# Metal-triggered topology switching in bipyridine-modified DNA G-quadruplexes

**DOI:** 10.1093/nar/gkag738

**Published:** 2026-07-28

**Authors:** Armin Durmisevic, Joseph Openy, Aatikah Majid, Mrunal Nanda, Ramon Vilar, Guido H Clever

**Affiliations:** Department of Chemistry and Chemical Biology, TU Dortmund University, Otto-Hahn Str. 6, 44227 Dortmund, Germany; Department of Chemistry and Chemical Biology, TU Dortmund University, Otto-Hahn Str. 6, 44227 Dortmund, Germany; Molecular Sciences Research Hub, Department of Chemistry, Imperial College London, 82 Wood Lane, London W12 0BZ, United Kingdom; Department of Chemistry and Chemical Biology, TU Dortmund University, Otto-Hahn Str. 6, 44227 Dortmund, Germany; Department of Biochemistry, University of Winsconsin-Madison, Madison, WI 53706, United States; Molecular Sciences Research Hub, Department of Chemistry, Imperial College London, 82 Wood Lane, London W12 0BZ, United Kingdom; Department of Chemistry and Chemical Biology, TU Dortmund University, Otto-Hahn Str. 6, 44227 Dortmund, Germany

## Abstract

Gaining control over DNA G-quadruplex topology bears potential to modulate and study their interaction with other biomacromolecules such as proteins. To achieve this, we introduced bipyridine ligands into short oligonucleotide strands derived from telomeric regions of humans and *Tetrahymena* as well as into an oncogenic promoter region capable of forming such G-quadruplexes. This modification makes it possible to dynamically access different G-quadruplex topologies through the formation of chelate complexes within the quadruplex loop regions using metals such as Cu^2+^, Ni^2+^, Zn^2+^, Co^2+^, and Cd^2+^. The metal-coordinated systems show enhanced stability towards thermal denaturation as well as a solvation-related response in the presence of molecular crowding reagents. Interestingly, these G-quadruplexes modified with bipyridine-metal complexes stay folded *in cellulo* and therefore show potential for creating new oligonucleotide-based diagnostic agents and therapeutics. Metal-stabilized G-quadruplexes could therefore be suitable as probes to explore protein-G4 interactions, as inducers for G4-dependent cellular processes, or decoys to sequester transcription factors.

## Graphical Abstract

**Graphical Abstract:**
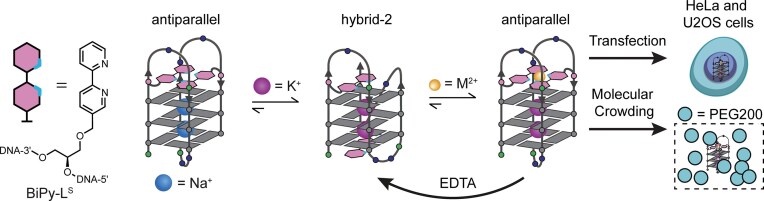

## Introduction

Guanine-rich oligonucleotides can fold into tetra-stranded structures known as G-quadruplexes (often abbreviated as G4 or GQ). There is significant evidence showing that G-quadruplex DNA plays a crucial role in various cellular processes, including the regulation of gene expression and the maintenance of chromosomal integrity [1, 2]. Due to their regulatory function, G-quadruplexes have been identified as promising drug targets in various areas such as cancer and neurodegenerative diseases [3–7]. Various small molecules, usually characterized by flat, extended π-surfaces and positive charges, have been found to bind and stabilize G-quadruplexes, thus serving as potential drug candidates [8–11]. Structurally, G-quadruplexes are composed of π-stacked layers of Hoogsten-type hydrogen-bonded guanine quartets, folded from G-rich DNA sequences and stabilized by monovalent metal cations occupying a central channel between the nucleobases. Depending on the strand molecularity (uni-, bi-, tri-, or tetramolecular), G-count, glycosidic bond torsion angles, and interstitial nucleotides, they show a high topological variety [12–15]. This extensive structural diversity is further influenced by the central metal cation (Li^+^, Na^+^, and K^+^), the solvation environment (including conditions of molecular crowding), and the presence of non-covalent binders, leading to different G-tetrad stacking patterns, strand polarities, and loop orientations [16, 17]. Common G-quadruplex topologies are divided into three categories, hybrid, parallel, or antiparallel, which can be easily differentiated through their distinct signatures in circular dichroism (CD) spectroscopy [18–20]. This polymorphism is crucial in defining the selective interaction of G-quadruplexes with regulatory proteins, and consequently, their cellular functions. Therefore, it offers a playground for the construction of new tailor-made G-quadruplexes with topologies and functions aimed at the targeted interaction with proteins of interest. Furthermore, chemical modification of such G-quadruplexes opens potential to control their structure and action by external stimuli [21–26].

A promising approach to exert control over G-quadruplex folding and introduce triggerable functions is to combine their polymorphism with the concept of metal-base pairing, where artificial ligands, covalently inserted into the DNA strand, can coordinate transition metal cations with structure-stabilizing properties. While numerous reports describe this phenomenon in duplex DNA [27, 28], only a few examples exist for other DNA secondary structures such as triplexes [29–31] and three-way junctions [32–36]. For G-quadruplexes, the few known reports include Hg^2+^–thymine interactions within a loop [37, 38] and 2,2′-bipyridine-containing linkers in the loops of bimolecular G-quadruplexes that lead to metal-mediated G-wire formation [39].

Previously, we have introduced a series of transition metal-base tetrads incorporated into uni- and tetramolecular G-quadruplexes, based on the square-planar coordination of four monodentate pyridine or imidazole donors to divalent cations such as Cu^2+^, Ni^2+^, and Zn^2+ ^[40–44]. We applied these systems in the construction of rigid EPR spin labels (exploiting the unpaired electrons of coordinated Cu^2+^ cations), the enantioselective catalysis of a Michael addition, and the metal-dependent activity switching of a of G-quadruplex-hemin complex serving as peroxidase mimic [41, 45–47]. While some of these systems already indicated the potential of increasing thermal stability through metal binding (and even a metal-triggered topological change could be observed [40]), the use of separate monodenate donors for the placement of tetra-coordinated metal complexes within the relatively short synthetic oligonucleotides (usually ≤25 bases) required chemical modification at four different sequence positions, thus risking significant structural disruption of the parental unmodified motif. We therefore now designed a 2,2′-bipyridine-based ligand-modified nucleoside (“ligandoside”) whose backbone architecture is similar to the glycol-based DNA surrogate introduced by Meggers (termed “GNA”) [48], thus allowing a facile synthesis of the respective phosphoramidite and enabling the incorporation of the ligand at any position during the DNA synthesis (5′, 3′, or internal). This new building block differs from previously reported, backbone-embedded chelate ligands, e.g. by Sugimoto [39], Sleiman [49], and us [50], as it is only connected to the strand via a one-point attachment. While it shares this feature with other previously introduced chelate ligands, e.g. a 2′-ribose-appended bipyridine by Shionoya [35], and some purine and pyrimidine deoxyribonucleotide-derived ligandosides by Switzer [32, 51], our herein introduced design benefits from a lean and easily accessible backbone that allows a flexible alignment of ligands in the G-quadruplex loop. Furthermore, bipyridines offer an enhanced complex stability as compared to monodentate pyridine ligands. They accept a broad spectrum of transition metal cations, as previously shown when incorporated into duplex DNA [32, 33, 52].

The work reported herein is aimed at exploring the impact of covalently incorporated bipyridine ligands on G-quadruplex topology and function. We describe the metal-binding capabilities of a series of modified G-quadruplexes and explored their effects on secondary structure stabilization and metal-responsive topology switching. We also report on their behaviour under varying solvation environments and within a cellular context.

## Materials and methods

### Chemistry

Chemicals and solvents were purchased from Sigma–Aldrich, Acros Organics, Carl Roth, TCI Europe, VWR, ABCR, or other suppliers and used as received without further purification. Ultrapure water (type I, 18.2 MΩ cm) was produced with a VWR Puranity TU 3 UV apparatus. Reactions involving substrates or reagents sensitive towards ambient air and/or hydrolysis were carried out using Schlenk techniques under argon. Purification was done by column chromatography with silica gel 60 from Macherey-Nagel (particle size: 40–63 μm). Additional purification was performed via gel permeation chromatography on Japan Analytical Industry LC-9210 II NEXT or Labo Ace LC-5610 systems with either HPLC-grade ethyl acetate or chloroform. NMR measurements were conducted at 298 K on Avance-500neo, 600, and 700 instruments from Bruker. Chemical shifts for ^1^H and ^13^C are reported in ppm with signals referenced to the residual solvent peak (signal multiplicity for ^1^H-NMR spectra: s: singlet, d: doublet, t: triplet, dd: doublet of doublets; dt: doublet of triplets; m: multiplet, and br: broad). High-resolution electrospray ionization mass spectrometry (ESI HRMS) was performed on a Bruker timsTOF ESI mass spectrometer.

### UV-Vis analysis

For all UV-VIS-based thermal denaturation studies, G-quadruplex samples contained 4 μM single-stranded DNA (= 4 μM G-quadruplex), 100 mM KCl or NaCl, 10 mM HEPES buffer pH 7.2, and, if present, 4–8 μM M^2+^ (Cu, Ni, Zn, Co, and Cd). For all experiments, samples were prepared with ultrapure water, heated to 90°C for 10 min, slowly cooled to 4°C with a cooling rate of 0.5°C/min, and then left at this temperature for several hours (typically overnight). Further details can be found in the SI.

### CD spectroscopy

For standard CD measurements, samples were prepared in the same way as for the UV-VIS-based thermal denaturation studies. CD spectra were measured on an Applied Photophysics Chirascan qCD spectropolarimeter (350–205 nm, 1.0 s time-per-point, step size 1 nm, bandwidth 0.5 nm, 3 repeats) at 25°C unless otherwise indicated. Temperature was controlled using a Quantum Northwest temperature control attached to a sample probe. The background was measured in the same cuvette as the sample. All spectra were averaged, background-corrected (cuvette, buffer, and electrolyte), smoothed (Savitzky–Golay, window size 5), and zeroed to the signal at 350 nm. Further details can be found in the SI.

### Molecular dynamics simulations

MD simulations were carried out as previously described [40, 43] using the Gromacs 2019.2 program [53–55] with the AMBER force field ff99bsc1 [56, 57] for the nucleic acid parts. A detailed explanation can be found in the SI.

### Cell assays

U2OS and HeLa cells were plated (1 × 10^5^ cells per well, 200 μl, 0.8 cm^2^) 24 h before each experiment and grown in Dulbecco’s modified Eagle’s medium (DMEM) media (Gibco) supplemented with 10% fetal bovine serum (FBS). To measure cellular toxicity of the samples, methylthiazolysulfophenyl tetrazolium (MTS) assays were conducted using the Promega CellTiter 96 Aqueous One Solution Cell Proliferation Assay kit, following manufacturer’s instructions. Cells were incubated with DNA samples over 6 h at varying concentrations from 5 to 2000 nM. Absorbance of the MTS reagent was then measured at 490 nm. The experiment was conducted in triplicate.

Confocal images were acquired using a Leica SP5 II confocal microscope after incubation of U2OS/HeLa cells with the DNA samples for 16 h. Images were taken with a 100× oil-immersion objective (correction collar, NA = 1.2, Leica) after excitation using an internal Ar+ laser at 495 nm and detection at 500–700 nm.

DNA transfection was performed with Lipofectamine 2000 (Invitrogen) following the manufacturer’s instructions: dye-labelled G4 DNA was incubated with Lipofectamine at a 3:1 ratio of Lipofectamine:DNA in Opti-MEM media (Gibco) for 15 min. The Lipofectamine/DNA mixture was then incubated with cells for up to 24 h and imaged every 4 h using a Sartorius Incucyte S3 Live-Cell Analysis System, from which the total green count of the selected cells was plotted over time to determine the optimal incubation time after which no more signal enhancement was present. All cell imaging experiments are an average of at least two independent biological repeats. Instances where images of three biological repeats were used are highlighted in relevant figure captions.

### Statistical methods

Association constants (K) and their associated errors were obtained from the Benesi–Hildebrand method by least-squares linear regression of *n* = 22 data points per condition. *K* values are reported as value ± standard error (SE), with 95% confidence intervals (CIs) calculated from the regression fit. Linear regressions were performed using OriginLab software. Observed rate constants (*k*_obs_) were determined by least-squares linear regression of the linear portion of the decay curve, assuming first-order kinetics. Each condition was measured in *n* = 3 independent replicates, and *k*_obs_ values are reported as mean ± standard deviation (SD) across replicates. Activation parameters (Δ*H*‡ and Δ*S*‡) were determined from the Eyring–Polanyi equation by unweighted least-squares linear regression of ln(*k*_obs_/T) versus 1/T across *n* = 4 temperatures. Errors in Δ*H*‡ and Δ*S*‡ were derived from the SEs of the slope and intercept, respectively, and are reported as value ± SE with 95% CIs. Linear regressions were performed using OriginLab software. Cell viability was assessed in *n* = 3 independent biological replicates per condition. Absorbance values were normalized to negative (full dehydrogenase activity) and positive (DMSO, no dehydrogenase activity) controls and are reported as mean percentage viability ± SD. No formal statistical comparisons between conditions were performed. Fluorescence intensity (count) in Förster resonance energy transfer (FRET)-based fluorescence microscopy experiments was calculated from *n* = 3 technical replicates and given as a mean value without error.

## Results and discussion

### G-quadruplex structure is modulated by transition metal binding

**Figure 1.**
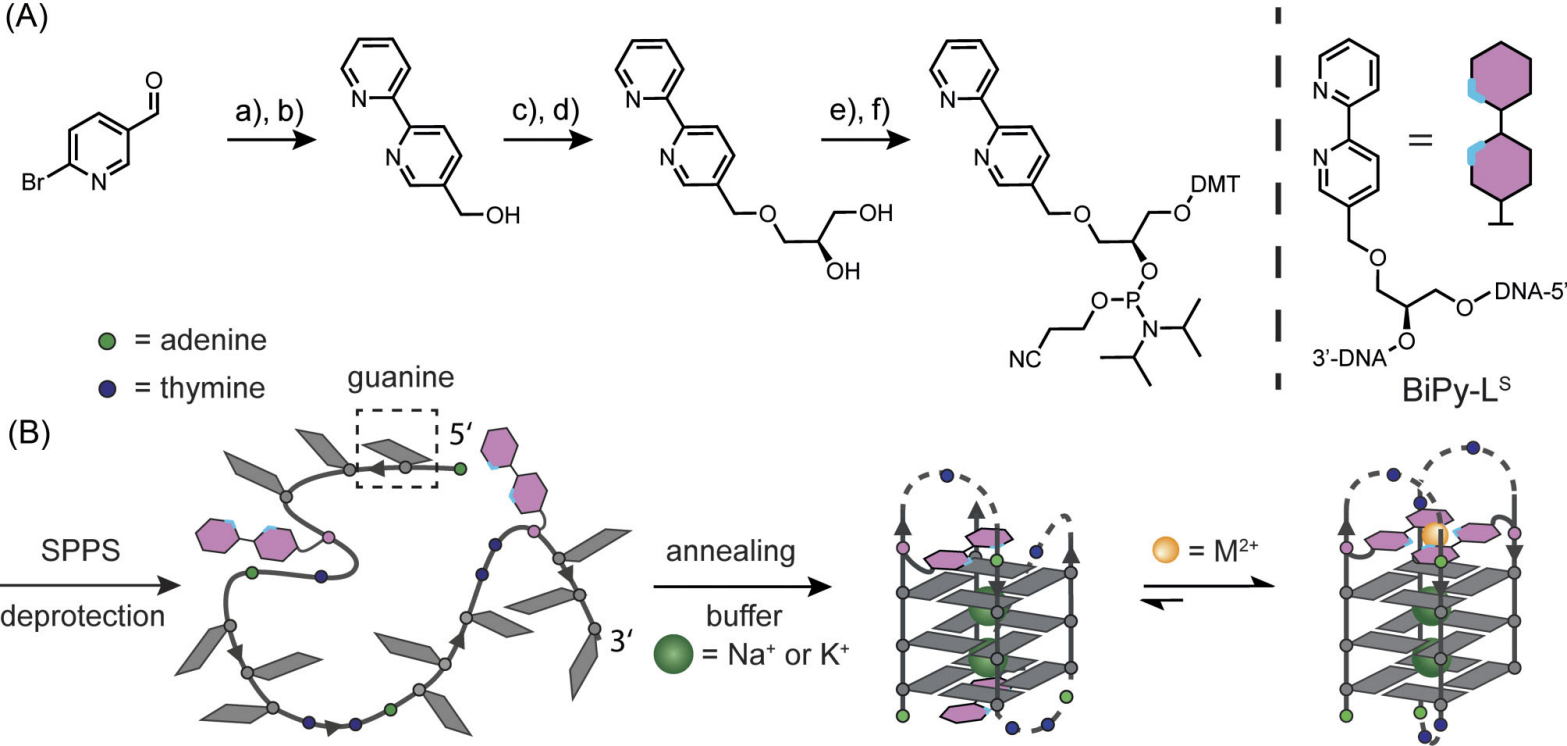
**A**) Synthesis of BiPy-L ligandoside starting from 6-bromonicotinaldehyde. (a) 2-(tributylstannyl)pyridine, Pd(PPh_3_)_2_Cl_2_, *m*-xylene, 110°C, 16 h; (b) NaBH_4_, THF, r.t., 24 h; (c) NaH, (*R*)-(2,2-dimethyl-1,3-dioxolan-4-yl)methyl 4-methylbenzenesulfonate, DMF, r.t., 24 h; (d) TFA, THF/water (1:1), 70°C, 3 h; (e) DMT-Cl, DIPEA, DMAP, THF, r.t., 24 h; and (f) CEP-Cl, DIPEA, THF, r.t., 3 h. (**B**) SPPS of various G-quadruplex-forming oligonucleotides, followed by annealing in buffer solutions containing either Na^+^ or K^+^ ions.

In order to incorporate the bipyridine ligandoside into an oligonucleotide strand, first a synthetic route had to be devised to obtain the necessary phosphoramidite building block (BiPy-L). As only few unsymmetrically substituted bipyridines are commercially available, the synthesis started with a Stille-cross coupling of 6-bromonicotinaldehyde and 2-(tributylstannyl)pyridine. The [2,2′-bipyridine]-5-carbaldehyde was then reduced to the alcohol, which was coupled to the glycidyl backbone via a nucleophilic substitution. After acid-promoted deprotection of the 1,3-dioxolane the necessary dimethoxytrityl (DMT) protecting group could be installed at the primary hydroxyl group. The phosphitylation followed reported procedures [40]. The glycol-based synthetic backbone was chosen as it was synthetically simple and offered a high degree of structural flexibility for the incorporated ligand. All synthetic steps were confirmed by NMR spectroscopy (SI, Supplementary Figs S1–S6). With the ligand at hand, solid-phase phosphoramidite synthesis (SPPS) was performed (Fig. 1).

In a first design, several sequences known to fold into unimolecular DNA G-quadruplexes, derived from the human telomeric region (htel), the promoter region of the Myc oncogene (pu22), and from the *Tetrahymena* telomeric region (ttel), were site-specifically modified with two bipyridine ligandosides each. Positions within the strands were decided based on the analysis of known structures of the parental sequences, together with experience from our previous studies on the incorporation of monodentate ligands [40, 41, 44–47, 58]. Therefore, selected nucleotides either within loop regions of the G-quadruplexes or guanines within a terminal tetrad were exchanged for BiPy-L. As pu22-derived parallel quadruplex topologies do not offer any closely neighbouring loops, we placed BiPy-L before and after the first and last run of guanines, respectively, and in addition elongated the first loop by up to two bases to increase structural flexibility. Eventually, the overall goal is to combine minimal disturbances with respect to the initial structure with optimal ligand positioning for the successful formation of metal complexes, not only to achieve a metal-mediated increase of thermal stability but also to exert stimuli-responsive control over G-quadruplex topology. It is important to note that the three parental sequences studied herein differ not only in their G-quadruplex topology but also in loop composition and length, and that the modification strategy is not identical across all constructs. Consequently, certain observations—in particular the quantification of thermal stability and the sensitivity to metal coordination—are expected to be scaffold-dependent and should not be uncritically transferred between systems. In contrast, the fundamental principles underlying the metal–BiPy complex formation, including coordination geometry and the spin-state-dependent fluorescence behaviour, are intrinsic to the bipyridine ligand and the respective metal ions and are therefore expected to be generally applicable regardless of the specific scaffold.

Successful synthesis and purity of modified G-quadruplex sequences were confirmed by analytical HPLC (SI, Supplementary Figs S7–S18) and LC-ESI-MS (SI, Supplementary Figs S19–S41). Modified sequences were investigated by thermal differential spectra (TDS), UV-Vis melting analysis, and CD spectroscopy after annealing in absence or presence of divalent metal cations and different buffers containing either K^+^ or Na^+^. All six synthesized sequences (Table 1) showed a characteristic minimum at 295 nm and maxima at approx. 243 and 273 nm in their respective TDS spectra, indicative of successful G-quadruplex formation (SI, Supplementary Figs S52–S61) [59].

**Table 1.** Bipyridine-modified DNA sequences

Name	Sequence (5′–3′)
htel22^(a)^	A GGG TTA GGG TTA GGG TTA GGG
htel22-L2a	A GGG L^(b)^TA GGG TTA GGG TTL GGG
FAM-htel22-L2a	FAM^(c)^-A GGG LTA GGG TTA GGG TTL GGG
FAM-htel22-L2a-Cy3	FAM-A GGG LTA GGG TTA GGG TTL GGG-Cy3^(c)^
htel22-L2b	A GGL TTA GGG TTA GGL TTA GGG
FAM-htel22-L2b	FAM-A GGL TTA GGG TTA GGL TTA GGG
FAM-htel22-L2a-Cy3	FAM-A GGL TTA GGG TTA GGL TTA GGG-Cy3
ttel24^(a)^	TTG GGG TTG GGG TTG GGG TTG GGG
ttel24-L2a	TTG GGL TTG GGG TTG GGL TTG GGG
ttel24-L2b	TTG GGG TTL GGG TTG GGG TTL GGG
pu22^(a)^	TGA GGG TGG GTA GGG TGG GTA A
pu22-L2a	TGL GGG TT^(d)^GG GTA GGG TGG GLA A
pu22-L2b	TGL GGG TTAGG GTA GGG TGG GLA A

(a) DNA sequences of parental G-quadruplexes for the modified systems; (b) incorporated BiPy-L ligandosides indicated by L; (c) FAM = carboxyfluorescein, Cy3 = cyanine3; (d) nucleotides added in addition to the parental sequence are underscored.

This also held true when annealed in the presence of a divalent metal source, where only minor changes between G-quadruplexes with and without M^2+^ could be identified, such as an additional band at 315 nm in the TDS spectra. This additional band stems from the redshift of the incorporated bipyridine ligands when forming a corresponding metal chelate complex (SI, Supplementary Figs S52–S61) [52]. This marked a first indication for the successful formation of a transition metal complex within the G-quadruplex. The region between 265 and 220 nm in the TDS spectra showed some differences between the individual sequences, originating from their different loop compositions and adopted topologies. With first spectroscopic evidence for the complexation of Cu^2+^, Ni^2+^, Zn^2+^, Co^2+^, and Cd^2+^, we next investigated the potential thermal stabilization by the respective metals. UV-Vis melting curve analysis revealed that even in the absence of transition metal cations, the bipyridine modifications have an overall positive impact on the stability of the respective quadruplexes, as the *T*_m_ values were found to be similar or above those of the parental sequences (Table 2). Furthermore, a large thermal stabilization could be observed for many of the modified sequences when a divalent metal ion was added. Ni^2+^ and Zn^2+^ exerted the strongest effects, with an increase of up to Δ*T*_m_ = +31 K in the case of htel22-L2b. The lowest stabilization was achieved for the Myc-derived sequences pu22-L2a and -L2b where *T*_m_ values were already above 80°C without the formation of a metal complex (SI, Supplementary Figs S62–S71). Overall, a strong influence of the formation of bipyridine metal complexes on the G-quadruplex stability could be observed, rendering these artificial DNA constructs largely resistant to thermal denaturation.

**Table 2.** Melting temperatures of bipyridine-modified DNA sequences in presence and absence of various transition metal cations, measured by UV-Vis spectroscopy

Name	*T*_m_ [°C]	*T*_m_ (Δ*T*_m_) (+Cu^2+^)	*T*_m_ (Δ*T*_m_) (+Ni^2+^)	*T*_m_ (Δ*T*_m_) (+Zn^2+^)	*T*_m_ (Δ*T*_m_) (+Co^2+^)	*T*_m_ (Δ*T*_m_) (+Cd^2+^)
htel22	64	n.d.	n.d.	n.d.	n.d.	n.d.
htel22-L2a	68	79 (+11)	84 (+16)	83 (+15)	81 (+13)	83 (+15)
htel22-L2b	44	72 (+28)	75 (+31)	74 (+30)	74 (+30)	71 (+27)
ttel-24*	n.d.	n.d.	n.d.	n.d.	n.d.	n.d.
ttel24-L2a	65	75 (+10)	77 (+12)	77 (+12)	73 (+8)	76 (+11)
ttel24-L2b	65	76 (+11)	79 (+14)	77 (+12)	71 (+6)	74 (+9)
pu22	85	n.d.	n.d.	n.d.	n.d.	n.d.
pu22-L2a	37 + >85**	>85	>85	>85	n.d.	n.d.
pu22-L2b	>85	>85	36 + >85**	37 + >85**	n.d.	n.d.

* Poorly defined melting transition. ** Two melting points identified [67].

Binding constants for the chelate metal complexes were determined via UV-Vis titration experiments by stepwise (0.1 eq) addition of a solution of metal source, incubation for 10 min, and subsequently following the increase of the 315 nm band. All sequences showed very high binding constants in the range of 10^10^‒10^11^ M^−1^. For the calculations, we treated the two incorporated bipyridines as a single tetradentate ligand as they are covalently attached to each other through the DNA strand. This characteristic will serve as an important factor for creating functional systems resistant to harsher conditions within a cellular environment (SI, Supplementary Fig. S72, Supplementary Table S2).

**Figure 2.**
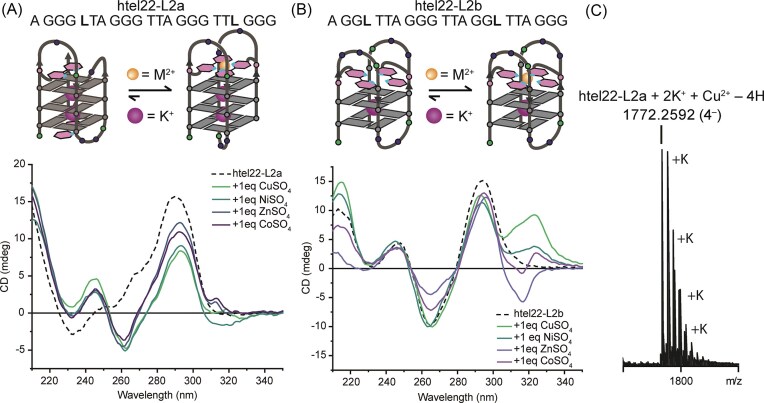
CD spectra of bipyridine modified sequences (**A**) htel22-L2a and (**B**) htel22-L2b in absence (dotted line) and presence (coloured lines) of divalent metal cations. While htel22-L2a changes from a hybrid-2 to an antiparallel topology upon metal addition, htel22-L2b is already prefolded into an antiparallel topology and shows no refolding upon metal complexation. (**C**) Native mass spectrometry measurements further confirm selective binding of one transition metal cation by the modified G-quadruplexes (expected mass for htel22-L2a + 2K^+^+Cu^2+^-4H: 1772.2583, measured mass: 1772.2592).

As we chose parental sequences known to adopt a variety of possible quadruplex topologies, and even show polymorphic behaviour, we then probed how the artificial ligandosides and their coordination with divalent metal cations affect the quadruplex topology. Out of the six synthesized sequences, the most notable behaviour could be observed for htel22-derived sequences, found in the human telomeric region. Two design approaches were followed here. (i) Replacement of loop nucleotides with the BiPy-L ligandosides aimed towards minimal disturbance of the initial structure, followed by formation of the metal complex within the quadruplex loop region. (ii) Replacement of guanines aimed towards the formation of a tetrad mimic whereby two bipyridines (chelating a metal cation) take the stabilizing and structural role of a natural G-tetrad. In the case of htel22-L2a, where two bipyridine ligandosides are incorporated into the loops at positions #5 and #19 (in 100 mM KCl-containing buffer), the formation of a hybrid topology—similar to the parental sequence—was observed, indicating no large structural impact of the modifications. In contrast, for htel22-L2b, where two guanines at positions #4 and #16 were replaces, a fully antiparallel topology was obtained (Fig. 2A and B).

As htel22 is known for its polymorphic behaviour [61, 62], the possibility of tuning its topology through metal coordination seemed intriguing. After annealing sequence htel22-L2a in the presence of a divalent metal cation (Cu^2+^, Ni^2+^,or Zn^2+^), CD analysis revealed the adoption of a new antiparallel topology with characteristic maxima at 290 and 240 nm and a minimum at 260 nm. Similar to the TDS spectra, an additional band at 315 nm could be identified, confirming the formation of the metal complex within a chiral environment. We therefore assume that the two spatially separated bipyridines form a stable metal complex during the annealing process and consequently force the DNA to adopt a folded topology that can accommodate this complex on one side of the G-stack. Compared to our previous results [42], based on monodenate ligands, this complexation-induced refolding was here not only achieved with Cu^2+^ but also a variety of other transition metal cations such as Ni^2+^, Zn^2+^, Co^2+^, and Cd^2+^, owing to the coordination versatility of the bipyridine ligand. On the other hand, heavier metal cations (with slower ligand exchange kinetics, e.g. Pd^2+^, Pt^2+^, Ag^+^, and Au^3+^) or those strictly requiring an octahedral coordination geometry (such as Fe^2+/3+^, Ru^2+/3+^, and Rh^3+^) could not be employed for the topological control of htel22-L2a, comprising two bidentate ligands. In NaCl-containing buffer, the topology of htel22-L2a is not clearly identifiable, as the initial structure has characteristics of an antiparallel topology with slight shifts in the minimum from 260 to 280 nm (whether the strand in the presence of sodium forms a mixture of different topologies or an unexpected folding pattern could not be unambiguously characterized). Here, addition of M^2+^ did not lead to any refolding, as indicated by only minor changes in the CD spectra upon metal addition (SI, Supplementary Fig. S42).

When the bipyridines were not placed within the loops of the sequence but replaced two guanines at positions #4 and #16, a replacement for a natural G-tetrad could be formed as seen for modified sequence htel22-L2b. We therefore left the two guanines of the parental tetrad in positions #8 and #20 unchanged. Due to then only two full G-tetrads being present, this quadruplex always adopts an antiparallel topology, indifferent to either KCl or NaCl in the buffer or the addition of M^2+^. Here, the two bipyridines already sit in close proximity to each other and are therefore not able to induce any refolding of the structure when coordinating the transition metal cation (Fig. 2B and Supplementary Fig. S43).

The sequences derived from *Tetrahymena* or the Myc oncogene promoter formed G-quadruplexes of different topologies as shown by their respective CD signatures (SI, Supplementary Figs S44–S47). In the case of ttel24-L2a and ttel24-L2b in potassium-containing buffer, the initially folded structures were more complex as they consist of a mixture of different topologies, not clearly distinguishable. Although these structures did show a partial refolding in presence of M^2+^ ions, they most likely remained mixtures of topologies. This indicated that metal complexation in this case only slightly affects the equilibria between almost equally populated structures. While in Na^+^ containing buffer, both ttel24-L2a and -L2b showed no distinct G-quadruplex signature, addition of M^2+^ cations triggered formation of an antiparallel topology (SI, Supplementary Figs S44 and S45).

The Myc-derived sequences pu22-L2a and -L2b adopt fully parallel topologies with a maximum at 260 nm and a minimum at 240 nm, identical to their parental sequence. Here, neither the addition of M^2+^ nor the change from K^+^ to Na^+^-containing buffer affected the observed structure. This behaviour is in line with their extremely high thermal stabilities (Table 2) as well as the general consideration that—wherever their formation is feasible—parallel G-quadruplexes represent the thermodynamically most favourable topology. Still, the presence of minor bands at ~315 nm in the CD spectra as well as the TDS spectra, together with native mass spectrometry measurements, allowed us also for these constructs to confirm the formation of the bipyridine metal complexes (with Cu^2+^, Ni^2+^, and Zn^2+^) (SI, Supplementary Fig. S46 and S47). Overall, all six sequences tolerated the BiPy-L modification well, successfully folding into G-quadruplex topologies of all three major structural types (or mixtures thereof). Although all six sequences showed successful metal complexation with varying degrees of stabilization, only htel22-L2a showed a metal dependent switching where both accessible states had clearly identifiable topologies, making it the most interesting system for further investigations.

### MD simulations

**Figure 3.**
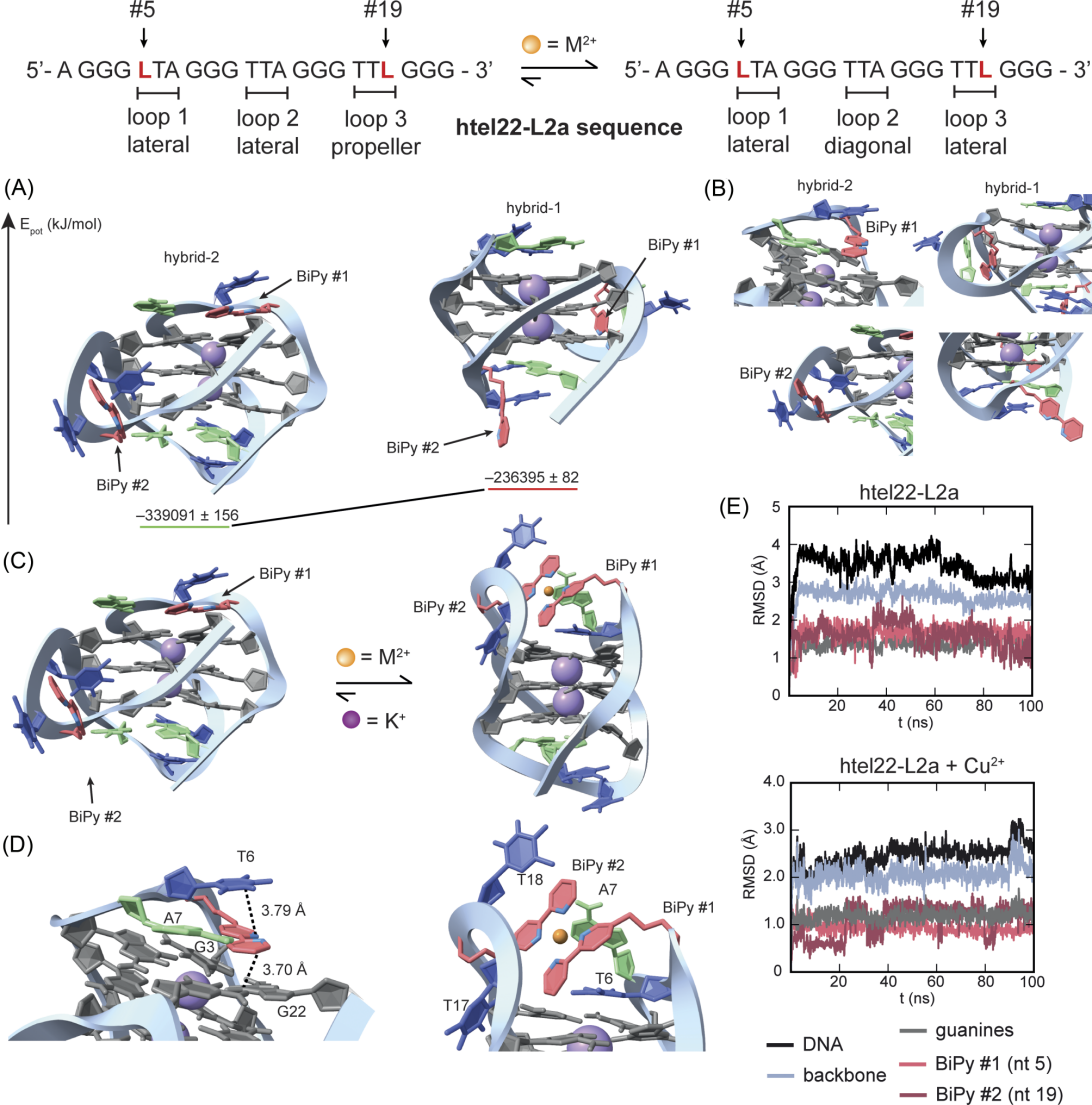
(**A**) Comparison of potential energies for the modelled structures for htel22-L2a in either a hybrid-2 (parental starting structure adopted from PDB: 2JPZ) [63] or hybrid-1 (PDB: 2HY9) [64] topology. (**B**) Closer examination of BiPy-L, positioned in the two different hybrid topologies, reveals π-stacking of the bipyridine unit in the hybrid-2 (3 + 1) structure, absent in hybrid-1 (1 + 3). (**C**) Models of htel22-L2a in hybrid-2 topology (left) and after refolding into the observed metal-induced antiparallel topology (right). (**D**) Closer examination of the interactions of BiPy #1 with the G-tetrad and the thymine T6, leading to additional stabiliszation of the metal-free structure (left). In contrast, the bipyridine metal complex does not lie flush with the G-tetrad underneath and shows no additional π-stacking or CH–π interactions with surrounding nucleobases (right). (**E**) Comparison of RMSD values of individual components of the models for htel22-L2a in the presence and absence of Cu^2+^ stabilization.

In order to better understand these new bipyridine-modified G-quadruplexes and their assumed topologies, we performed a series of molecular dynamics (MD) simulations for each system. Our goal was to compare the positioning of the BiPy-ligandosides and the corresponding metal complexes and identify potential interactions with other nucleotides (Fig. 3). All systems were run in the Gromacs program with the AMBER force field over 100 ns at 298 K, with 100 mM KCl in a periodic rhombic dodecahedron box with a cutoff at 1.5 nm. All systems reached a stable state within the 100-ns simulation runs, at which root-mean-square deviation (RMSD) values remained consistent. Looking more specifically into the refolding of htel22-L2a, we first compared two starting structures for a hybrid-2 (“3 + 1”) and a hybrid-1 (“1 + 3”) topology in absence of any transition metal cations, modelled on the basis of the experimentally observed CD results (Fig. 3A). For the hybrid-2 structure, the bipyridine located in the first loop was found to stack in between the upper G-tetrad, hovering above G22 and G3 mainly, and the loop thymine T5. The RMSD of this bipyridine ligandoside was ~1.8 Å without strong deviations over 100 ns, indicating low structural flexibility. The distances between the aromatic rings of the ligandoside and the G22, G3, and T5 nucleotides ranged from 3.7 to 4.2 nm, which is well within the region for potential π–π interactions and would explain the slight increase in thermal stability of htel22-L2a over the wild-type sequence (delta *T*_m_ = +4°C) (Fig. 3D). The constant RMSD values of the guanines and the backbone, as well as the whole artificial construct, further emphasize the stability of the htel22-L2a system. Interestingly, the bipyridine in the third loop shows an expected flexibility over the first 50 ns, but stabilizes afterwards, seemingly interacting with the neighbouring thymine of T18. We then compared second rounds of MD runs, initiated from various starting structures that were generated through manual alteration of the bipyridine ligand orientations, to check if the obtained structures can be taken as viable representations of the thermodynamic minima. Indeed, the newly obtained structures were found to be very similar to the initially observed ones in that the ligand towards the 5′ end tends to “swing back” and engage in π–π interactions with the G-tetrad and the thymine T5, while the bipyridine ligand towards the 3′ end rotates outwards and does not remain stacked underneath the lower G-tetrad. While the resulting conformations obtained from the starting structure variations are not perfectly superimposable, they are structurally very similar, and it can be assumed that the found arrangement indeed comes close to a reasonable minimum (SI, Supplementary Fig. S75).

For the alternative hybrid-1 starting structure of htel22-L2a, a more complex situation was encountered. The obtained RMSD values showed an overall stable structure over 100 ns, yet the orientation of the free bipyridine ligands was found to be very different. The first ligandoside is wedged between the G-tetrad and the phosphate backbone in a perpendicular fashion, creating a rather bulged propeller-type loop towards the 5′ end. The bipyridine ligand in the lateral loop towards the 3′ end is oriented away from the G-core and does not stack on top of the upper G-tetrad. Again, altered starting structures were created from the first MD run, able to show that the initially obtained structure was already very close to a robust minimum structure (for details see SI). Comparing the potential energies of the two systems, we obtained a significantly lower value for the 3 + 1 starting topology with *E*_pot_ = ‒339 091 ± 156 kJ/mol compared to that of the 1 + 3 starting structure at *E*_pot_ = ‒236 395 ± 82 kJ/mol. While these values can only be taken with caution as a rough basis for an energetic comparison, the number, type, and covalent connectivity of all atoms (including the DNA, electrolytes, and solvents) are identical for both modelled systems. We therefore conclude that htel22-L2a in absence of any metal adopts predominantly a hybrid-2 topology in accordance with CD spectroscopic results.

Next, we modelled the antiparallel topology ofhtel22-L2a—as inferred from the experimental results in the presence of a single M^2+^ cation, chelated by both bipyridine ligands on one side of the G-quadruplex stem. Positions of the individual bipyridine nitrogens coordinating to the metal center where not fixed in space as to allow free flipping of the two bipyridine ligands around the metal center during the simulation run. Restraints where only put on the relative orientation of the nitrogens with respect to the Cu(II) cations to make them obey a square-planar coordination geometry (that would otherwise not be properly kept by the force field approach). The system showed an overall remarkable stability with low RMSD values and only minor changes throughout the 100-ns simulation time. As expected, the ligandosides demonstrated an overall increase in structural stability in the newly formed metal complex, with very low RMSD values. When comparing the backbone flexibility in absence and presence of the metal complex, also an overall RMSD value decrease could be observed, nicely showing the metal-stabilizing effect on the entire G-quadruplex structural dynamics. Overall, the obtained calculation results are thus in accordance with the spectroscopic observations and thermal stability studies.

The modelled structures of the smaller htel22-L2b sequence, adopting an antiparallel basket-type topology, showed a distinct strain within the G-tetrads as the individual guanines are slightly off-plane twisted in a propeller-type arrangement around the central potassium cation. This is potentially caused by additional π–π interactions between the bipyridine ligandosides and the neighbouring nucleobases. The overall antiparallel topology is, however, maintained. In presence of the formed metal complex, the guanines adopt a flatter arrangement towards each other, representing a slightly less distorted G-quadruplex. Interestingly, during the simulation run, the orientation of the biypyridines towards each other changed from an initially expected (and manually modelled) *cis* orientation to a *trans* arrangement around the square-planar metal center (SI, Supplementary Figs S73 and S74).

### Refolding kinetics

**Figure 4.**
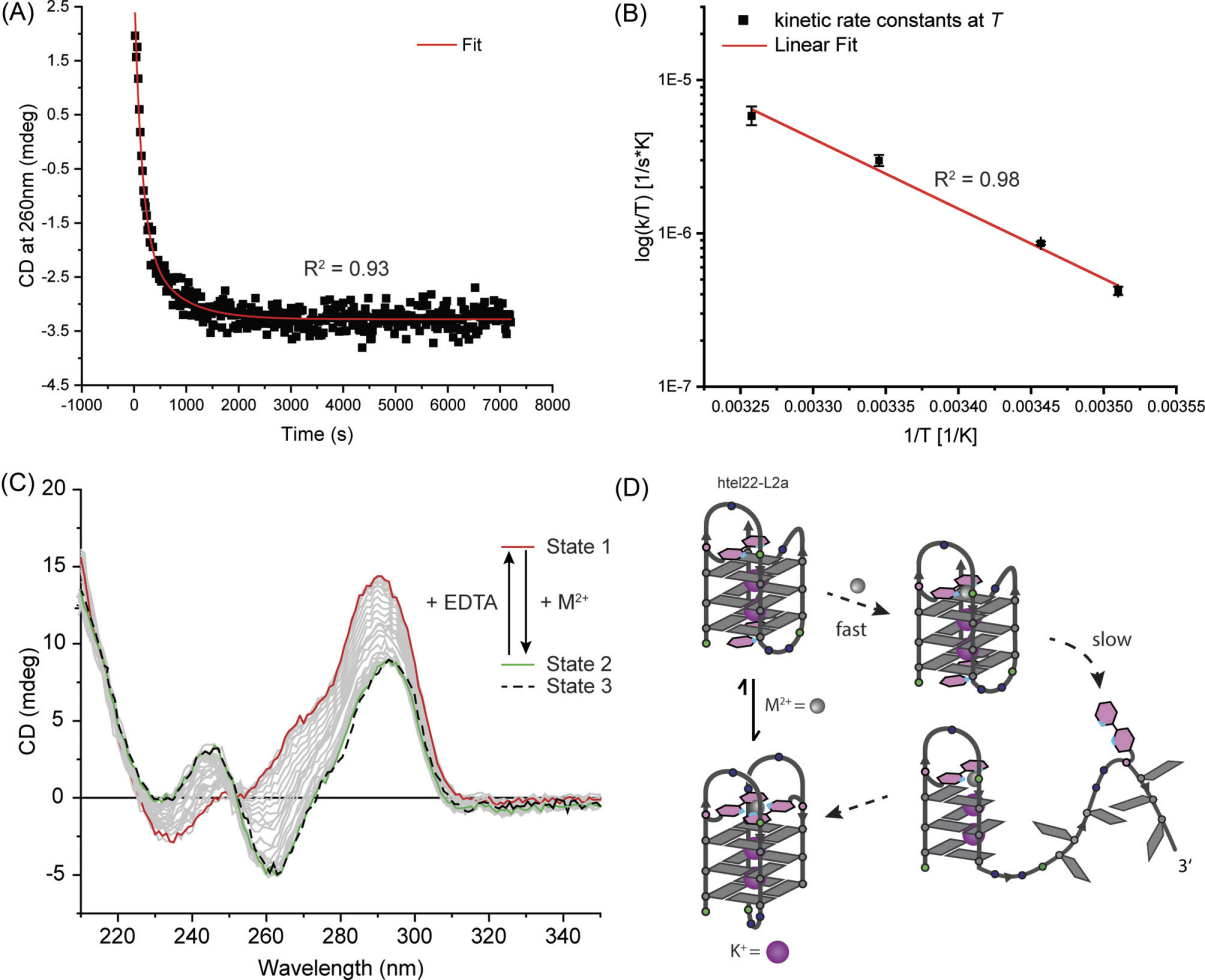
**A**) Decay of the CD signal of htel22-L2a at 260 nm over 7200 s at 306 K, measured in intervals of 10 s. (**B**) Eyring–Polanyi plot for the determination of enthalpy and entropy of activation and Gibbs activation energy. (**C**) CD spectrum of htel22-L2a showcasing the slow refolding from hybrid-2 (red line) to antiparallel topology (green line) upon addition of CuSO_4_ (grey lines). This process is reversible by ethylenediaminetetraacetic acid (EDTA) addition. For comparison, htel22-L2a annealed directly in presence of Cu^2+^ (dashed line). (**D**) Schematic representation of the suggested refolding mechanism, comprising the thermally induced (but low-probability) melting of the hybrid-2 form under slight steady-state population of a partially unfolded state that is then strongly driven to refold into the metal-stabilized, antiparallel product by formation of the chelate complex.

With the possibility of accessing multiple topologies through the formation of the bipyridine metal complexes in hand, we next wanted to investigate if this process could be induced after a DNA G-quadruplex had already been formed (by thermal annealing) and if the topological switch could be reversed. When 1 eq. of MSO_4_ (M = Cu, Ni, or Zn) was added to the pre-folded htel22-L2a quadruplex (4 µM DNA concentration, 100 mM KCl-containing HEPES buffer, 25°C), a slow but strong change in the CD signature could indeed be observed, showing the transition into an antiparallel topology after 1.5 h at 25°C (Fig. 4C). We note that this slow process, concomitant with significant changes in the CD spectra, strongly indicates a major refolding of the G-quadruplex (as in alternative cases, where metals bind into an already preorganized ligand environment, CD spectral changes are usually rapid and only of minor extent) [40]. Following this process by decay of the CD band at 260 nm over time and at various temperatures allowed us to identify a first-order reaction as well as determine its kinetic rate constants in the range of 10^−3^ to 10^−4^ s^−1^ (Fig. 4). Through Eyring–Polanyi plot analysis, the activation enthalpy (*ΔH^‡^*), entropy (*ΔS^‡^*), and the Gibbs energy of activation (*ΔG^‡^*) could be determined. A large positive enthalpic contribution of +37.9 kJ∙mol^−1^ was calculated, likely originating from the loss of stabilizing interactions (H-bonds) during the refolding process. The negative activation entropy of −80.2 J∙K^−1^∙mol^−1^ indicates that the system seems to retain a rather organized and folded state throughout the process, which aligns with similarly flexible systems which were described to refold via transitory ensembles without fully unfolding [65].

The resulting Gibbs activation energy of 61.8 kJ∙mol^−1^ at 298.15 K is in good accordance with reported values for other refolding processes of G-quadruplexes [65]. Apparently, refolding of htel22-L2a is eventually driven by the formation of the metal complex, and we suggest a mechanism going through a lowly populated, partially unfolded intermediate as depicted in Fig. 4D. The formation of the metal complexes (with Cu^2+^, Ni^2+^, and Zn^2+^) should—according to the typical coordination kinetics of these metals—be fast (10^3^–10^6^ s^−1^) [66], leaving the actual refolding of the quadruplex as rate-determining step, which is also demonstrated by the DNA concentration-dependent first-order kinetics observed for the process. Furthermore, when comparing the impact of the different metals, no large differences could be observed (SI, Supplementary Figs S76 and S77; Supplementary Tables S3 and S4). In order to revert the now antiparallel htel22-L2a quadruplex into its starting hybrid structure, metal decomplexation was tested. Although EDTA shows exceptionally large binding affinities for many transition metals [67, 68], the DNA-confined bipyridine complex showed to be stable to metal de-coordination after addition of up to 2 eq. of EDTA for 12 h. Only after 4 eq. of EDTA was added, a slow change of the CD signature was observed, leading to the reconstitution of the hybrid topology of htel22-L2a after 24 h (SI, Supplementary Figs S78 and S79). We additionally showed that refolding can occur undisturbed in presence of other chelating reagents such as spermin (SI, Supplementary Fig. S81).

### Refolding under molecular crowding conditions

**Figure 5.**
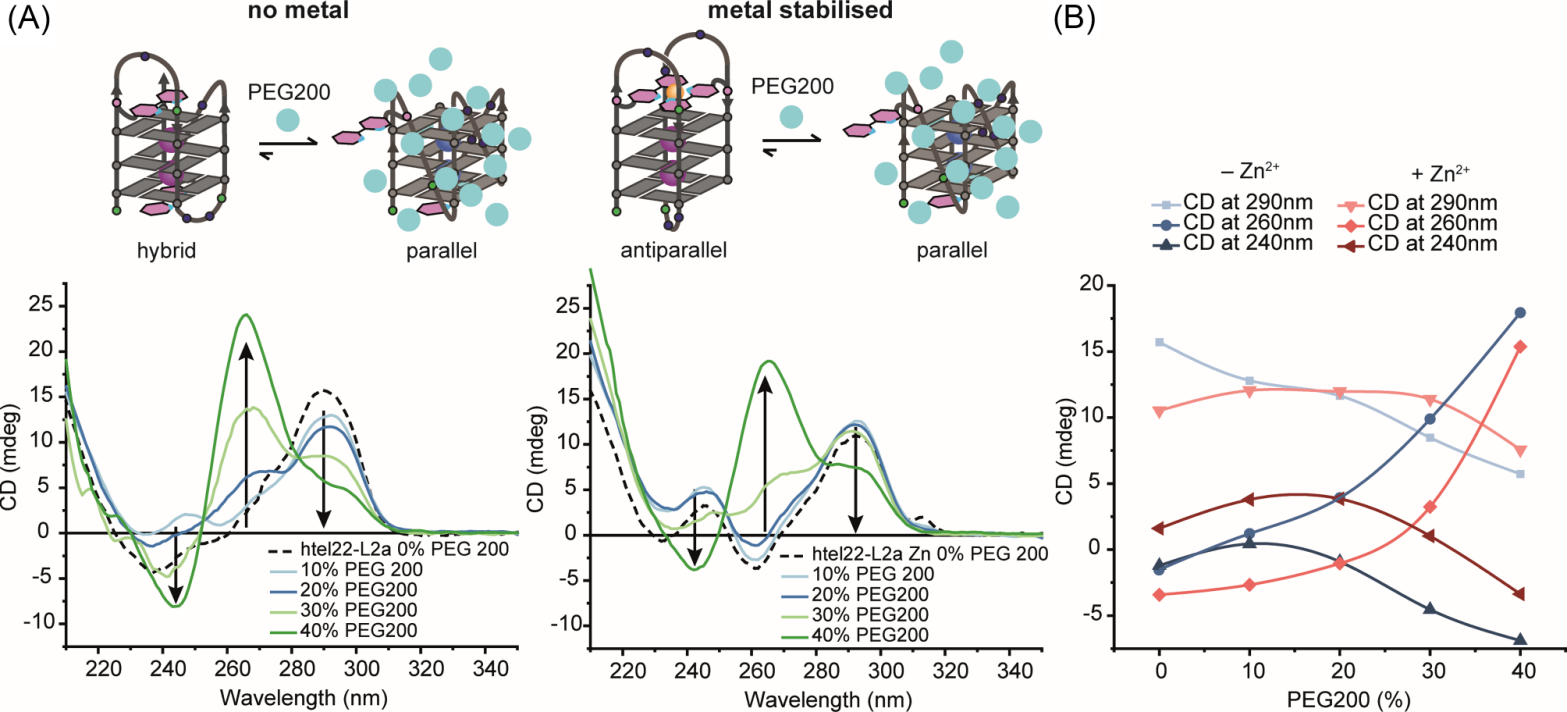
(**A**) CD spectra of htel22-L2a in increasing concentration of molecular crowding reagent PEG200 (10%–40%). PEG200 induces an earlier and stronger refolding of htel22-L2a into a parallel topology when not stabilized by the metal complex, as indicated most prominently by the CD signal at 290 nm. (**B**) Change of CD signal intensities of htel22-L2a in the absence and presence of Ni^2+^ at various wavelengths with increasing PEG200 concentration.

With these modified systems in hand, both capable of switching topologies as well as being stabilized by the formation of the bipyridine metal complexes, we then set out to explore them in a more biological context and solvation environment. The cellular environment is much more densely packed, with other bio- and small molecules exerting a crowding effect on the DNA, when compared to isolated *in vitro* conditions containing only water, buffer, and metal ions. For this reason, we first studied the system under molecular crowding conditions using PEG200, a common reagent capable of mimicking a cellular environment and known to have an impact on G-quadruplex topologies [17, 69]. When annealed in increasing concentrations of PEG200 (0%–40% w/t), the sequenceshtel22-L2a and htel22-L2b showed different behaviours (Fig. 5).

While htel22-L2a in the absence of stabilizing M^2+^ ions quickly refolds into a fully parallel G-quadruplex upon PEG addition, the samples in the presence of M^2+^ ions did not refold from antiparallel to parallel before the addition of up to 20% PEG200. Only after further increasing the PEG200 content did a change became noticeable. In the optical spectra, a large shoulder at 290 nm is still observable even at 40% PEG200, indicating an incomplete topological switch of the M^2+^-stabilized htel22-L2a quadruplex by the molecular crowding reagent (Fig. 5A and B).

PEG200 had an overall destabilizing impact on htel22-L2a without divalent cations, lowering its melting temperature by Δ*T*_m_ = –13 K with 20% PEG200 and by Δ*T*_m_ = –16 K at 30% PEG200, where a second higher melting point at *T*_m_ = 82°C could be observed, likely representative of the now parallel G-quadruplex. PEG200 had no large effect on the M^2+^-stabilized structures, independent of the concentration, lowering their melting temperatures by just Δ*T*_m_ = 1–2 K. No melting profile could be obtained for samples containing 40% PEG200. In contrast to this, htel22-L2b, incapable of adopting another topology, remains fully antiparallel even up to 40% PEG200 in the solution, either in presence or absence of stabilizing M^2+^ ions (Supplementary Fig. S80). This is comparable to similar G-quadruplexes, like those derived from TBA, where no refolding was observed [40]. Only small changes can be observed in the corresponding CD signatures, yet these do not indicate any major structural refolding.

### Transfection of G4s into living cells

Our long-term goal is to develop highly stabilized G-quadruplexes that can serve as potential ‘decoys’ for the cellular sensing and modulating of protein-quadruplex interactions. Towards this aim, and considering the high stability of the metal-stabilized G-quadruplexes herein reported, we sought to investigate their behaviour within a cellular environment, i.e. by establishing their cellular uptake, stability, potential toxicity, and localization within living cells by fluorescence microscopy. For this purpose, DNA oligonucleotides htel22-L2a and -L2b (carrying additional modifications as outlined below, Supplementary Figs S48–S51) were transfected into two human cancer cells lines, HeLa and U2OS. The modifications included either a single fluorescein (FAM) fluorescent label on the 5′-end of the oligonucleotide strand or a double fluorescent labelling with FAM and a cyanine-3 (Cy3) on the 5′- and 3′-ends, respectively, to perform FRET experiments to reveal the folding state.

Before conducting any imaging studies, it was first necessary to establish the viability of the cells in the presence of the modified G-quadruplexes (as indicated earlier, htel22-L2a and htel22-L2b were selected) under different conditions (i.e. with/without coordinated Ni^2+^ or Zn^2+^, and lipofectamine). To this aim, MTS assays were performed with U2OS and HeLa cells incubated with different concentrations of the oligonucleotides (with/without Cu^2+^, Ni^2+^, or Zn^2+^, and lipofectamine). As can be seen in Supplementary Figs S82 and S83, viability was >80% for both cell lines when incubated with 1 μM oligonucleotides (10 μl of a 10 µM oligonucleotide solution added to 100 μl of cells in DMEM media; final oligonucleotide concentration 1.0 μM) with/without Cu^2+^, Ni^2+^, or Zn^2+^, and lipofectamine. Increasing the concentration to 2 μM led to cell viabilities <80% (in some cases as low as 50%), and therefore it was decided to use 1 μM for subsequent studies since this provided high enough fluorescence signal to be easily detected by microscopy and good cell viability. The observed toxicity is well within the realm of oligonucleotide systems of similar molecular weight or nucleotide length reported for human cancer cell lines (SI, Supplementary Figs S82 and S83) [70].

**Figure 6.**
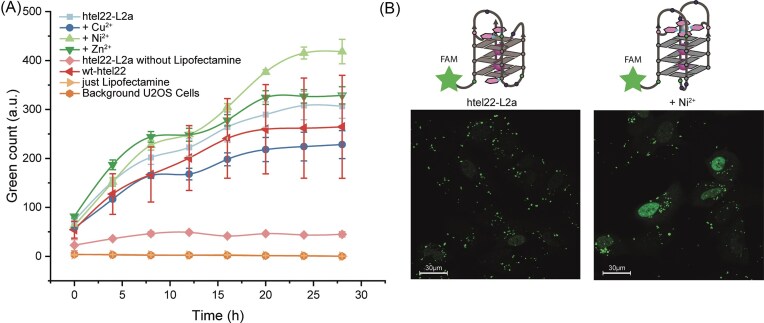
(**A**) Plot of recorded fluorescence of FAM-labelled, ligand-modified G-quadruplexes (in absence or presence of divalent metal cations) over time after transfection into U2OS cells, indicating a maximum yield of transfection after 16–24 h. (**B**) Confocal fluorescence microscopy images of transfected htel22-L2a in presence or absence of Ni^2+^. Localization of the Ni^2+^-stabilized G-quadruplex to the nucleus was found to be enhanced. No entering of the nucleoli could be observed.

Next, we transfected FAM-labelled oligonucleotides htel22-L2a and -L2b into HeLa and U2OS and followed the process in 96-well plates using an Incucyte® S3 Live Cell imaging system by Sartorius via fluorescence microscopy. Qualitatively, all G-quadruplexes transfected with lipofectamine showed a successful penetration into the cytoplasm, where punctuate formations could be observed in many of the tested conditions, indicating a high local concentration of transfected DNA (SI, Supplementary Figs S84 and S85). The observed fluorescing aggregates could have various origins, ranging from intracellular agglomeration of the polyanionic oligonucleotides together with the polycationic transfecting reagent, endosomal compartmentalization or cytoplasmic aggregation with proteins [71–73]. Interestingly, when the modified G-quadruplexes were stabilized by coordination to Ni^2+^ prior to transfection, a strong nuclear staining could be observed, with only a minor formation of cytoplasmic punctuate objects with high fluorescence intensity (Fig. 6B). In contrast, no comparable nuclear enrichment was observed for the Cu^2+^ or Zn^2+^ complexes. In the case of Cu^2+^, this is likely attributable to quenching of the fluorescent signal, precluding reliable localization. For Zn^2+^, impaired nuclear uptake of the metalated construct may be responsible, though the underlying mechanism remains to be established. The question may arise whether one exclude partial unfolding under intracellular conditions and that aggregation could then result from DNA crosslinking via inter-strand metal-bipyridine complex formation. To this end, inspection of the MD structures indicates that the intact G-quadruplex assemblies rather sequester the bipyridine complexes within a pocket created by the loop region, rendering the invasion of a bipyridine from a neighbouring strand unlikely. Furthermore, *in vitro* CD spectroscopic data provided no evidence for unfolding/crosslinking upon metal complexation, and native ESI-MS experiments show that the metalated constructs exist as discrete monomeric species in solution without evidence of higher-order intermolecular assemblies (SI, Supplementary Figs S29–S41). Also, native polyacrylamide gel electrophoresis experiments at high concentration showed no evidence of high-order species forming (SI, Supplementary Fig. S89).

**Figure 7.**
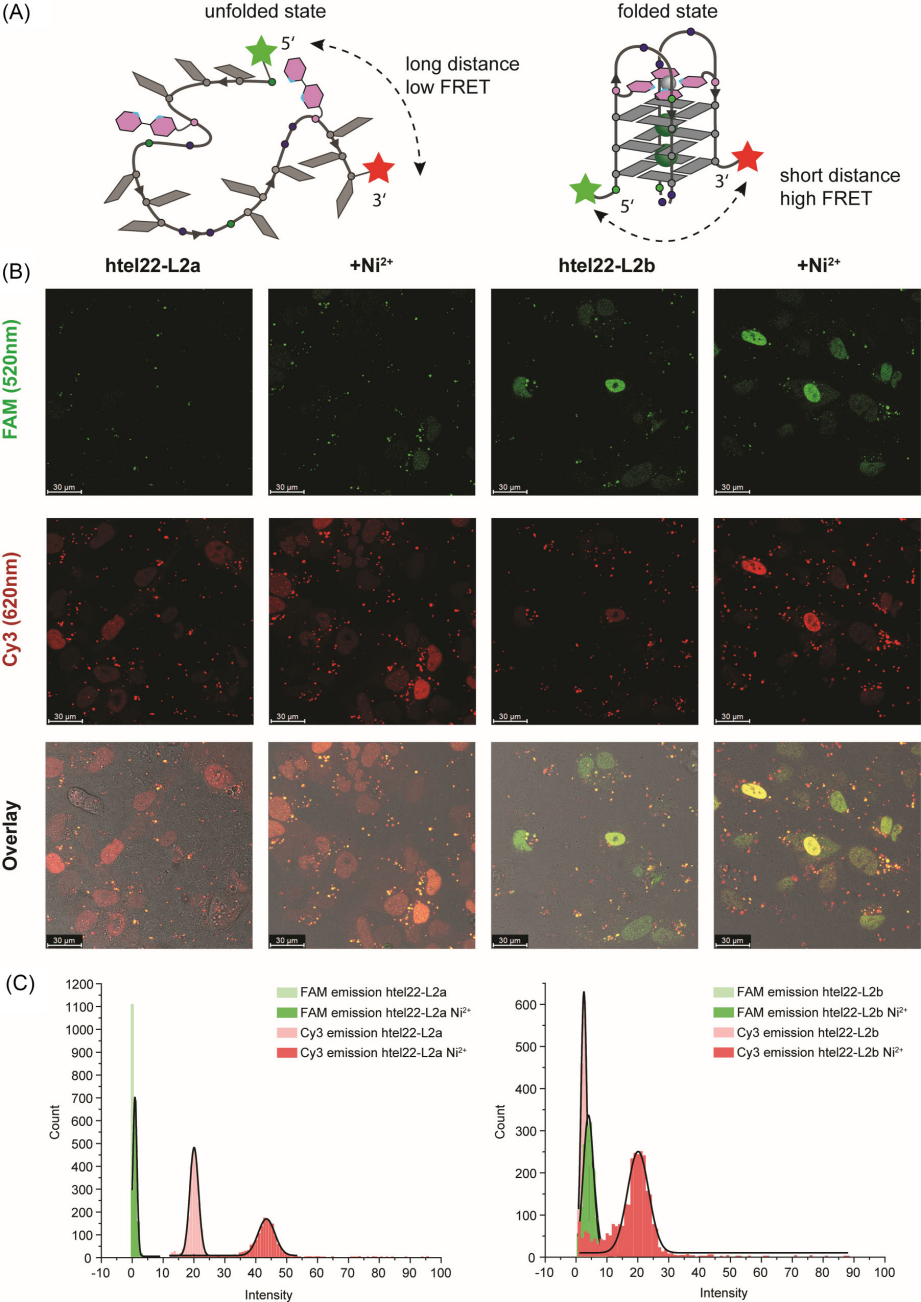
(**A**) Schematic representation of the FRET experiments. Low FRET efficiency is observed when the G-quadruplex is unfolded as donor and acceptor are further away. High FRET efficiency is observed when the G-quadruplex is folded as donor and acceptor are in close proximity. (**B**) Fluorescence microscopy of U2OS cells transfected with either htel22-L2a or htel22-L2b (without and with Ni^2+^). Images show filters applied for the individual fluorophores (FAM 520 nm, Cy3 595 nm) as well as overlay with the brightfield image (excitation wavelength corresponding to the excitation maximum of FAM donor, 495 nm). (**C**) Counts of fluorescence intensities of FAM and Cy3 fluorophores of htel22-L2a and -L2b (without and with Ni^2+^).

As shown in in previous sections, the Ni^2+^-bound quadruplexes show high stability under *in vitro* conditions. To investigate if this also holds true under cellular conditions, we transfected the double fluorescently labelled oligonucleotide strands of htel22-L2a and -L2b, bearing a FAM donor and a Cy3 acceptor, thus creating a FRET pair (Fig. 7A). When these modified oligonucleotides fold into a G-quadruplex, donor and acceptor fluorophores are in close proximity and hence show a high FRET efficiency (SI, Supplementary Fig. S87). We then transfected the modified htel22-L2a and -L2b sequences, pre-annealed in absence or presence of Ni^2+^, into U2OS cells (Fig. 7B). By plotting the counts of individual intensities (max intensity value: 255) from the nucleus, we created a histogram of the two emission wavelengths for the donor FAM and the acceptor Cy3 (see Fig. 7C). Overall htel22-L2a shows FAM donor emission with extremely low intensity (<5) both in presence and absence of Ni^2+^. The Cy3 acceptor emission intensity increased from a mean value of 20–44 when the G-quadruplex was stabilized with Ni^2+^, yet a lower maximum count was concomitant, which can be due to the quenching by the transition metal (see SI, Supplementary Fig. S86–S88). We conclude, however, that htel22-L2a remains partially folded even in the absence of Ni^2+^ and is further stabilized, as shown *in vitro*, by the metal, leading to a substantial FRET effect. The case is similar for htel22-L2b, where FAM donor fluorescence does not decrease in presence of Ni^2+^ but shows higher intensities (<10) than inhtel22-L2a. A slight increase in FAM donor emission can originate from the increased stabilization and better cellular transfection of the metal-complexed structures, as has been shown for the singly fluorescently labelled G-quadruplexes (see Fig. 6). Contrary to the previous strand, Cy3 acceptor fluorescence shows very low intensities in absence of Ni^2+^ (<5) for htel22-L2b, indicating that the G-quadruplex was either transfected improperly due to the lacking stabilization of Ni^2+^ or is unfolded in its absence. Similarly, we see that the metal-stabilized G-quadruplex shows an increased Cy3 donor fluorescence (with a mean value of 20). Again, partial quenching due to the involvement of the Ni^2+^ cations in the FRET process may explain a lower count.

## Conclusions

Taken together, we were successful in the implementation of a novel artificial ligandoside capable of forming transition metal complexes in DNA that not only affect the thermal stability of the resulting G-quadruplexes but can alter their respective topologies dynamically. We demonstrate the structural dependence of ligandoside placement and how positioning them within loops versus the G-core affects folding and stability. A clear metal-dependant topology switching from a hybrid to an antiparallel form was established. MD simulations support the experimental observation made for the different G-quadruplex topologies and give helpful structural insights. We further show that metal-triggered refolding of these structures withstands adverse molecular crowding conditions. Initial studies demonstrate that these functionalized G-quadruplexes can be transfected into live cells and that they most likely remain folded. Interestingly, coordination to Ni^2+^ improves transfection and nuclear localization of the modified G-quadruplexes. This paves the way for further applications of these biohybrid G-quadruplex constructs as potential interaction partners for proteins, acting as robust molecular decoys capable of binding to targets involved in certain diseases and serving as diagnostic probes. Our upcoming studies will focus on (i) the further development of minimal size/structure-impacting modifications to stick as close to wild-type loop and G-stack structures as possible, (ii) the screening for protein binding by libraries of such modified G4s, and (iii) the triggering biological responses by robust, metal-stabilized G-quadruplex constructs.

## Supplementary Material

gkag738_Supplemental_File

## Data Availability

Data for this article, including NMR and mass spectra, UV-Vis and CD results, and molecular dynamics simulation files, are available at the RESOLVdata repository (https://data.tu-dortmund.de/dataverse/resolv) at https://doi.org/10.17877/RESOLV-2025-MHT5G3K3.
