# Supplementary material for: Metal-triggered topology switching in bipyridine-modified DNA G-quadruplexes
Source: Nucleic Acids Res. 2026 Jul 28;54(14):gkag738. doi: 10.1093/nar/gkag738 (PMC13408043; doi:10.1093/nar/gkag738)
Supplement: gkag738_Supplemental_File [file gkag738_supplemental_file.pdf]

Metal-triggered Topology Switching in Bipyridine-modified DNA G-quadruplexes

Supporting Information

Table of Contents

1. Experimental Section 2
2. Oligonucleotide synthesis and purification 3
3. UV-Vis experiments details 4
4. CD Spectroscopy further details 5
5. Molecular Dynamics simulations details 5
6. Ligand Synthesis 7
7. Analytical HPLC of synthesized of Oligonucleotides 15
8. Mass Spectrometry 19
9. Native Mass spectrometry 25
10. Circular Dichroism (CD) spectroscopic analysis 30
11. Thermal Differential Spectroscopy Analysis 35
12. UV-Vis Melting Curve Analysis 40
13. Molecular Dynamics Simulation 46
14. PEG200 experiments 51
15. Cellular studies in HeLa and U2OS cells 52
16. Fluorescence Spectroscopy Analysis 54
17. References 56

1. Experimental Section

Solvents were purified from higher boiling impurities on the rotational evaporator (RE). The solvent toluene was commercially purchased in 99.8% purity from Fischer Scientific Company L.L.C. and degassed using the freeze-pump-thaw method. The solvent dioxane was purchased in 99% purity from Fischer Scientific Company L.L.C. and degassed using the freeze-pump-thaw method. The solvents ethanol (EtOH), methanol (MeOH), dicholormethane (DCM) and ethylacetate (EtOAc) were used as technical and high performance liquid chromatography (HPLC) grade solvents.

Reactions involving substrates or reagents sensitive towards ambient air and/or hydrolysis were carried out in under vacuum heated glassware using the Schlenk technique under argon. Syringes and cannulas were washed with inert gas before usage.

Yields were determined via gravimetrical analysis using an analytical scale (variance: d = 0.1 mg). The yield was given as a weight percentage, relative to the amount of used substrate.

Reagents and substrates were either readily available from the groups chemical reservoir or were commercially purchased and used without further purification steps.

^{1}H-NMR-Spectra were obtained using Bruker *AV 400*, Bruker *AV 500 Avance NEO* / Agilent *DD2* und Bruker *AV 600* spectrometers manufactured by Bruker Physics and were carried out in deuterated chloroform ($CDCl_3$), deuterated acetonitrile (CD_3CN), deuterated dimethyl sulfoxide (DMSO-d_6), deuterated tetrahydrofuran (THF-d_8), deuterated methanol (MeOD), deuterated acetone (Aceton-d_6) and deuterated dimethylformamide (DMF-d_7). The chemical shifts δ were determined relative to a standard (trimethylsilane, δ = 0 ppm) in ppm. All ^{1}H-NMR-spectra were calibrated on the residual proton signal of the respective deuterated solvents ($CDCl_3$: δ = 7.26 ppm, CD_3CN: δ = 1.93 ppm, DMSO-d_6 = 2.49 ppm). The coupling patterns were denoted with the following terms: s (singulet), d (doublet), t (triplet), m (multiplet).

^{13}C-NMR-Spectra were obtained using Bruker *AV 400*, Bruker *AV 500 Avance NEO* / Agilent *DD2* und Bruker *AV 600* spectrometers manufactured by Bruker Physics and were carried out in deuterated chloroform ($CDCl_3$), deuterated acetonitrile (CD_3CN) and deuterated dimethyl sulfoxide (DMSO-d_6). The chemical shifts δ were determined relative to a standard (trimethylsilane, δ = 77.0 ppm) in ppm. All ^{1}H-NMR-spectra were calibrated on the residual

proton signal of the respective deuterated solvents ($CDCl_3$: δ = 77.16 ppm, CD_3CN: δ = 1.3, 117.7 ppm, DMSO-d_6 = 39.7 ppm).

2. Oligonucleotide synthesis and purification

Table S1. Reagents for DNA synthesis.

Name	Reagent	Composition
DCA	Detritylation	3% (v/v) dichloroacetic acid in anhydrous dichloromethane
ACT	Activator	0.3 M 5-(benzylthio)-1H-tetrazole in anhydrous acetonitrile
Cap A	Capping A	10% (v/v) N-methyl imidazole in anhydrous tetrahydrofurane
Cap B	Capping B	2,6-lutidine / acetic anhydride / anhydrous tetrahydrofurane 1:1:8 (v/v/v)
OXI	oxidizer	0.02 M iodine in tetrahydrofurane / pyridine / water 7:2:1 (v/v/v)
ACN	solvent	anhydrous acetonitrile

All oligonucleotides were synthesized on a K&A Laborgeraete GbR H-8 synthesizer on a 1 µmol scale using the standard phosphoramidite methods on controller pore glass (CPG) and following previously published procedures for synthesis. Standard phosphoramidites (DMT-dT-CEP, DMT-dG(iBu)-CEP, DMT-dA(bz)-CEP, DMT-dC(Ac)-CEP) were used and the cartridges with CPG solid supports (1000 Å, 25-35 µmol/g, DMT-dT-CPG and DMT-dG(iBu)-CPG) were manually packed. The oligonucleotide synthesis followed the built-in methods of the DNA synthesizer and was slightly modified. First, the cartridges were treated three times with DCA to deprotect the 5'-OH groups. Second, coupling was achieved by mixing the respective phosphoramidite building block (0.1 M in ACN) with ACT (1:1, v/v). The coupling time was ~0.5 min for standard phosphoramidites and ~3.5 min for the ligand-modified phosphoramidite. Third, the cartridge was treated with a 1:1 (v/v) mixture of Cap A and Cap B to acetylate unreacted 5'-OH groups, which was followed, by the oxidation with OXI. Here, an additional washing step with CAN was introduced compared to the standard routine. After each individual step of the cycle, the cartridge was washed with ACN followed by a drying step with argon. The described cycle was repeated for every incorporated nucleotide. After DNA synthesis, the solid supports were removed from the cartridges and treated with concentrated aqueous NH_3 solution at 55 °C overnight for cleavage and deprotection. The supernatant solution was filtered (VWR Centrifugal filters) and the solid support was washed with 100 µL water. NH_3 was removed from the filtrate under reduced pressure using a H. Saur Laborbedarf S-Concentrator BA-VC-300H vacuum concentrator and the volume of the solution was reduced to ~300 µL. Purification of the oligonucleotides was performed with reversed-phase HPLC on an Agilent Technologies 1260 Infinity II HPLC system equipped with an autosampler,

column oven, DAD detector and a Macherey-Nagel VP 250/10 Nucleodur 100-5 C18ec column (oven temperature: 60 °C, flow rate: 2.5 mL/min, solvent A: 50 mM TEAA pH 7, solvent B: 70:30 MeCN/50 mM TEAA pH 7, gradient: from 100% solvent A to 20% solvent A and 80% solvent B in 30 min). To remove ACN from the sample, the volume of the solution was again reduced to ~300 µL in the vacuum concentrator and then the sample was diluted with100 mM TEAA pH 7 to a volume of 2 mL. Subsequently, the cleavage of the 5′-OH DMT protecting groups (with 2% TFA) and desalting were accomplished using Waters Sep-Pak C18 cartridges. Desalted oligonucleotides were lyophilized using a Christ Alpha 2-4 LSC basic lyophilisation device and stored either as a solid or as 0.5–2.5 mM stock solutions in water (MQ water, pH 7.2) at 4 °C. The concentrations of all oligonucleotide stock solutions were determined via the absorbance at 260 nm at 25 °C with a Thermo Scientific Nanodrop One instrument and using revised extinction coefficients for the nucleosides.

3. UV-Vis experiments details

Both UV-VIS spectra and thermal denaturation profiles (melting curves) were recorded on a Jasco V-750 UV-Visible Spectrophotometer equipped with a PAC-743 6-cell thermostat for temperature control. The temperature was measured in the measurement cell in a water-filled cuvette. Quartz glass cuvettes (Hellma Analytics 114-QS, 1 cm path length) were used. In order to avoid condensation of water on the cuvette surface or cell window at low temperatures, a constant flow of air was pumped through the measurement cell. Evaporation of water at high temperatures and resulting changes in the absorption behaviour were minimized by a thin layer of silicon oil placed onto the sample and by tightly stoppering the cuvette. UV-VIS spectra were recorded from 350 to 220 nm with a scan rate of 200 nm/min both before (4 °C) and after thermal denaturation (95 °C). The data interval was set to 1 nm, bandwidth to 2.0 nm and the response time to 0.96 sec. All UV-VIS spectra were background corrected (cuvette, buffer and electrolyte) and zeroed using the absorption at 350 nm. To obtain the thermal difference spectra (TDS), the spectrum before denaturation (at 4 °C) was subtracted from the one after denaturation (at 95 °C). A negative band (hypochromic shift) at 295±2 nm and positive bands at 243±2 nm and 273±2 nm (hyperchromic shift) indicated G-quadruplex formation. For the thermal denaturation profiles (melting curves), absorption of the samples at 295 nm was recorded in a 0.5 °C interval with a temperature gradient set to 0.5 °C/min, which corresponds to ~0.174 °C/min including the measurement time. Data points were recorded from 4 °C to 90 °C. Melting curves were background corrected using the absorption at 350 nm and normalised from 0.0 to 1.0 absorption and a first order derivative calculated to determine the minimum which corresponded to the melting point (T_m).

Determination of binding constants of the individual metals (M^{2+} = Cu, Ni, Zn) with the respective G-quadruplexes was performed via titration experiments followed by UV-Vis spectroscopy. G-quadruplex samples were prepared identical to standard UV-Vis thermal denaturation studies. A concentrated solution of the metal salts was prepared (1 mM) and added in 0.1 eq steps until a total of 2.5 eq was reached. After each addition the sample was mixed thoroughly and incubated at r.t. for 15min to secure full complexation and subsequently a UV-Vis spectrum recorded. The absorbance of the metal-complex at 315 nm was then plotted against the increasing equivalents of metal cations to ensure that a saturation point had been reached. The binding constant was then calculated using the Benesi-Hildebrandt method by plotting the $1/\Delta A$ against $1/c(M^{2+})$ with $c(M^{2+})$ being the concentration of the metal cations in solution for each step. A linear fit through the data was performed and the slope determined from which the binding (association) constant could be extracted.

4. CD Spectroscopy further details

For the kinetic measurements by CD spectroscopy, the samples were prepared in the same way as for the UV-VIS-based thermal denaturation studies. The CD signal at 260 nm was chosen as this was indicative of the formation of an antiparallel G-quadruplex topology formed from the hybrid topology. The sample was kept at the desired temperature by the temperature control with an in-sample thermometer. At t = 0 s, 4.4 µL of 1 mM metal salt solution was added to the DNA sample and quickly mixed by pipetting. The measurement intervals were 15 s over a period of up to 20000 s. The recorded CD intensities were then plotted over time and fitted with an exponential decay curve which was used to calculate the concentration of the G-quadruplex without any metal (educt). To assess the order of this reaction we plotted the concentration of the G-quadruplex without any metal bound on a logarithmic scale over the recorded time. This was repeated for each metal cation (Cu^{2+}, Ni^{2+}, Zn^{2+}) and over four temperatures (284 K, 289 K, 298 K, 306 K). Out of the corresponding kinetic rate constants an Eyring plot was created to extract the enthalpy ($\Delta H^{\ddagger}$) and entropy ($\Delta S^{\ddagger}$) of activation as well as the activation energy at each temperature (E_a or $\Delta G^{\ddagger}$).

5. Molecular Dynamics simulations details

Force field parameters and RESP charges for the artificial nucleotide **BiPy-L** and its Cu^{2+} complex were generated in the identical manner as previously described.[1] The uncoordinated ligand nucleoside was split into fragments, according to the original AMBER force field implementation (dimethylphosphate)[2] and capped with 5' and 3' OH groups. For the linkage between the pyridine and the glycol backbone $-CH_3$ and $-OCH_3$ were used as

caps, respectively. The standard $–CH_3$ and –OH approach resulted in less optimal RESP charge fits. All molecules were geometry optimized at the HF/6-31G(d)level using Gaussian ’09. In case of the M^{2+} complex, metal bound to two of 4-(methoxymethyl)bipyridine units was used and was geometry optimized at the DFT B3LYPlevel of theory with the 6-31G(d) basis set. For all bipyridine containing molecules two different conformations were found with similar energies. Partial point charges were then derived by RESP charge fitting using the REDServer-Development.[3–5] Inter-molecular and intra-molecular charge constraints were used to maintain the correct total charge of the ligand. For the metal complex, the $–CH_3$ cap of the pyridine units was constrained to the same values obtained earlier for the uncoordinated ligand, to avoid problems arising from the different basis sets used. For the metal ion, a van-der-Waals radius of 1.7 Å was used. In all cases, the constrained charges were similar to the unconstrained values.

For the ligand alone, force field parameters missing in the AMBER force field ff99 bsc1 were generated with parmchk2 from the AmberTools, based on analogy to existing force field entries (see Table 4). Parameters for the copper complex were derived using the VFFDT program, which is based on the Seminario method, using a geometry-optimized model (B3LYP/6-311+G(d,p) (CHNO), def2-TZVPD (Cu) level of theory). To obtain a square-planar coordination environment for the Cu^{2+} ion, adjacent ligands were named differently, in order to avoid ambiguity in the parameterization. Improper dihedrals were used to maintain coplanarity between the pyridine plane and the pyridine – metal plane. The respective force constants were estimated based on literature values.

First, initial structures for the G-quadruplex (**htel22-L2a** and **htel22-L2b**) were constructed by using the solid-state or solution-sate structures of the respective parental G-quadruplex and the topologies observed by CD (PDB entries: 143D, 2JPZ, 2HY9).[6–8] All manipulations were carried out in UCSF Chimera.[9] Na^+ ions were replaced by K^+ ions. Redundant ions and redundant nucleotides were deleted, the geometry-optimized ligands or the respective Cu^{2+}-complex was inserted manually. The respective models obtained above were put in a periodic rhombic dodecahedron box (cut off 1.5 nm) and energy minimized 2000 steps of steepest descent (600 kJ/mol nm tolerance) in vacuum. PME and van-der-Waals cut off of 1.3 nm were used. The system was solvated with TIP3P water molecules and the negative charge of the system was neutralized with the corresponding amount of randomly positioned K^+ ions. An additional 100 mmol/L KCl was added to simulate the ionic strength of the experiments. The system was then energy minimized in two steps, first 500 steps of steepest descent (500 kJ/mol nm tolerance) and then 3000 steps of conjugate gradient minimization (300 kJ/mol nm tolerance). The non-bonded Lennard-Jones cut off was set to 1.3 nm, the non-bonded pair list updated every 50 steps. For the coulombic interactions, Particle-mesh Ewald summation (PME) was used.[10] Next, the system was equilibrated with positional constraints on the

model's heavy-atoms 100 ps in a first round (NVT ensemble, constraints 1000 kJ/mol Å^2, time step 2 fs; Temperature coupling modified Berendsen, 298 K); second round 100 ps with additional pressure coupling (isotropic, Berendsen, 1 bar, time constant for coupling 0.1 ps, compressibility 4.5 10−5) and a third round 100 ps but with lower constraints(100 kJ/mol Å^2, Nose-Hoover temperature coupling, 2 ps coupling, Parinello-Rahman isotropic pressure coupling, 2 ps coupling time). The equilibration phase was finished with 200 ps of an unconstraint DNA MD run (coupling times increased to 4 ps). A 100 ns MD production run was then performed. Coordinates were written every 10 ps, resulting in 10001 frames per trajectory. Trajectories were centered, aligned and fitted to the first frame using the built-in Gromacs tools and then analysed and visualized with UCSF Chimera. The depicted structures are representatives of the MD trajectories. For the RMSD plots, the first frame of the trajectory was used as the reference.

6. Ligand Synthesis

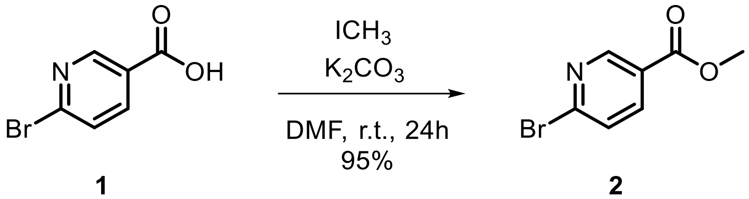

1 **2**

6-Bromo-nicotinic acid (**1**) (1 g, 4.95 mmol, 1 eq.) was dissolved in DMF (30 mL), methyl iodide (703.65 mg, 4.96 mmol,1.1 eq) and K_2CO_3 (1.03 g, 7.43 mmol, 1.5 eq.) were added, followed by stirring the solution overnight at room temperature (r.t.). The solution was diluted with water (100 mL), and the product was extracted with EtOAc (3 × 50 mL). The combined organic phases were washed with water (2 × 50 mL) and brine (2 × 50 mL), and the mixture was dried over $MgSO_4$. The solvent was evaporated under reduced pressure, and the substance was purified by column chromatography (SiO_2, pentane → pentane/EtOAc 10:1). Methyl-6-bromoisonicotinate (3) (1016.3 mg, 4.7 mmol, 95%) was obtained as a yellow oil.

^{1}H NMR (600 MHz, $CDCl_3$) δ (ppm) = 8.95 (dd, J = 2.4, 0.7 Hz, 1H, a), 8.12 (dd, J =8.3, 2.4 Hz, 1H, b), 7.58 (dd, J = 8.3, 0.8 Hz, 1H, c), 3.95 (s, 3H, d).

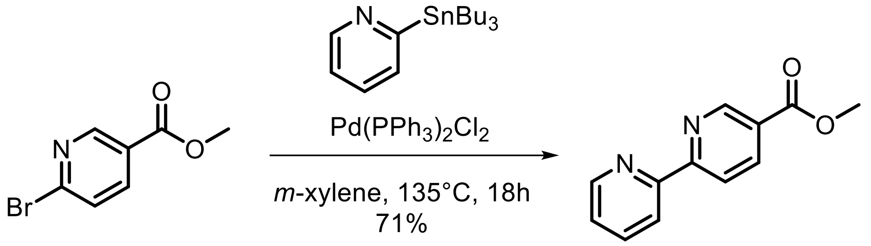

2 **5**

The reaction was carried out in degassed xylene (20 mL), which was subjected to the freeze-pump-thaw (FPT) method. Methyl 6-bromoisonicotinate (**2**) (3.0 g, 13.89 mmol, 1 eq.) was dissolved in the solvent. Subsequently, 2-(tributylstannyl)pyridine (5.11 g, 13.89 mmol, 1.1 eq.) and bis(triphenylphosphine)dichloropalladium(II) (487.36 mg, 694.34 µmol, 0.05 eq.) were added to the solution. The mixture was heated at 135 °C for 18 hours. The resulting suspension was extracted with EtOAc, washed three times with aqueous potassium fluoride solution (1 M), and once with brine. The precipitated Bu_3SnF was removed by filtration through a KF/Celite mixture. The combined organic phases were dried over $MgSO_4$ and purified by column chromatography (SiO_2, pentane → pentane/EtOAc 10:1 → pentane/EtOAc 5:1). 5-(Methoxycarbonyl)-2,2'-bipyridine (5) (2.1 g, 9.72 mmol, 71%) was obtained as orange-brown oil.

^{1}H NMR (500 MHz, $CDCl_3$) δ (ppm) = 9.29 (s, 1H, a), 8.75 (d, J = 2.9 Hz, 1H, b), 8.59 (d, J = 8.2 Hz, 1H, c), 8.53 (d, J = 8.1 Hz, 1H, d), 8.44 (dd, J = 8.4, 2.1 Hz, 1H, e), 7.93 (td, J = 7.7, 1.8 Hz, 1H, f), 7.43 (dd, J = 7.9, 4.6 Hz, 1H, g), 3.98 (s, 3H, h).

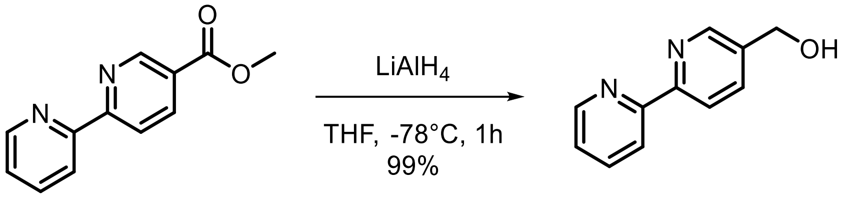

5 **7**

A solution of 5-(methoxycarbonyl)-2,2'-bipyridine (5) (420 mg, 1.96 mmol, 1 eq.) in anhydrous THF (50 mL) was cooled to –78 °C. A 1 M solution of lithium aluminium hydride in THF (2.16 mL, 2.16 mmol) was added dropwise. The reaction mixture was warmed to –20 °C and stirred for 0.5 h until a homogeneous solution was formed. Subsequently, the mixture was cooled again to –78 °C and quenched dropwise with 30 mL of 10% aqueous THF. After reaching room temperature, the reaction mixture was stirred with dry Celite for 15 minutes, filtered, and the

Celite was washed with approximately 100 mL of THF. The solvent was removed under reduced pressure after drying with $MgSO_4$. [2,2'-Bipyridin]-5-ylmethanol (**7**) (365.08 mg, 1.96 mmol, 100%) was obtained as a dark orange oil which was used directly in the subsequent reaction without further purification.

^{1}H NMR (500 MHz, $CDCl_3$) δ (ppm) = 8.73 (d, J = 4.6 Hz, 2H, a/a'), 8.48 (t, J = 9.2Hz, 2H, b/b'), 7.95 (d, J = 7.0 Hz, 1H, c), 7.91 (t, J = 7.6 Hz, 1H, d), 7.39 (t, J = 6.0 Hz,1H, e), 4.82 (s, 2H, f).

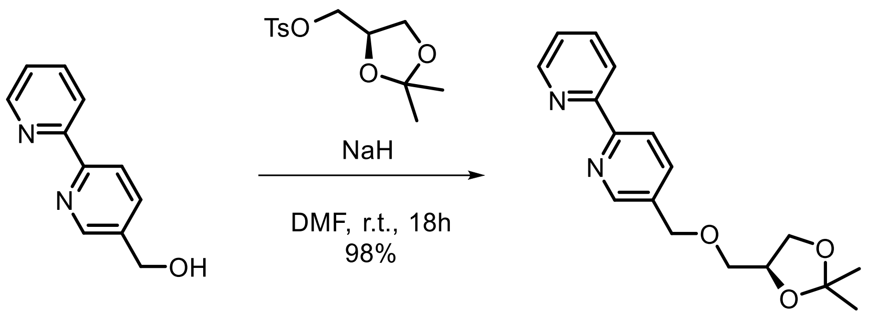

7 **9**

Sodium hydride (60% suspension in mineral oil, 136.41 mg, 5.68 mmol, 2.9 eq.) was washed three times with THF (10 mL) and dried under vacuum. Anhydrous DMF (10 mL) was then added, and the mixture was cooled to 0 °C. A solution of [2,2'-bipyridin]-5-ylmethanol (**7**) (365 mg, 1.96 mmol, 1 eq.) in anhydrous DMF was added dropwise to the suspension, followed by stirring for 30 minutes at 0 °C. Subsequently, (R)-(2,2-dimethyl-1,3-dioxolan-4-yl)methyl 4-methylbenzenesulfonate (645 mg, 781 µL, 2.25 mmol, 1.15 eq.) was added in portions (0.2 mL every 5 minutes). The reaction mixture was stirred at 0 °C for an additional hour and then allowed to stir at room temperature overnight. The reaction was quenched by the careful addition of water (30–40 mL), and the product was extracted with EtOAc (3 × 50 mL). The combined organic phases were washed with water (4 × 80 mL) and brine (4 × 80 mL), dried over $MgSO_4$, and concentrated under reduced pressure and purified by column chromatography (SiO_2, pentane → pentane/EtOAc 10:1 → pentane/EtOAc 1:1). 5-(((2,2-dimethyl-1,3-dioxolan-4-yl)methoxy)methyl)-2,2'-bipyridine (9) (575.68 mg, 1.92 mmol, 98%) was obtained as orange oil.

^{1}H NMR (500 MHz, $CDCl_3$) δ (ppm) = 8.61 (ddd, *J* = 4.7, 1.9, 1.0 Hz, 1H, a), 8.57 (d, *J* = 3.1 Hz, 1H, b), 8.32 (d, *J* = 7.9 Hz, 1H, c), 7.78 – 7.73 (m, 2H, d/d'), 7.27 – 7.23 (m, 2H, e/e'), 4.64 – 4.55 (m, 2H, f), 4.29 – 4.18 (m, 1H, g), 3.99 (ddd, *J* = 12.7, 8.3, 6.5 Hz, 1H, h), 3.68 (ddd, *J* = 14.6, 8.2, 6.4 Hz, 1H, i), 3.57 – 3.45 (m, 2H, j), 1.36 (s, 6H, k/k').

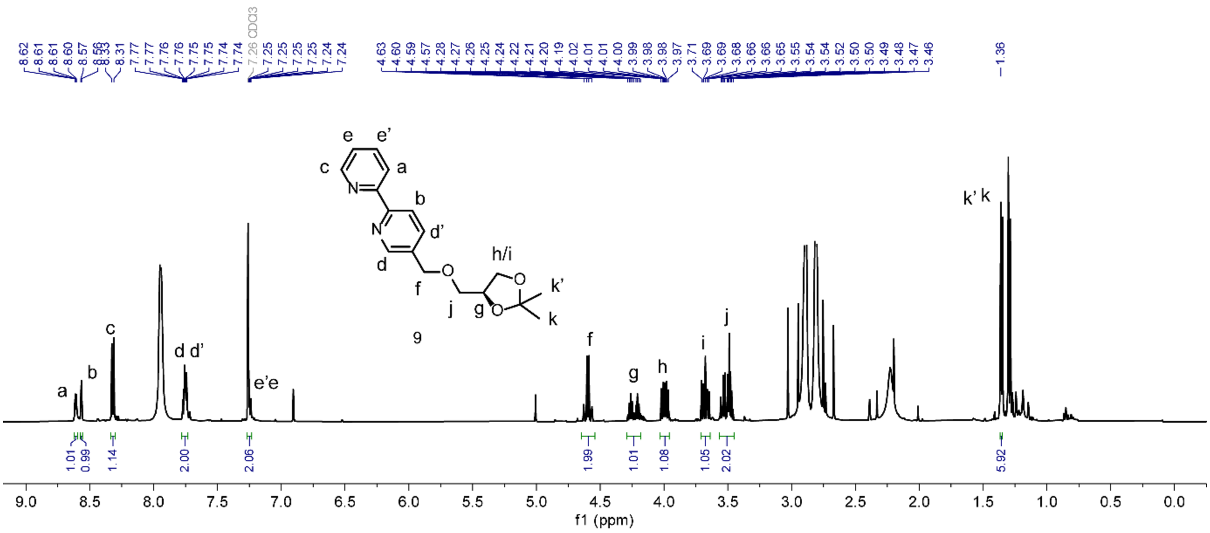

Figure S1. ^{1}H-NMR spectrum of 5-(((2,2-dimethyl-1,3-dioxolan-4-yl)methoxy)methyl)-2,2'-bipyridine (9) in $CDCl_3$. 500MHz, 298K.

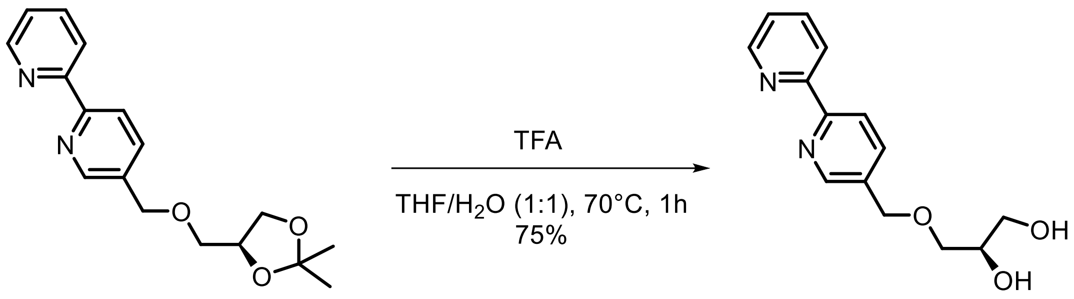

9 **11**

5-(((2,2-Dimethyl-1,3-dioxolan-4-yl)methoxy)methyl)-2,2'-bipyridine (**9**) (517 mg, 1.72 mmol, 1 eq.) was dissolved in a mixture of THF and water (4 mL, 1:1 v/v). Trifluoroacetic acid (215.9 mg, 145.0 µL, 1.89 mmol, 1.1 eq.) was added dropwise to the solution. The reaction mixture was heated to 70 °C (reflux) for 1 hour, followed by stirring overnight at room temperature. The acidic suspension was neutralized with aqueous ammonia (25% v/v), and the solvents were removed under reduced pressure. The residue was purified by column chromatography (SiO_2, chloroform/methanol 10:1), yielding 3-([2,2'-bipyridin]-5-ylmethoxy)propane-1,2-diol (11) (196.18 mg, 0.75 mmol, 75%) as yellow oil.

^{1}**H NMR** (600 MHz, $CDCl_3$) δ (ppm) = 8.72 (ddd, *J* = 4.9, 1.8, 0.9 Hz, 1H, a), 8.69 (d, *J* = 1.6 Hz, 1H, b), 8.47 (dd, *J* = 8.1, 6.1 Hz, 2H, c/c'), 7.92 – 7.82 (m, 2H, d/d'), 7.38 (ddd, *J* = 7.5, 4.9, 1.2 Hz, 1H, e), 4.66 (s, 2H, f), 3.98 – 3.92 (m, 1H, g), 3.75 (dd, *J* = 11.4, 3.9 Hz, 1H, h/i), 3.69 – 3.59 (m, 3H, i'), 3.48 (s, OH, j), 2.63 (s, OH, k).

^{13}C NMR (125 MHz, CD_2Cl_2) δ (ppm) = 165.30 (C5) , 150.04 (C1, C8), 139.17 (C3, C7), 124.83 (C6), 123.74 (C5), 122.33 (C2), 121.27 (C4), 120.63 (C4), 72.89 (C9), 71.85 (C10), 70.53 (C11), 52.93 (C12).

HR-ESI-MS (positive mode, acetonitrile): *m/z* calc. for 261.1239 $[M+H]^+$, found 261.1211.

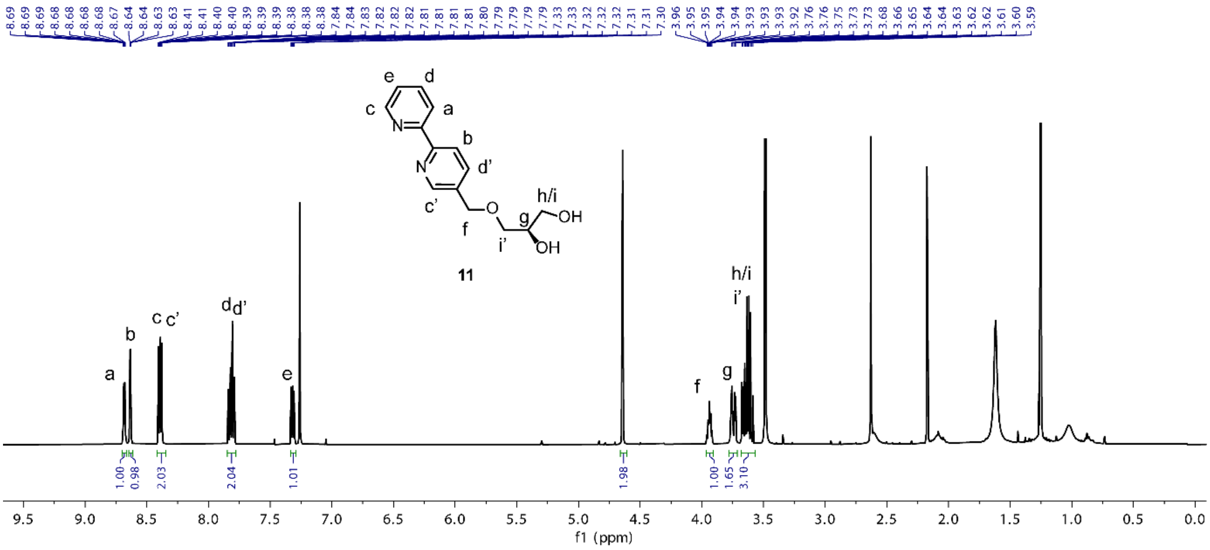

Figure S2. ^{1}H-NMR spectrum of 3-([2,2'-bipyridin]-5-ylmethoxy)propane-1,2-diol (11) in $CDCl_3$. 500MHz, 298K.

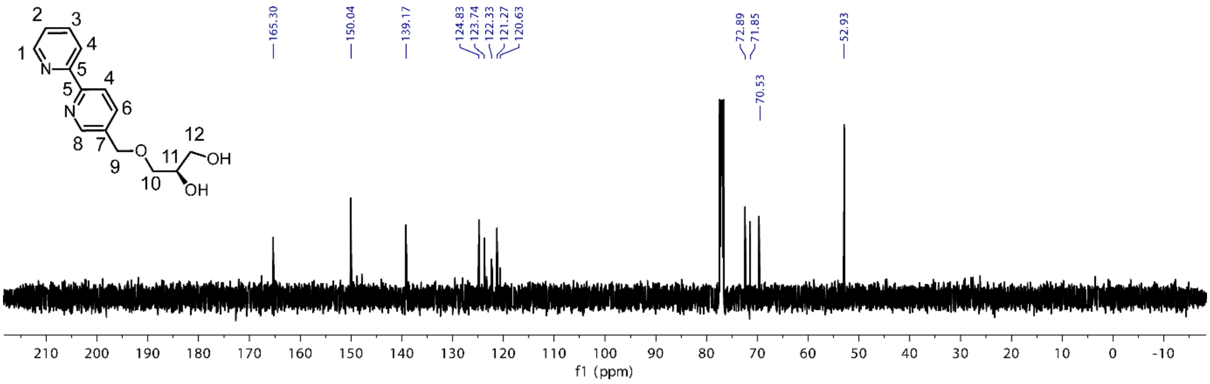

Figure S3. ^{13}C-NMR spectrum of 3-([2,2'-bipyridin]-5-ylmethoxy)propane-1,2-diol (11) in CD_2Cl_2. 500MHz, 298K.

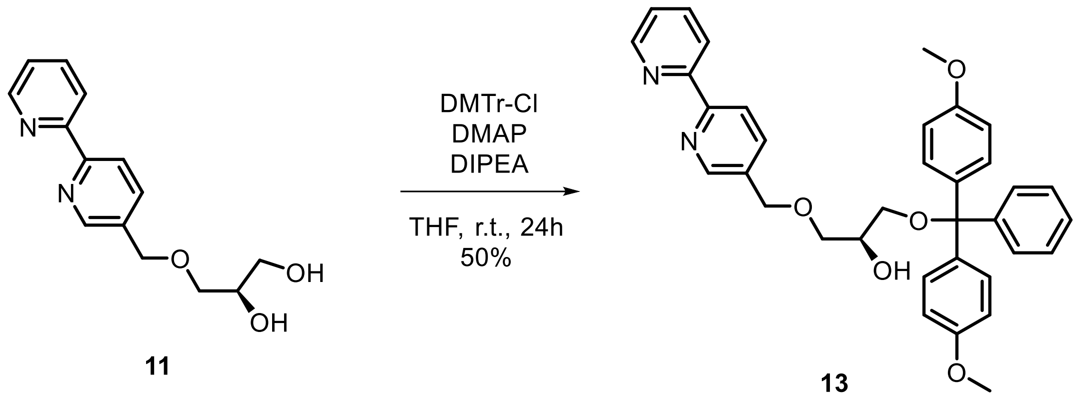

Under an inert atmosphere, 3-([2,2'-bipyridin]-5-ylmethoxy)propane-1,2-diol (**11**) (196.18 mg, 0.75 mmol, 1 eq.) was dissolved in anhydrous THF (3 mL). 4-Dimethylaminopyridine (5.5 mg, 45.2 µmol, 0.06 eq.) was added, followed by the slow addition of 4,4′-dimethoxytrityl chloride (280.6 mg, 828.3 µmol, 1.10 eq.) as a solid in portions. Diisopropylethylamine (256.1 µL, 1.51 mmol, 2.00 eq.) was then added to the mixture. The reaction was stirred overnight at room temperature. The suspension was diluted with methanol (1 mL) and ethyl acetate (50 mL), and the combined organic phases were washed with saturated aqueous sodium hydrogen carbonate solution (3 × 25 mL), water (3 × 25 mL), and brine (3 × 25 mL). The organic phase was dried over magnesium sulfate, and the solvents were removed under reduced pressure. Purification by column chromatography (SiO_2, ethyl acetate, 1% v/v triethylamine) yielded (S)-1-([2,2'-bipyridin]-5-ylmethoxy)-3-(bis(4-methoxyphenyl)(phenyl)methoxy)propan-2-ol (**13**) (210 mg, 373 µmol, 49%) as a white-gray solid.

^{1}H NMR (500 MHz, CD_2Cl_2) δ (ppm) = 8.61 (ddd, J = 4.7, 1.9, 1.0 Hz, 1H, a), 8.57 (d,J = 3.1 Hz, 1H, b), 8.32 (d, J = 7.9 Hz, 1H, c), 7.78 – 7.73 (m, 2H, d/d'), 7.27 – 7.23 (m,2H, e/e'), 4.64 – 4.55 (m, 2H, f), 4.29 – 4.18 (m, 1H, g), 3.99 (ddd, J = 12.7, 8.3, 6.5 Hz,1H, h), 3.68 (ddd, J = 14.6, 8.2, 6.4 Hz, 1H, i), 3.57 – 3.45 (m, 2H, j), 1.36 (s, 6H, k/k').

^{13}C NMR (125 MHz, CD_2Cl_2) δ (ppm) = 158.62 (C5), 149.22 (C1), 149.10 (C8), 145.04 (DMT-C_{ar}), 136.83 (C3), 135.95 (C7), 130.01 (DMT-C_{ar}), 129.99 (DMT-C_{ar}), 128.04 (C6), 127.84, 127.79 (DMT-C_{ar}), 126.74 (DMT-C_{ar}), 123.75 (C2), 121.81 (C4), 120.92 (C4), 113.10 (DMT-C_{ar}), 113.04 (DMT-C_{ar}), 86.06 (DMT-C_{ar}), 72.33 (C9), 71.82 (C10), 69.92 (C11), 64.44 (C12), 55.17 (DMT-OCH_3).

HR-ESI-MS (positive mode, acetonitrile): *m/z* calc. for 563.2546 $[M+H]^+$, found 563.2531.

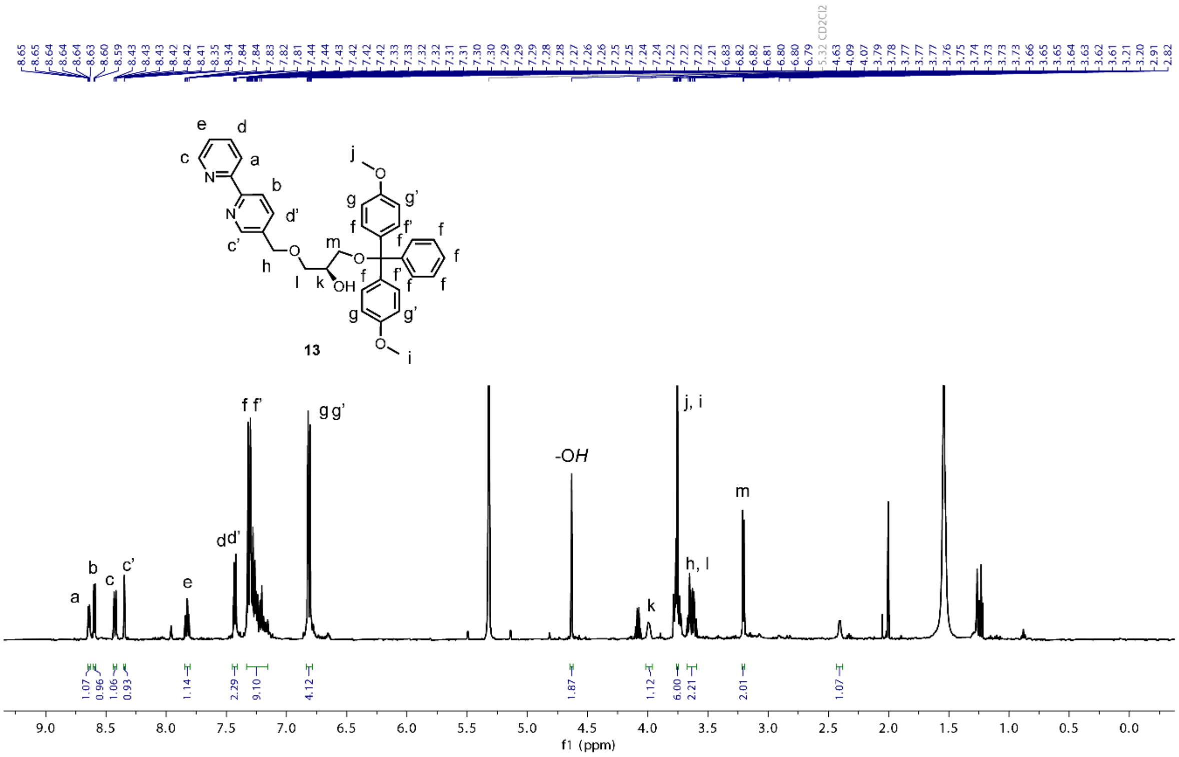

Figure S4. ^{1}H-NMR spectrum of (S)-1-([2,2'-bipyridin]-5-ylmethoxy)-3-(bis(4-methoxyphenyl)(phenyl)methoxy)propan-2-ol (**13**) in CD_2Cl_2. 500 MHz, 298K.

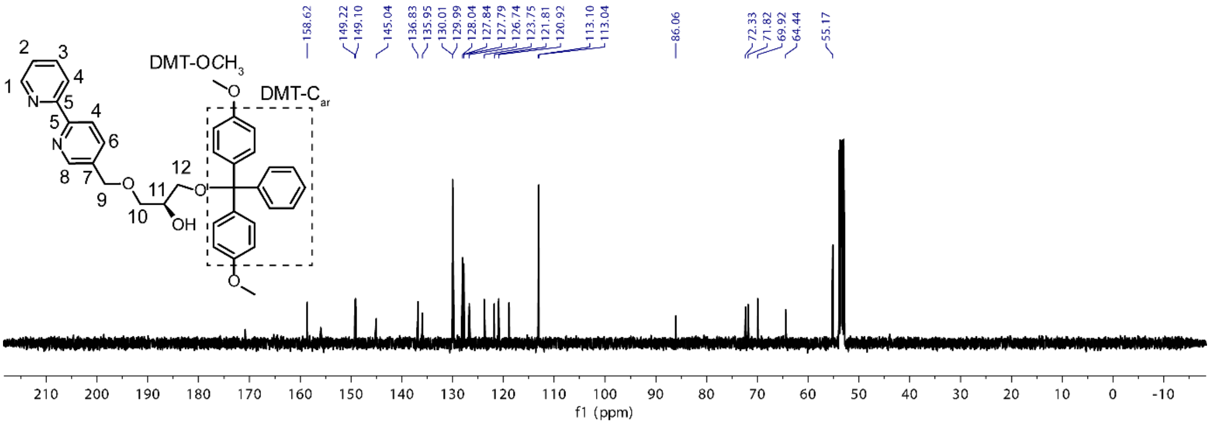

Figure S5. ^{13}C-NMR spectrum of (S)-1-([2,2'-bipyridin]-5-ylmethoxy)-3-(bis(4-methoxyphenyl)(phenyl)methoxy)propan-2-ol (**13**) in CD_2Cl_2. 125 MHz, 298K.

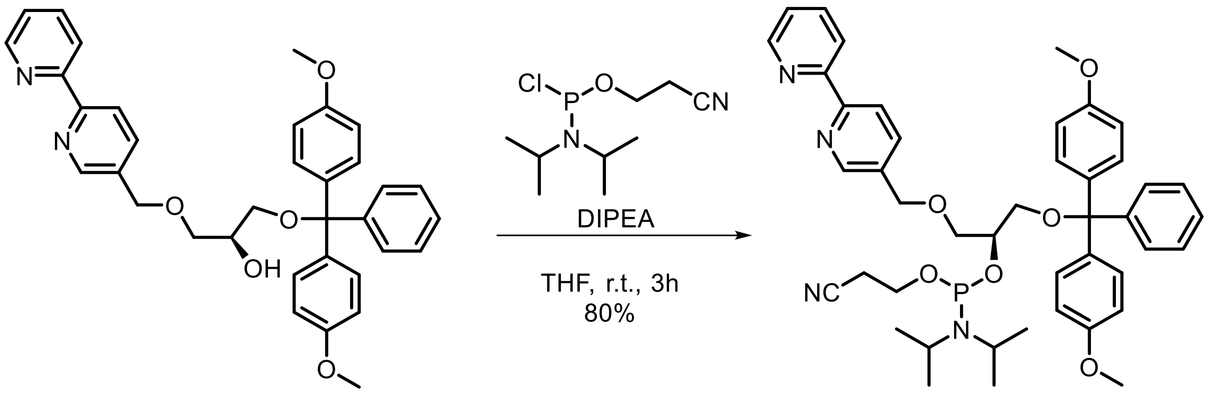

13 **15**

Compound **13** was co-evaporated with anhydrous THF (2 × 1 mL) under an inert atmosphere and then dissolved in anhydrous THF (6 mL). DIPEA (4.00 eq.) was added dropwise, followed by the dropwise addition of 2-cyanoethyl-N,N-diisopropylchlorophosphoramidite (CEDIP-Cl) (1.10 eq.). The resulting solution was stirred for 2–3 hours at room temperature, during which a white precipitate formed. The reaction mixture was filtered through a syringe filter to remove the precipitated diisopropylethylammonium chloride. The solvent was removed under reduced pressure, yielding the final compound **15** as yellow oil. The crude product was used without further purification, either directly or after storage at –25°C and was utilized within several days for automated DNA synthesis.

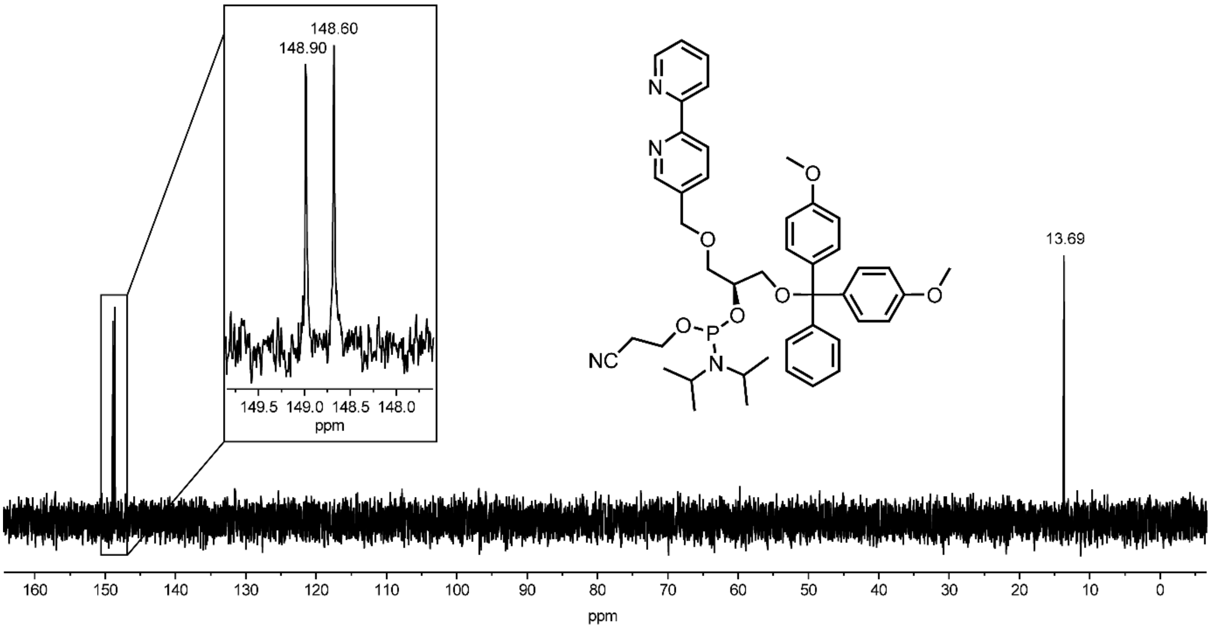

Figure S6. 31P NMR spectrum of the bipyridine phosphoramidite in CD_3CN. 500 MHz, 298K.

^{31}P NMR (202 MHz, CD_3CN) δ (ppm) = 148.91, 148.60, 13.76 (hydrolised/oxidised CEDIP-Cl reagent).

7. Analytical HPLC of synthesized of Oligonucleotides

Analytical reverse-phase HPLC (RP-HPLC) was performed to assess the purity of synthesized and purified oligonucleotides. Samples (10 µL, ~5 µM DNA concentration) were prepared in 20 mM triethylammonium acetate (TEAA) buffer (pH 7.0). Analyses were conducted on an Agilent Technologies 1260 Infinity II system equipped with an autosampler, column oven, and DAD detector, using a Macherey-Nagel NUCLEODUR 100-5 C18ec column (250 × 4.6 mm, 5 µm; oven temperature: 60 °C; flow rate: 0.75 mL/min). The mobile phase consisted of solvent A (50 mM TEAA, pH 7.0) and solvent B (70:30 acetonitrile/50 mM TEAA, pH 7.0), with a defined linear gradient program. Oligonucleotide purity was quantified as the percentage of the target peak area relative to the total integrated peak area in the chromatogram.

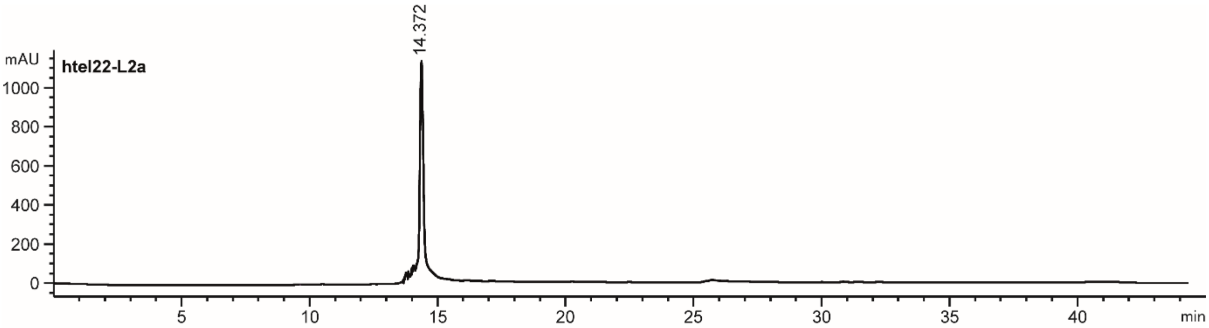

Figure S7. HPLC-trace of oligonucleotide **htel22-L2a** at 5µM concentration in 50mM TEAA buffer.

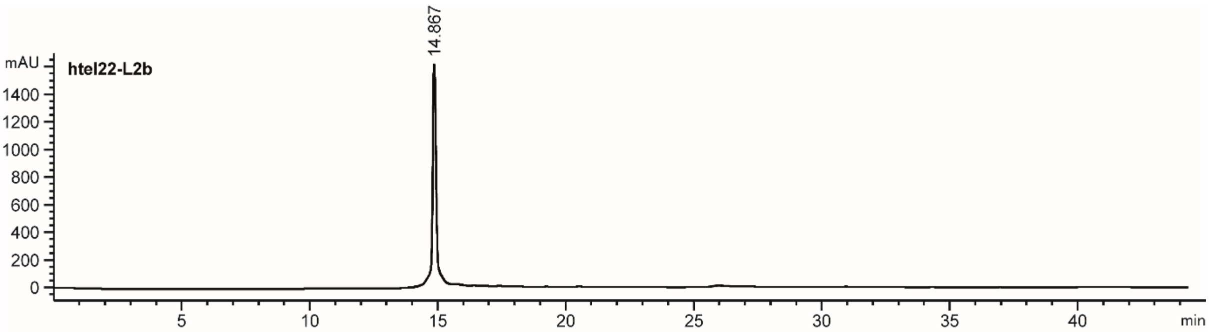

Figure S8. HPLC-trace of oligonucleotide **htel22-L2b** at 5µM concentration in 50mM TEAA buffer.

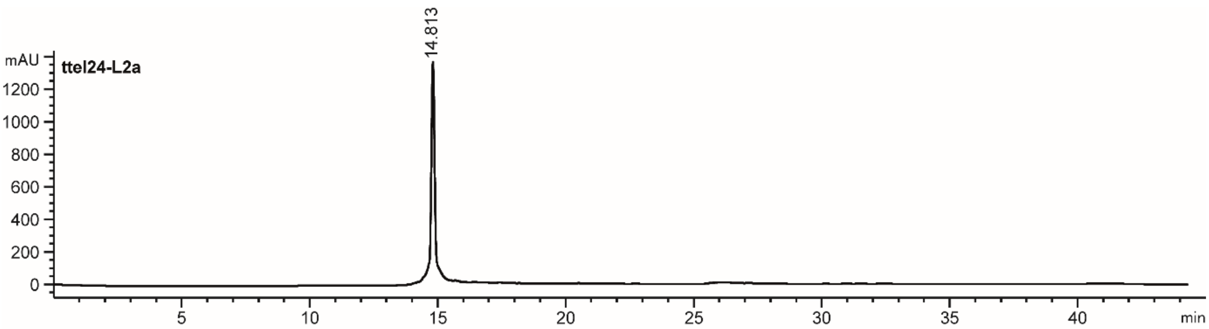

Figure S9. HPLC-trace of oligonucleotide **ttel24-L2a** at 5µM concentration in 50mM TEAA buffer.

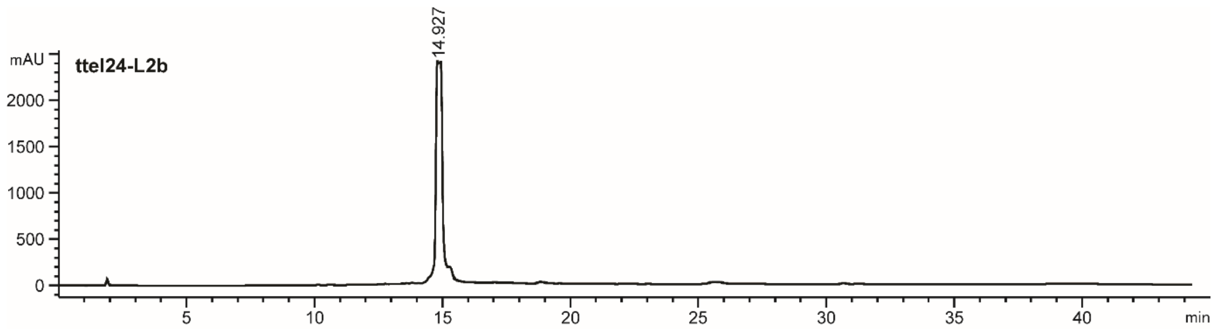

Figure S10. HPLC-trace of oligonucleotide **ttel24-L2b** at 5μM concentration in 50mM TEAA buffer.

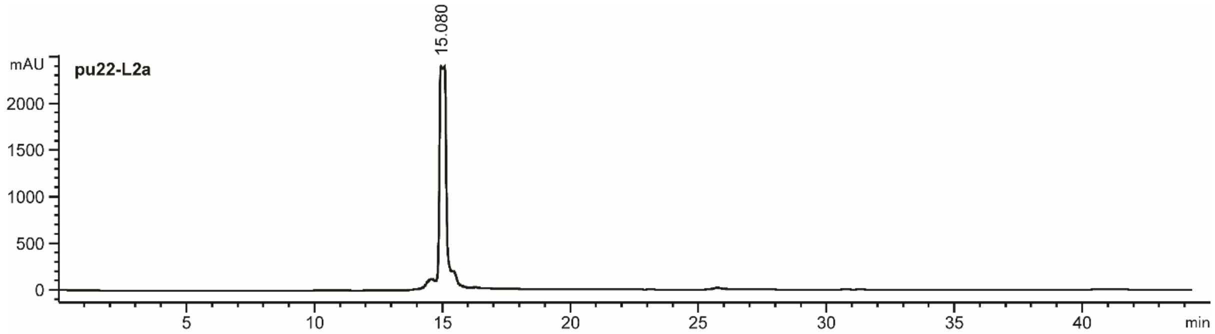

Figure S11. HPLC-trace of oligonucleotide **pu22-L2a** at 5μM concentration in 50mM TEAA buffer.

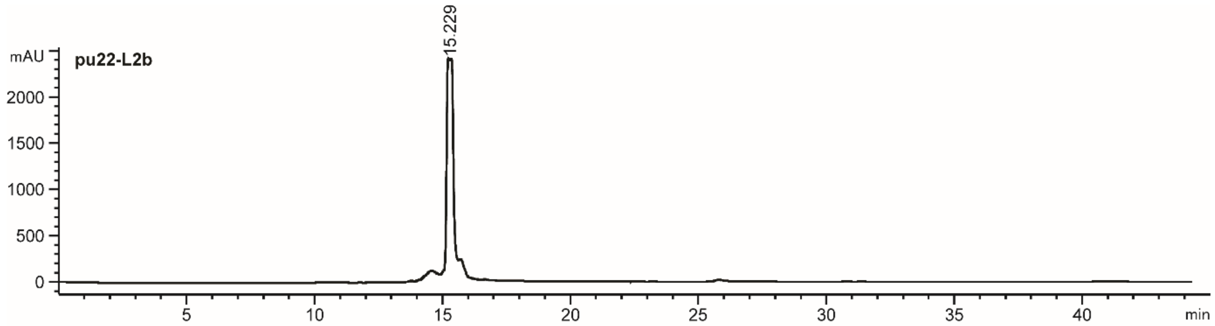

Figure S12. HPLC-trace of oligonucleotide **pu22-L2b** at 5μM concentration in 50mM TEAA buffer.

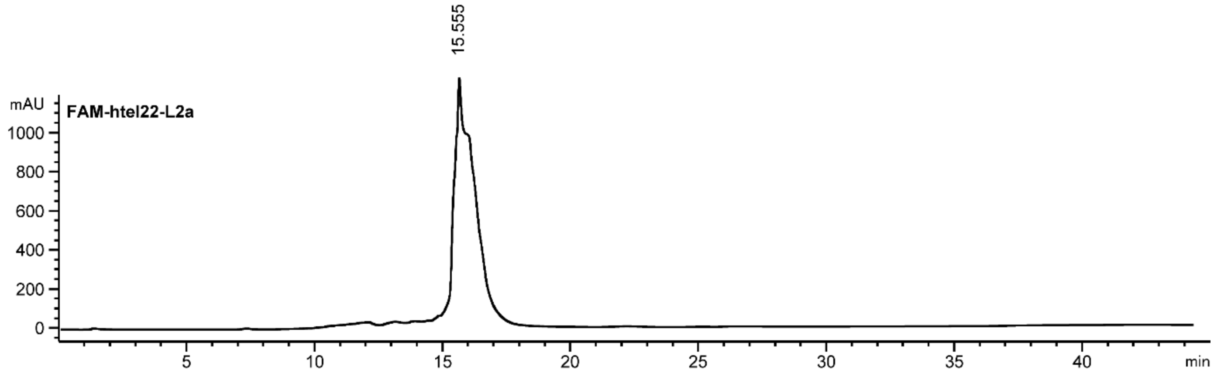

Figure S13. HPLC-trace of oligonucleotide **FAM-htel22-L2a** at 5μM concentration in 50mM TEAA buffer.

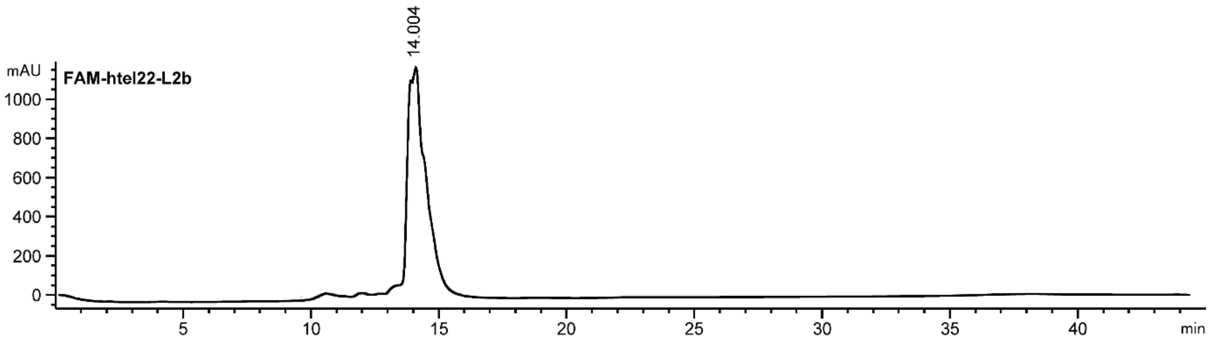

Figure S14. HPLC-trace of oligonucleotide **FAM-htel22-L2b** at 5μM concentration in 50mM TEAA buffer.

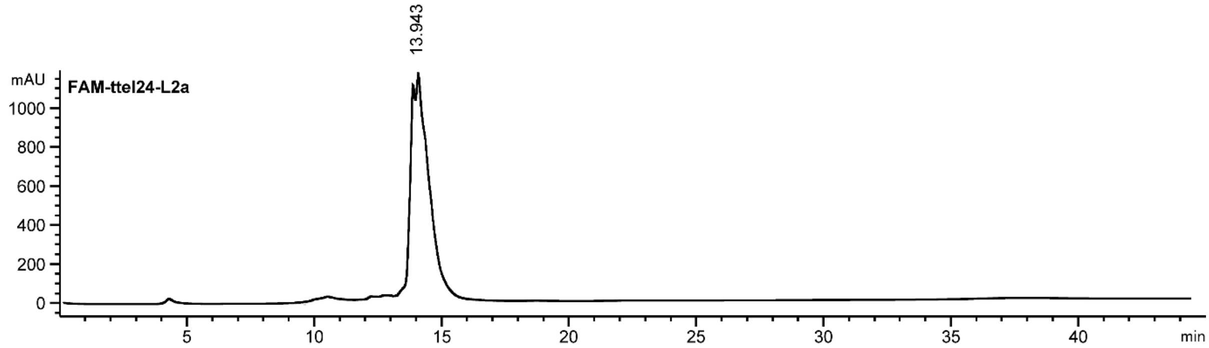

Figure S15. HPLC-trace of oligonucleotide **FAM-ttel24-L2a** at 5μM concentration in 50mM TEAA buffer.

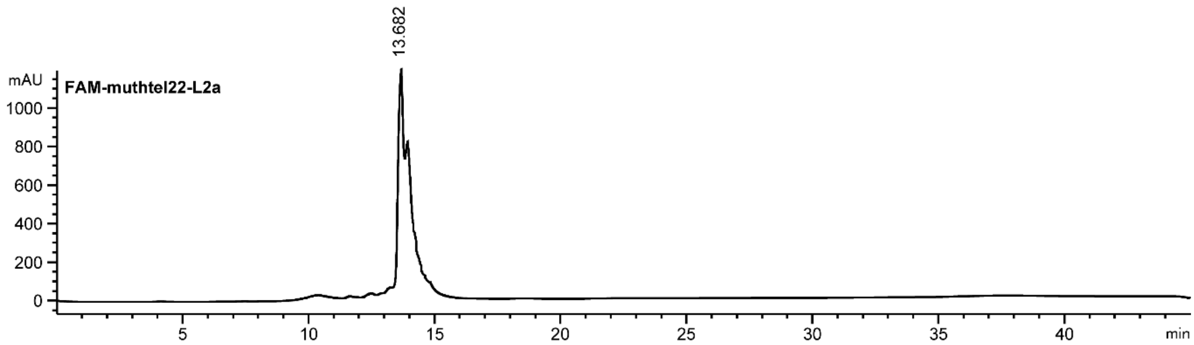

Figure S16. HPLC-trace of oligonucleotide FAM-muthtel22-L2a at 5μM concentration in 50mM TEAA buffer.

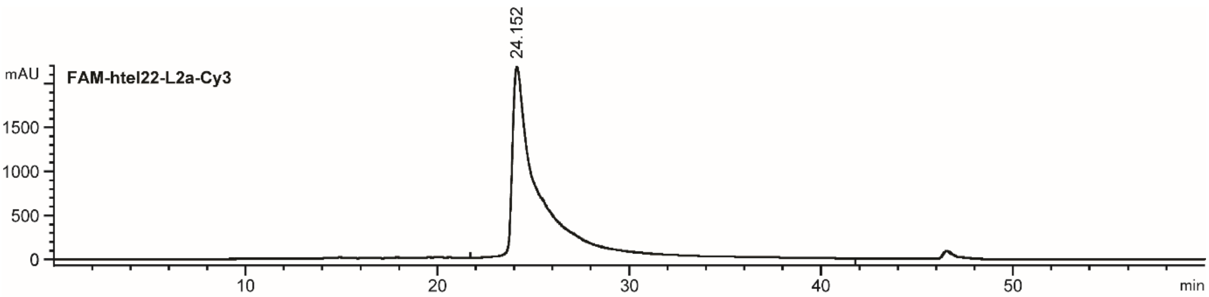

Figure S17. HPLC-trace of oligonucleotide **FAM-htel22-L2a-Cy3** at 5μM concentration in 50mM TEAA buffer.

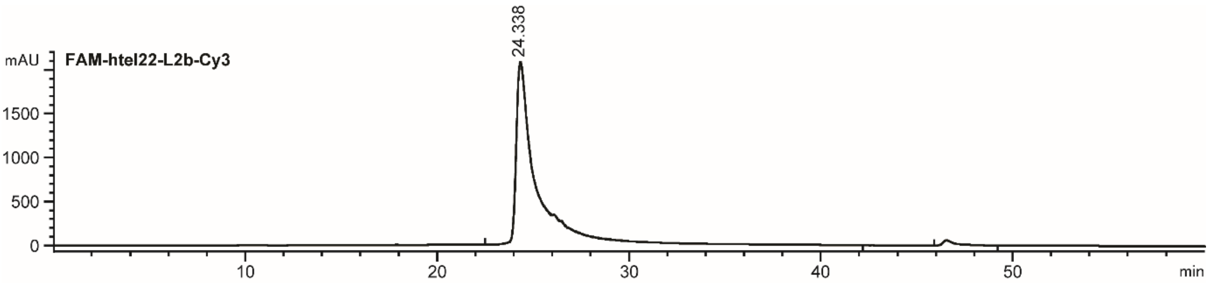

Figure S18. HPLC-trace of oligonucleotide **FAM-htel22-L2b-Cy3** at 5μM concentration in 50mM TEAA buffer.

8. Mass Spectrometry

Electrospray ionization mass spectrometry (ESI-MS) was conducted in negative ionization mode using Bruker timsTOF and Compact instruments. The TOF analyzer was calibrated with Agilent ESI-Low Concentration Tuning Mix. Oligonucleotide samples (5 μL, 5 μM in 10 mM TEAA, pH 7.0) were automatically injected via an Agilent 1260 Infinity autosampler (flow rate: 0.3 mL/min; mobile phase: 1:1 v/v acetonitrile/water). All analyses utilized trifluoroacetate-free buffers to prevent ion suppression. Mass spectra were acquired under standard operating conditions with optimized capillary voltage (−4.5 kV) and nebulizer gas settings.

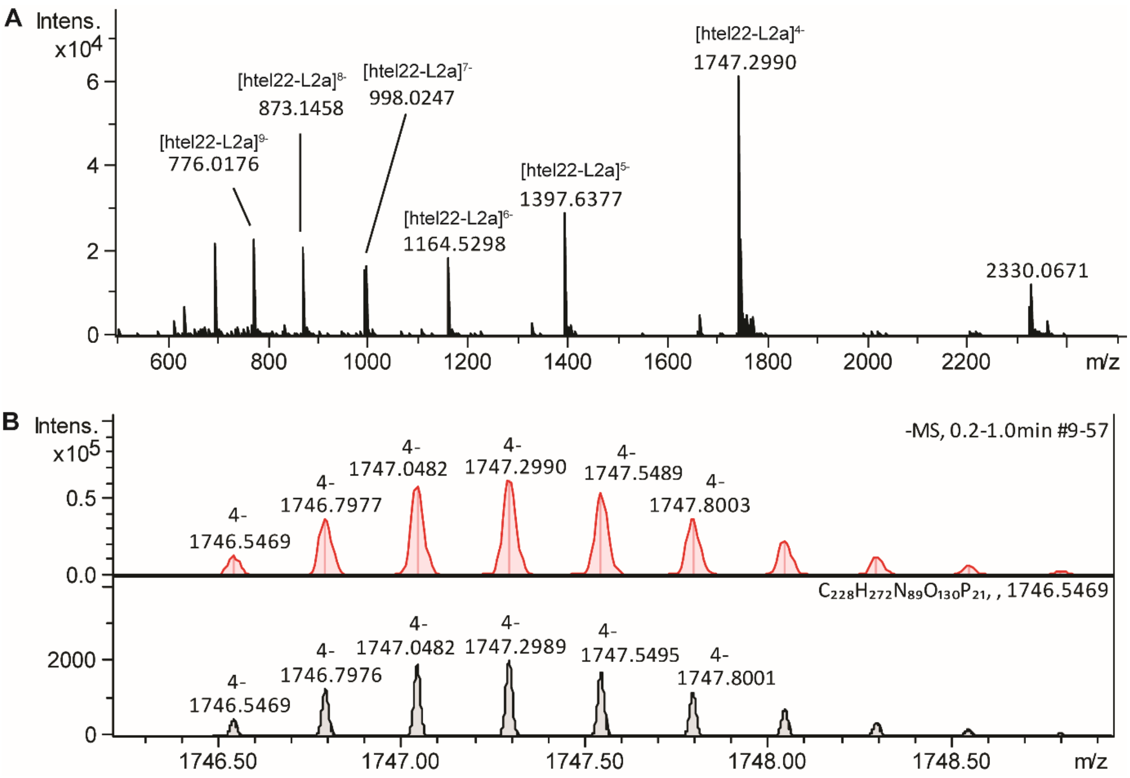

Figure S19. LC-ESI-MS of htel22-L2a oligonucleotide. Conditions: 5uM DNA, 100mM TMAA pH 7.1.

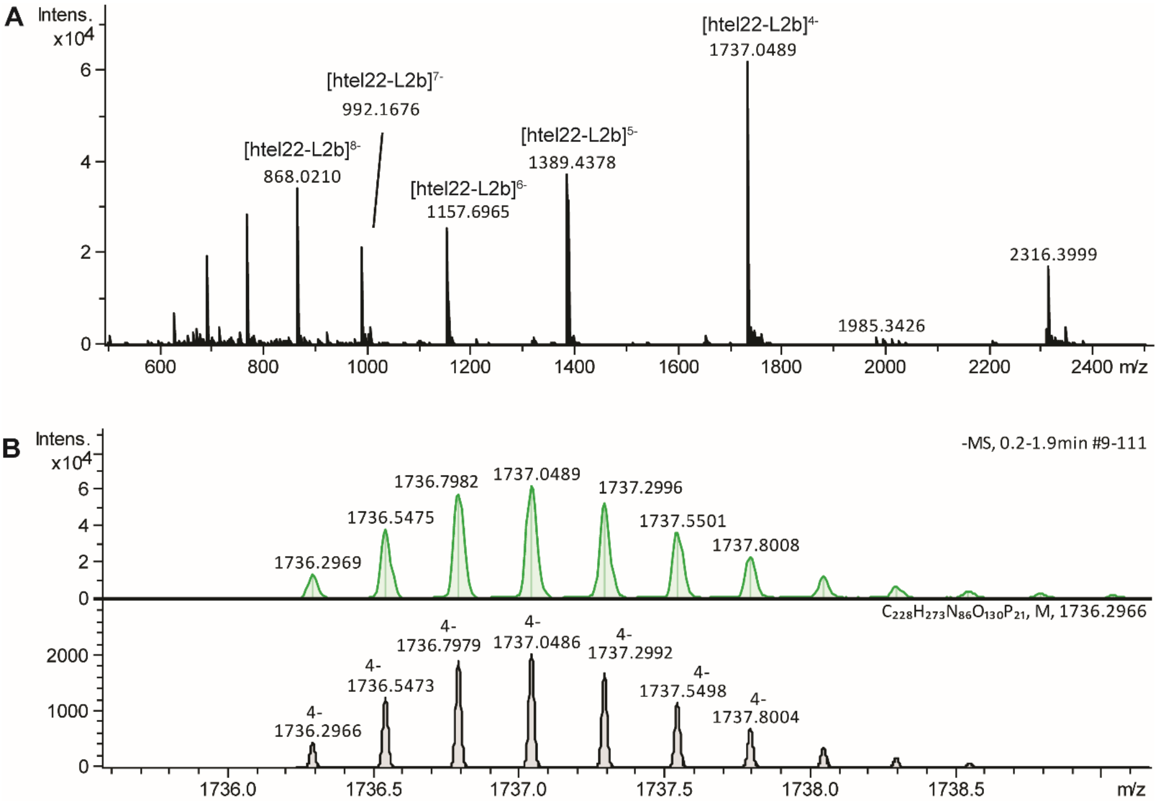

Figure S20. LC-ESI-MS of htel22-L2b oligonucleotide. Conditions: 5uM DNA, 100mM TMAA pH 7.1.

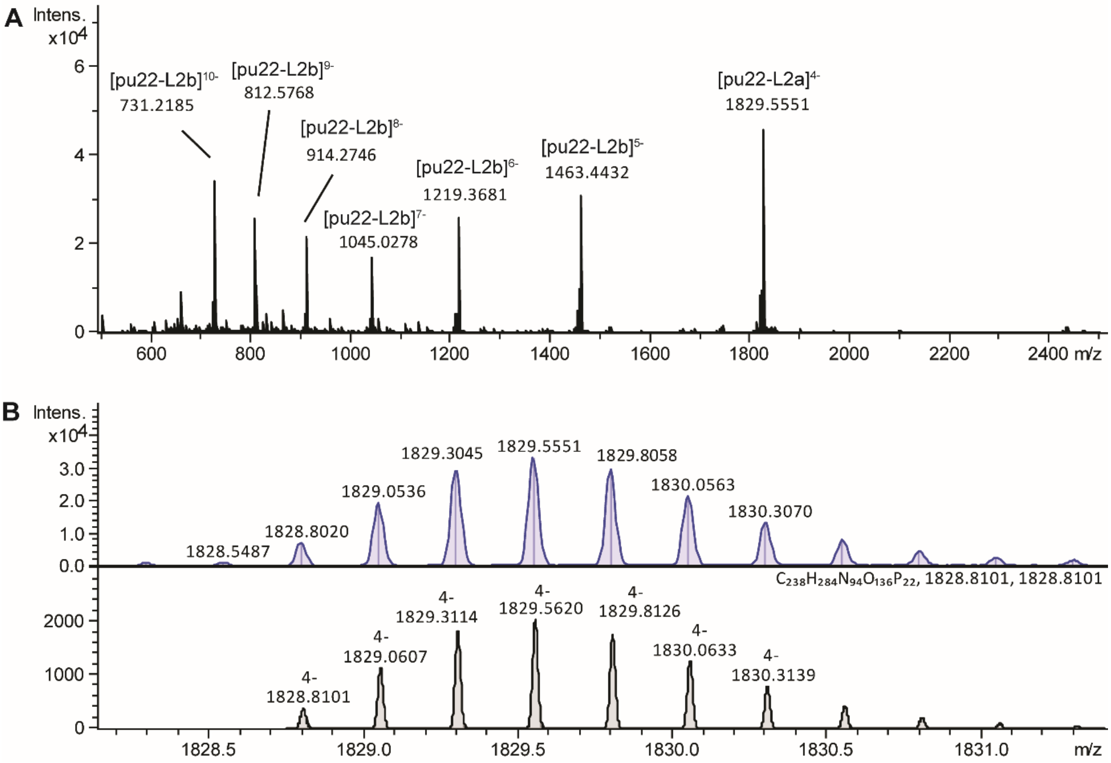

Figure S21. LC-ESI-MS of pu22-L2a oligonucleotide. Conditions: 5uM DNA, 100mM TMAA pH 7.1.

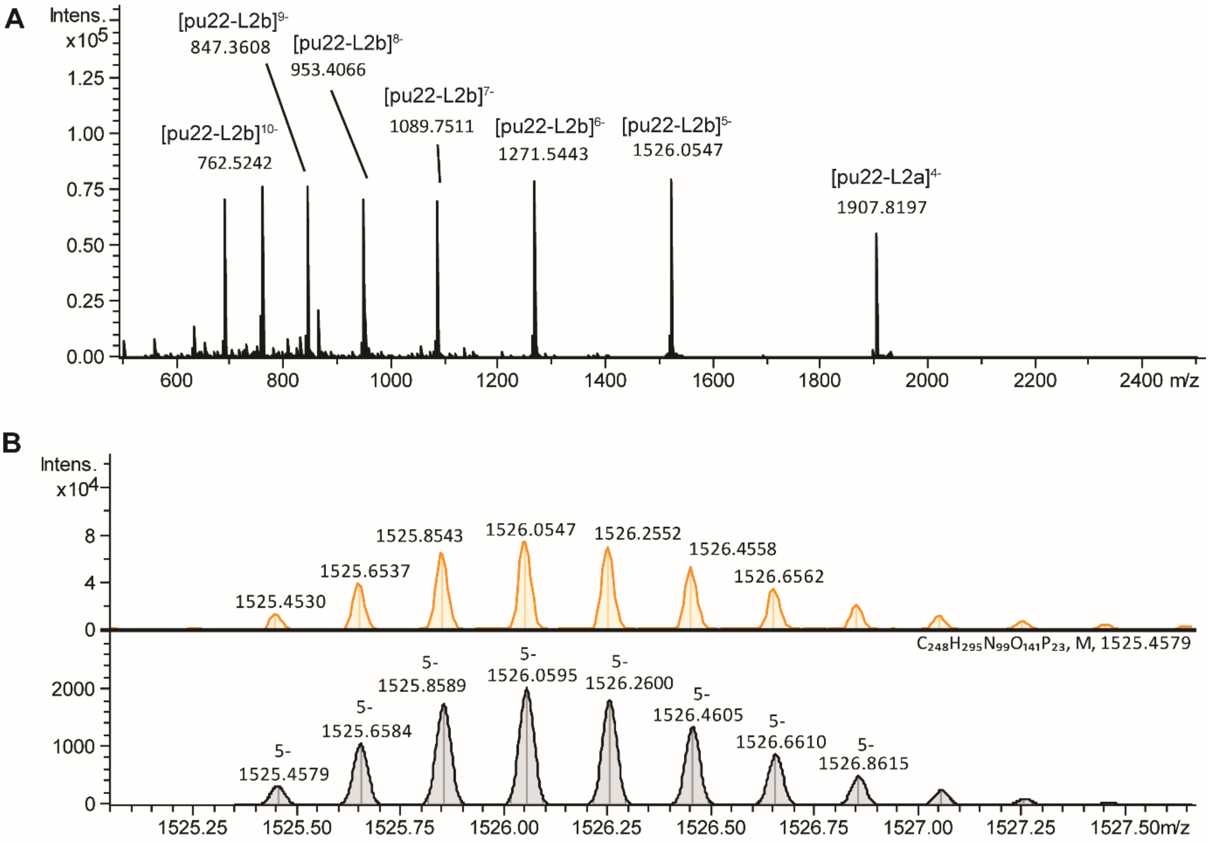

Figure S22. LC-ESI-MS of pu22-L2b oligonucleotide. Conditions: 5uM DNA, 100mM TMAA pH 7.1.

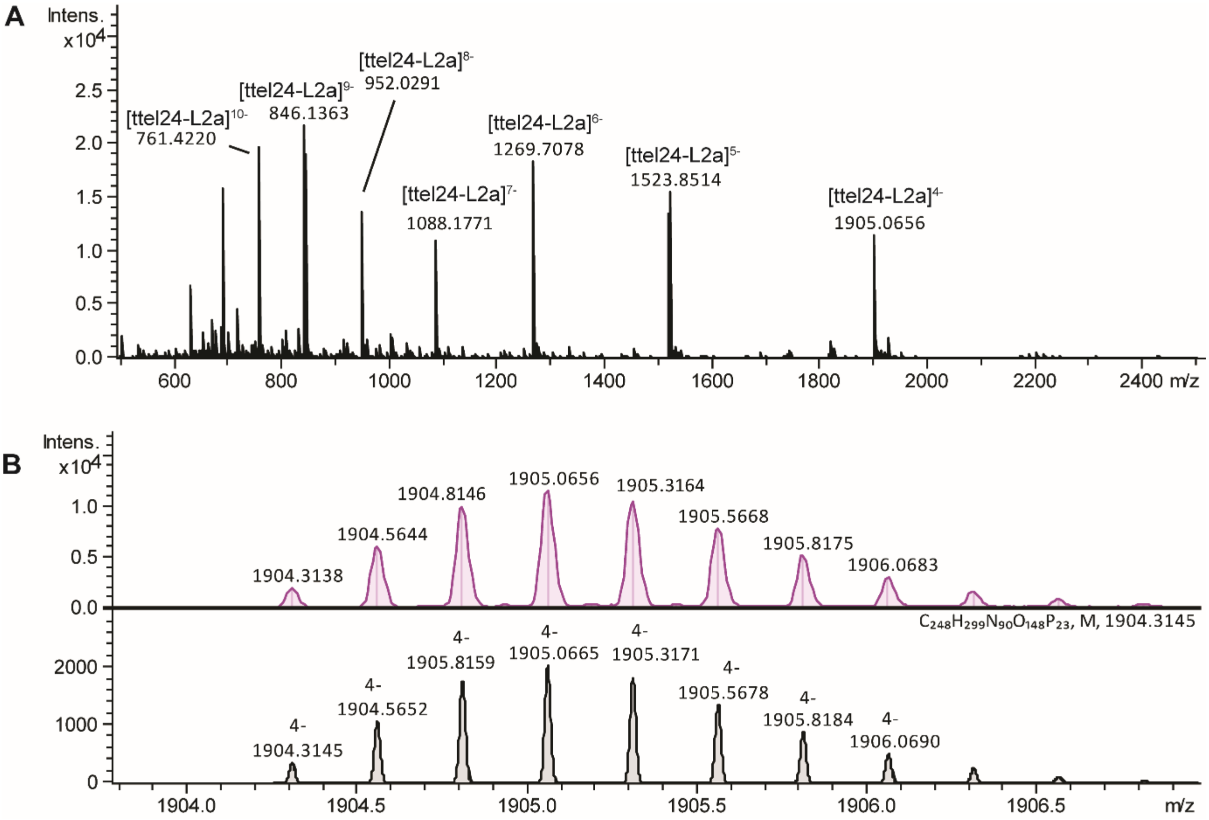

Figure S23. LC-ESI-MS of ttel24-L2a oligonucleotide. Conditions: 5uM DNA, 100mM TMAA pH 7.1.

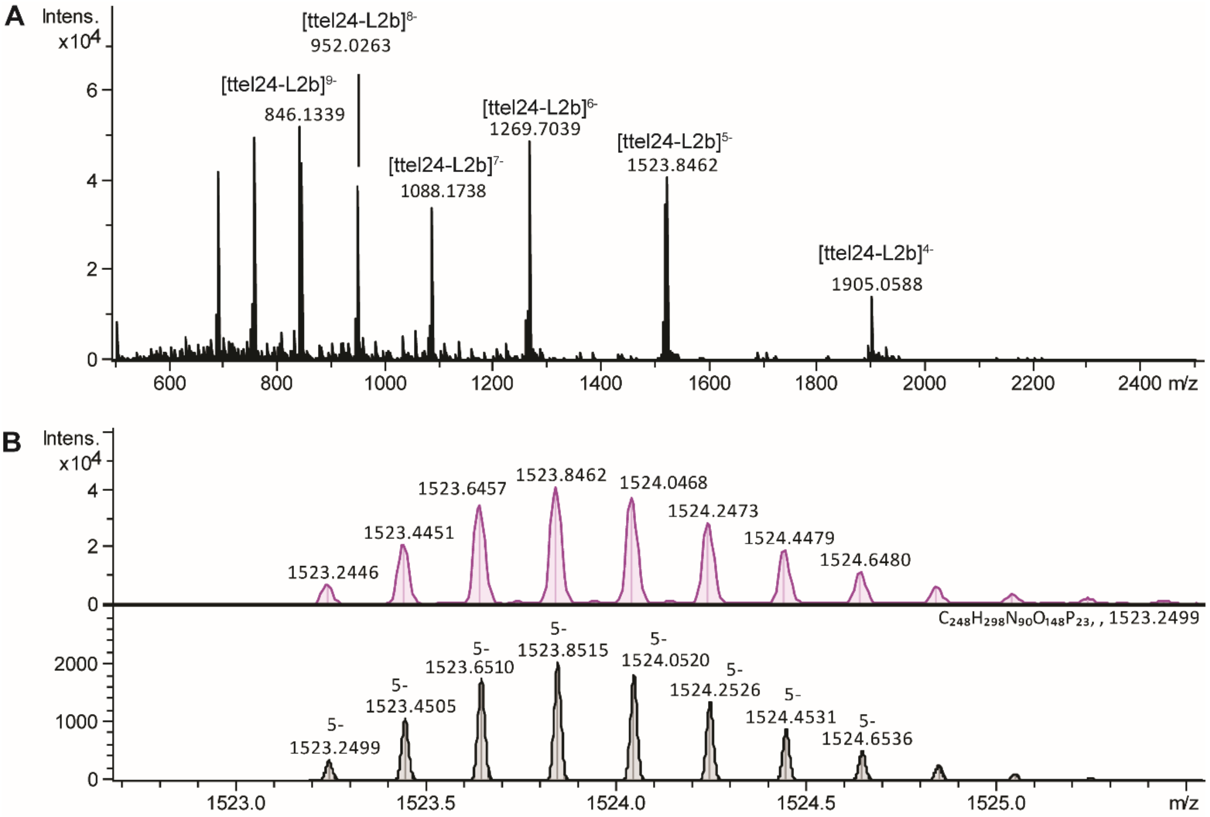

Figure S24. LC-ESI-MS of ttel24-L2b oligonucleotide. Conditions: 5uM DNA, 100mM TMAA pH 7.1.

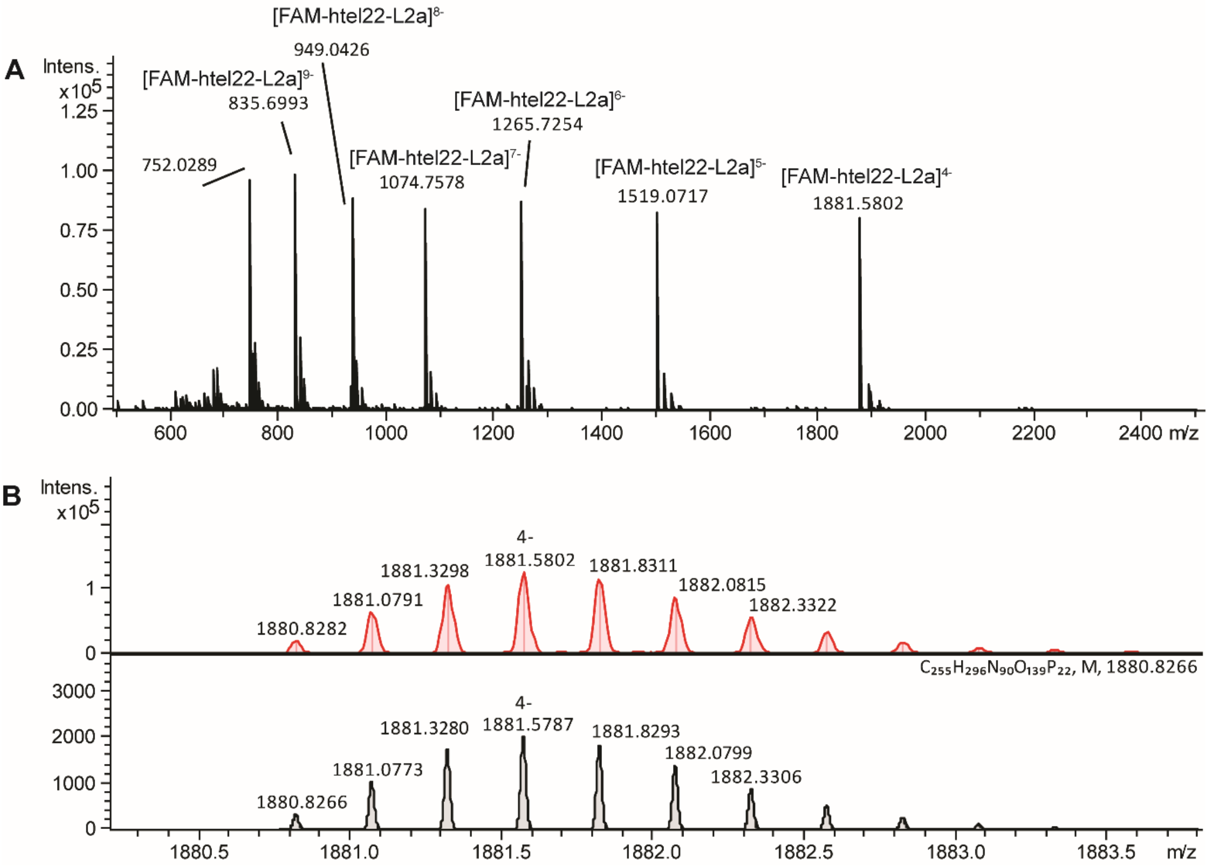

Figure S25. LC-ESI-MS of FAM-htel22-L2a oligonucleotide. Conditions: 5uM DNA, 100mM TMAA pH 7.1.

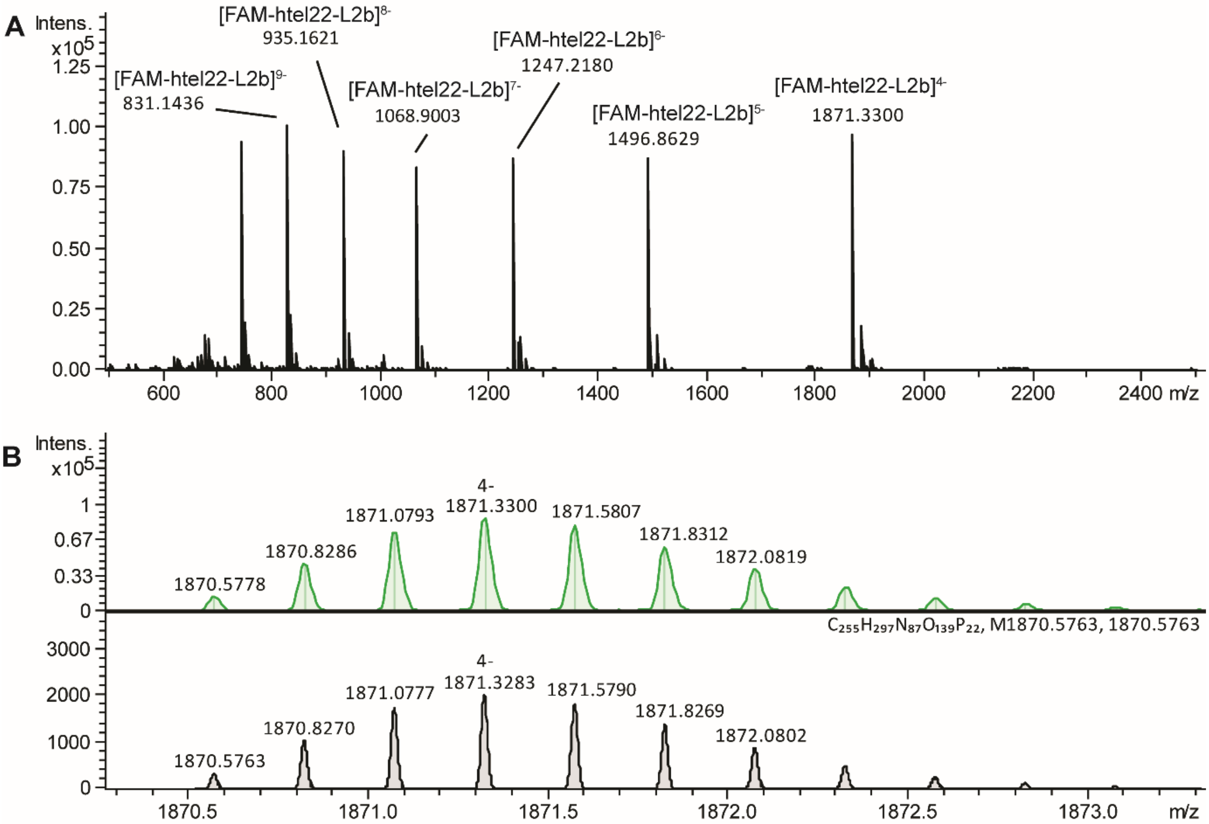

Figure S26. LC-ESI-MS of FAM-htel22-L2b oligonucleotide. Conditions: 5uM DNA, 100mM TMAA pH 7.1.

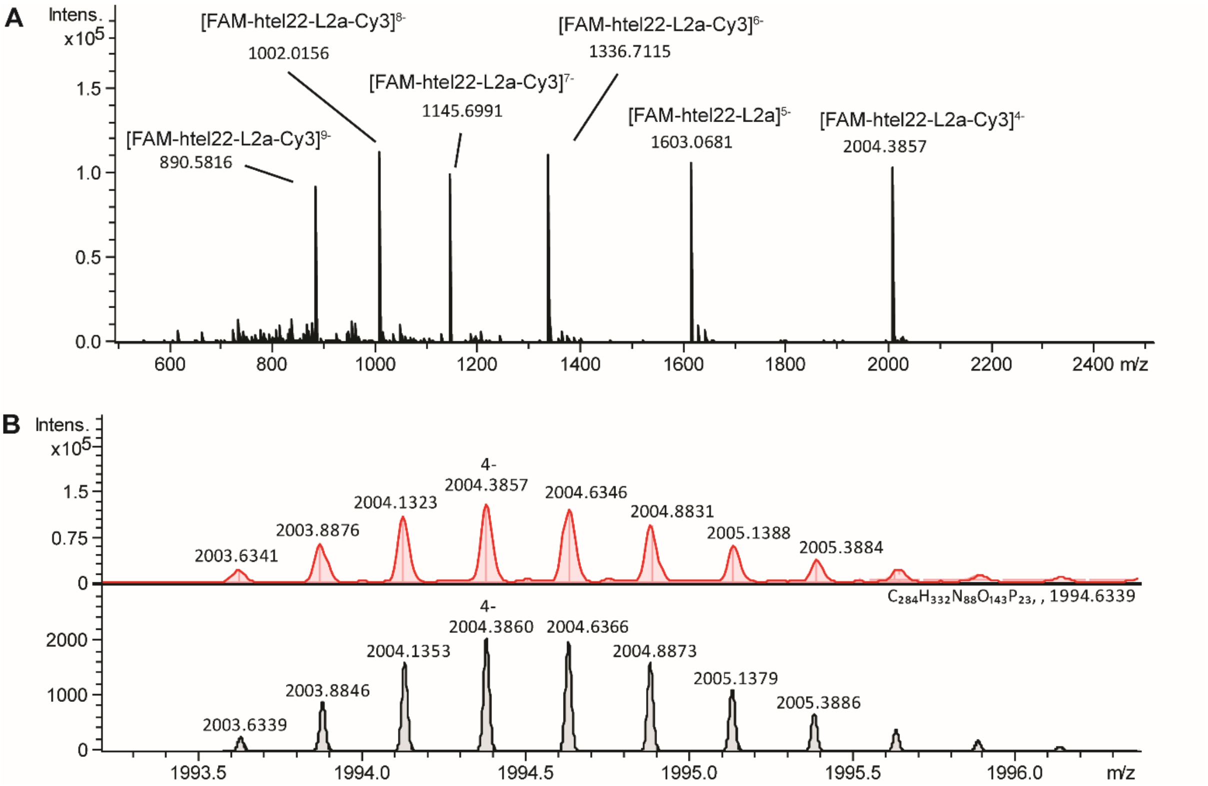

Figure S27. LC-ESI-MS of FAM-htel22-L2a-Cy3 oligonucleotide. Conditions: 5uM DNA, 100mM TMAA pH 7.1.

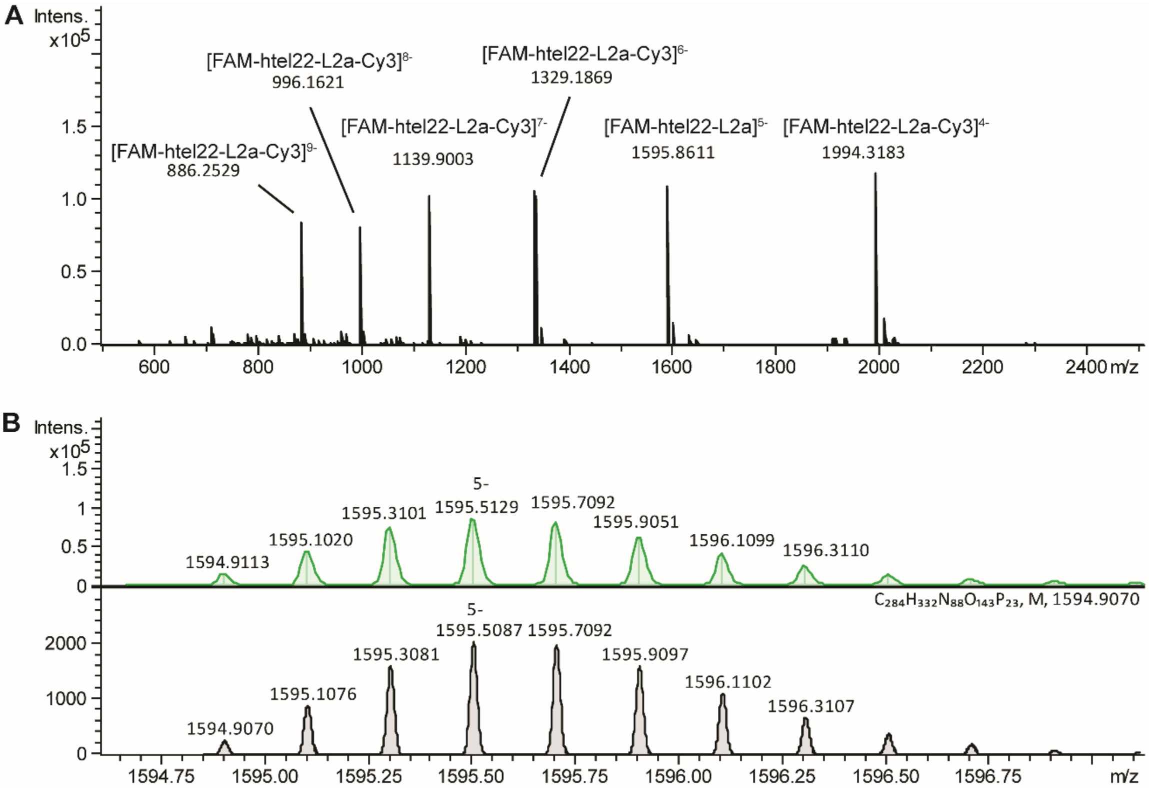

Figure S28. LC-ESI-MS of FAM-htel22-L2b-Cy3 oligonucleotide. Conditions: 5uM DNA, 100mM TMAA pH 7.1.

9. Native Mass spectrometry

Native electrospray ionization mass spectrometry (ESI-MS) was conducted in negative ionization mode using Bruker timsTOF and Compact instruments. The TOF analyzer was calibrated with Agilent ESI-Low Concentration Tuning Mix. Oligonucleotide samples (5 µL, DNA concentration 25 µM in 10 mM TEAA, 1mM KCl, if present 27.5 µM MSO_4 (M = Cu, Ni, Zn) pH 7.0, annealed under standard conditions) were manually injected via (diluted 1:1 v/v acetonitrile/DNA sample). All analyses utilized trifluoroacetate-free buffers to prevent ion suppression. Mass spectra were acquired under standard operating conditions with optimized capillary voltage (−4.5 kV) and nebulizer gas settings.

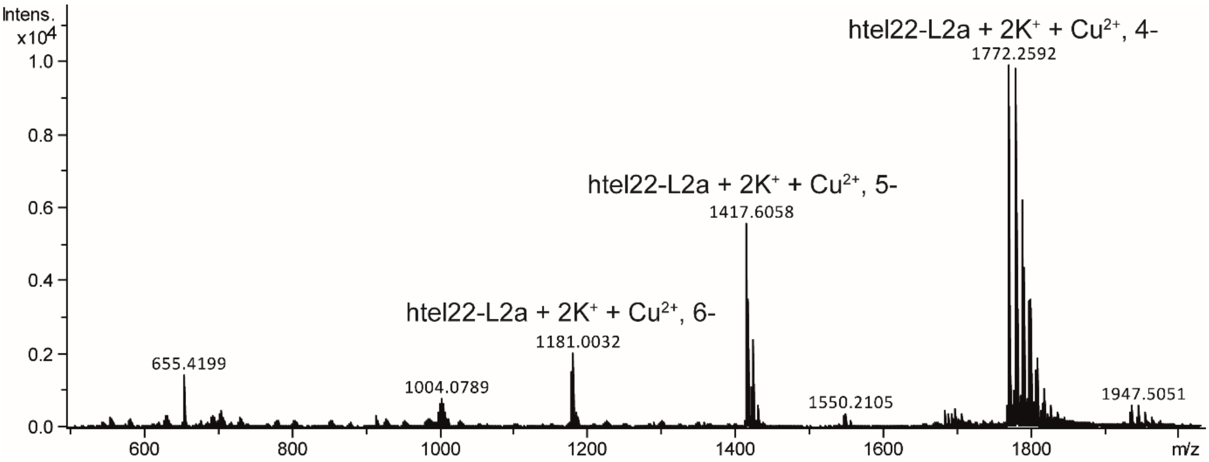

Figure S29. Native ESI-MS of htel22-L2a in presence of Cu^{2+}. Conditions: 12.5 µM DNA, 15 µM $CuSO_4$, 0.5 mM KCl, 50 mM TMAA pH 7.1, H_2O:ACN, 1:1.

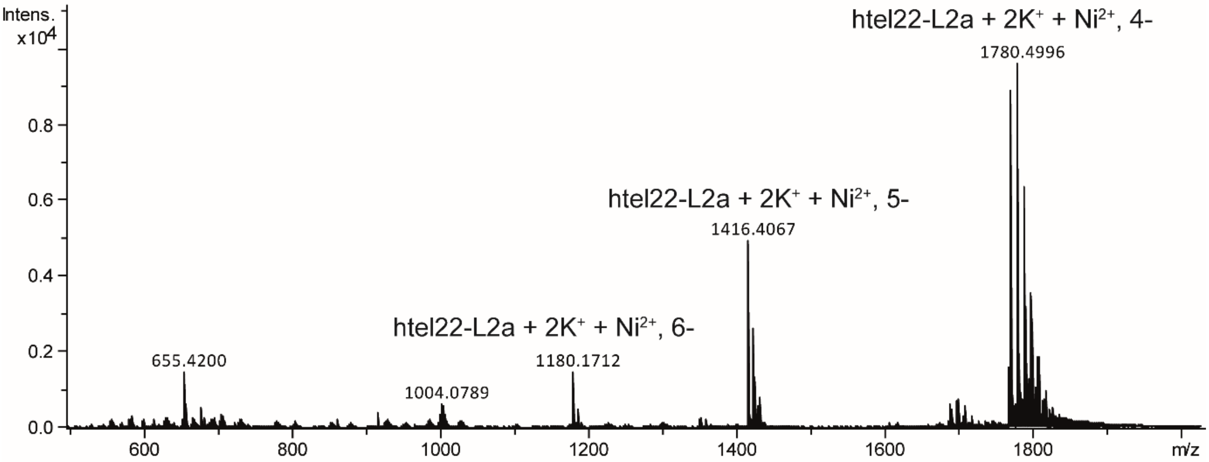

Figure S30. Native ESI-MS of htel22-L2a in presence of Ni^{2+}. Conditions: 12.5 µM DNA, 15 µM $CuSO_4$, 0.5 mM KCl, 50 mM TMAA pH 7.1, H_2O:ACN, 1:1.

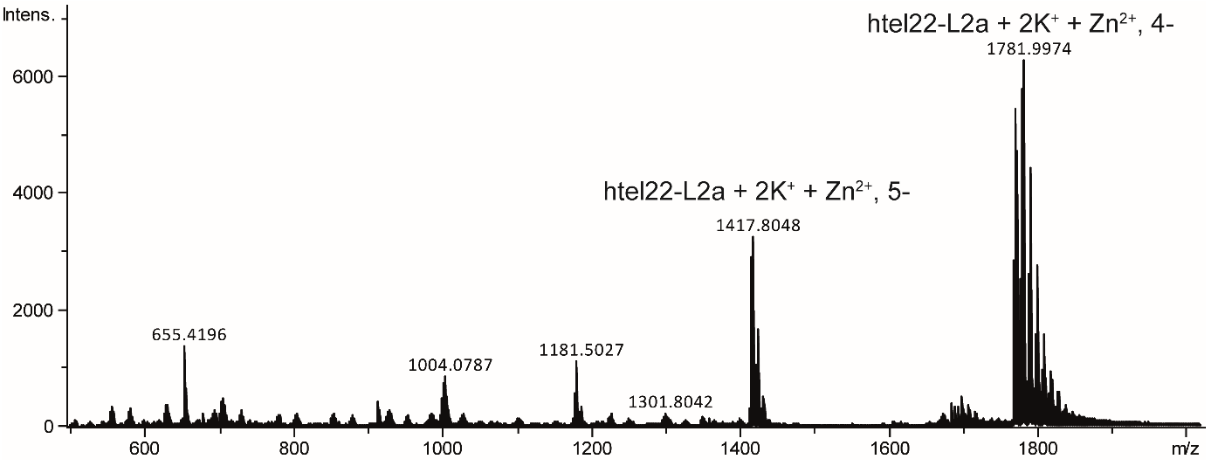

Figure S31. Native ESI-MS of htel22-L2a in presence of Zn^{2+}. Conditions: 12.5 μM DNA, 15 μM $CuSO_4$, 0.5 mM KCl, 50 mM TMAA pH 7.1, H_2O:ACN, 1:1.

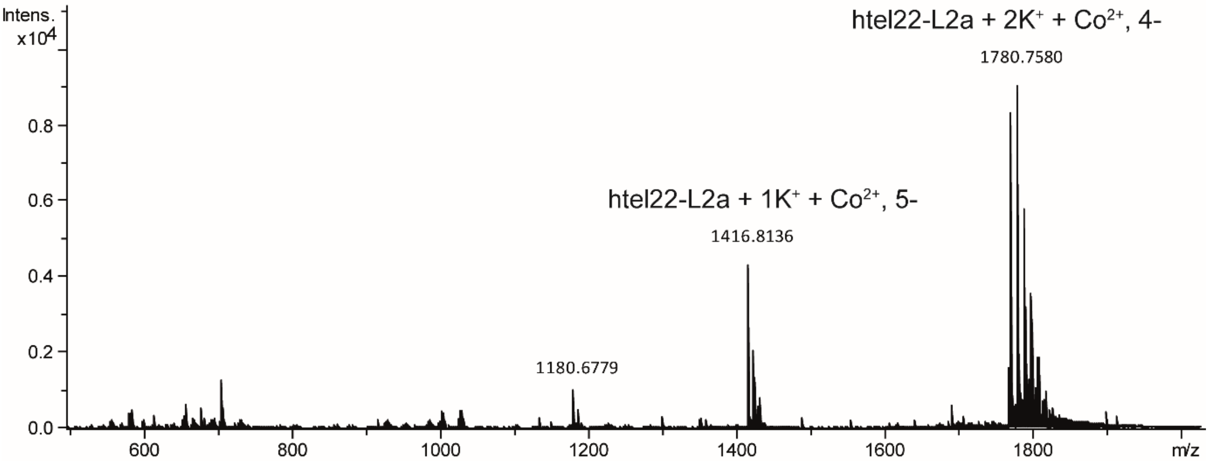

Figure S32. Native ESI-MS of htel22-L2a in presence of Co^{2+}. Conditions: 12.5 μM DNA, 15 μM $CuSO_4$, 0.5 mM KCl, 50 mM TMAA pH 7.1, H_2O:ACN, 1:1.

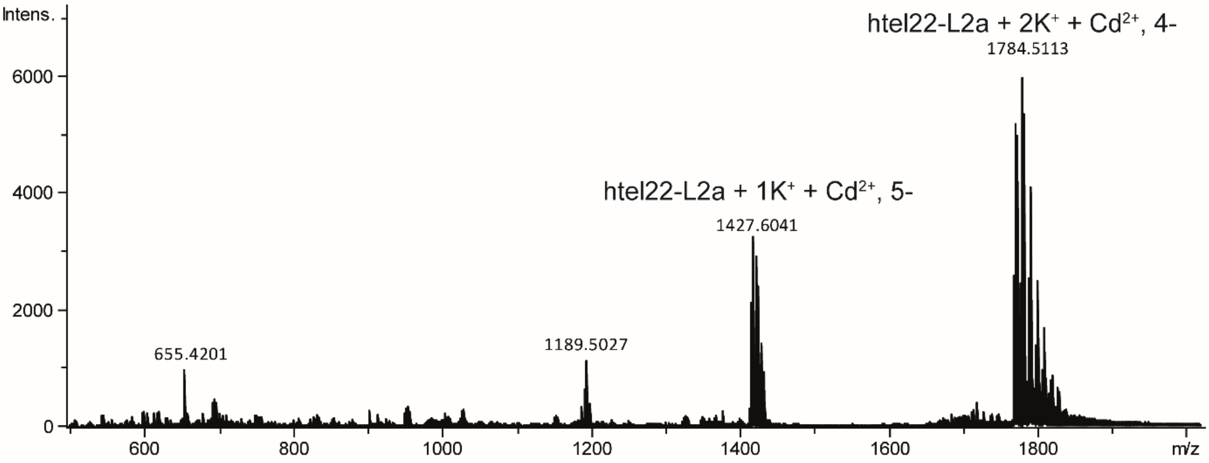

Figure S33. Native ESI-MS of htel22-L2a in presence of Cd^{2+}. Conditions: 12.5 μM DNA, 15 μM $CuSO_4$, 0.5 mM KCl, 50 mM TMAA pH 7.1, H_2O:ACN, 1:1.

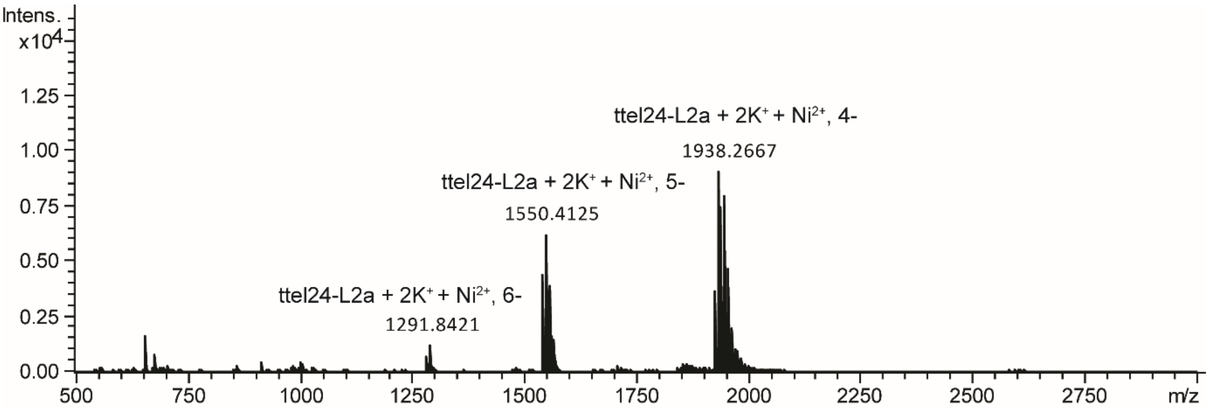

Figure S34. Native ESI-MS of ttel24-L2a in presence of Ni^{2+}. Conditions: 12.5 μM DNA, 15 μM $CuSO_4$, 0.5 mM KCl, 50 mM TMAA pH 7.1, H_2O:ACN, 1:1.

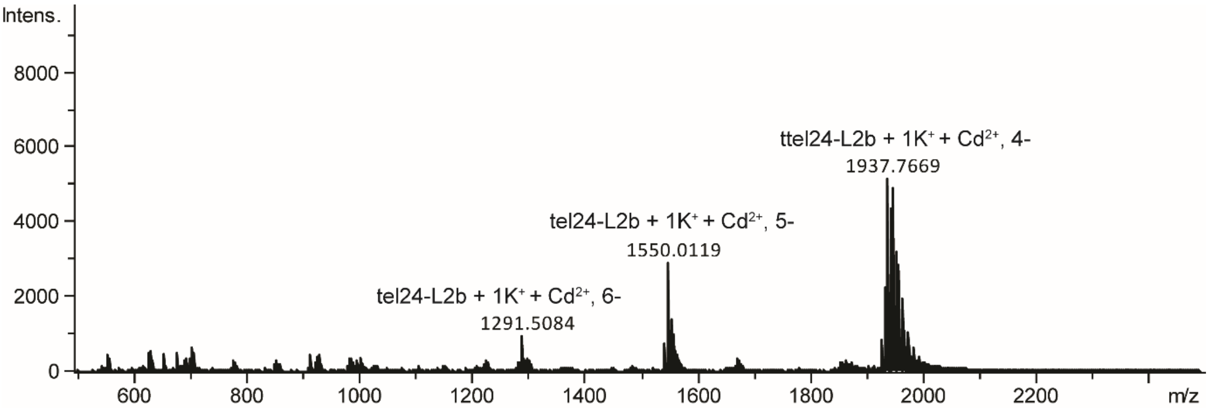

Figure S35. Native ESI-MS of ttel24-L2b in presence of Cd^{2+}. Conditions: 12.5 μM DNA, 15 μM $CuSO_4$, 0.5 mM KCl, 50 mM TMAA pH 7.1, H_2O:ACN, 1:1.

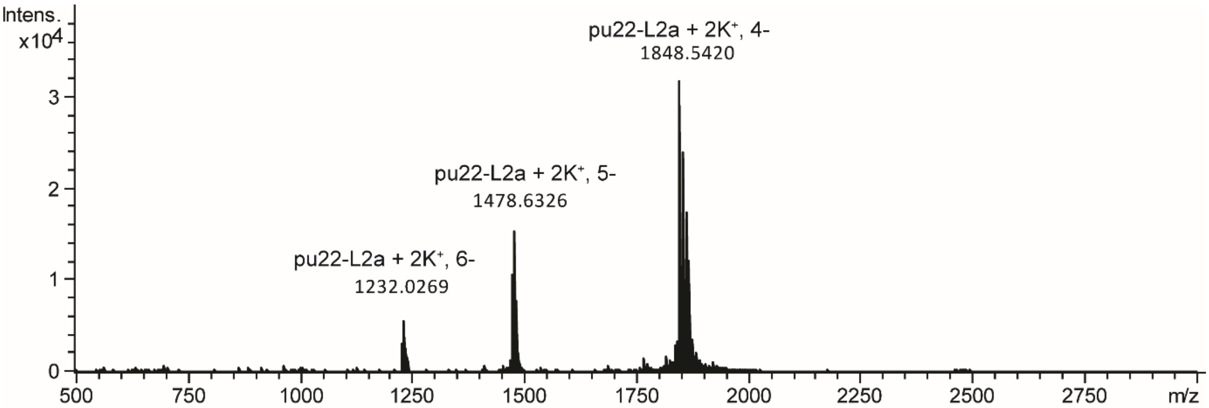

Figure S36. Native ESI-MS of pu22-L2a in absence of M^{2+} (Cu^{2+}, Ni^{2+}, Zn^{2+}). Conditions: 12.5 μM DNA, 0.5 mM KCl, 50 mM TMAA pH 7.1, H_2O:ACN, 1:1.

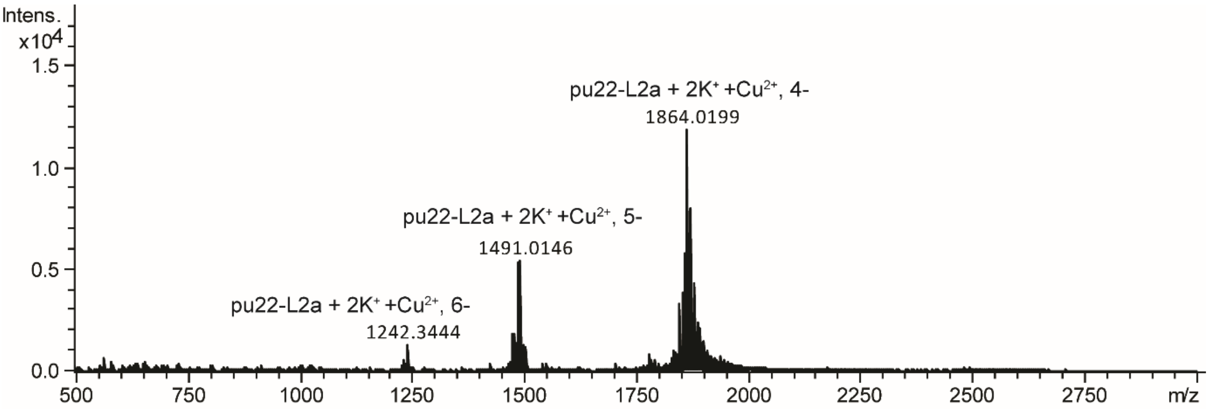

Figure S37. Native ESI-MS of pu22-L2a in presence of Cu^{2+}. Conditions: 12.5 µM DNA, 15 µM $CuSO_4$, 0.5 mM KCl, 50 mM TMAA pH 7.1, H_2O:ACN, 1:1.

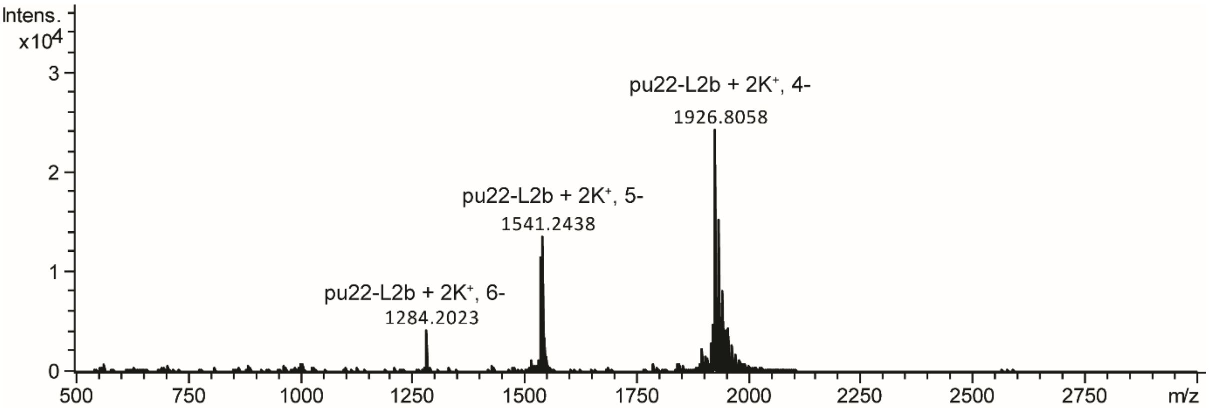

Figure S38. Native ESI-MS of pu22-L2b in absence of M2+ (Cu2+, Ni2+, Zn2+). Conditions: 12.5 µM DNA, 0.5 mM KCl, 50 mM TMAA pH 7.1, H2O:ACN, 1:1.

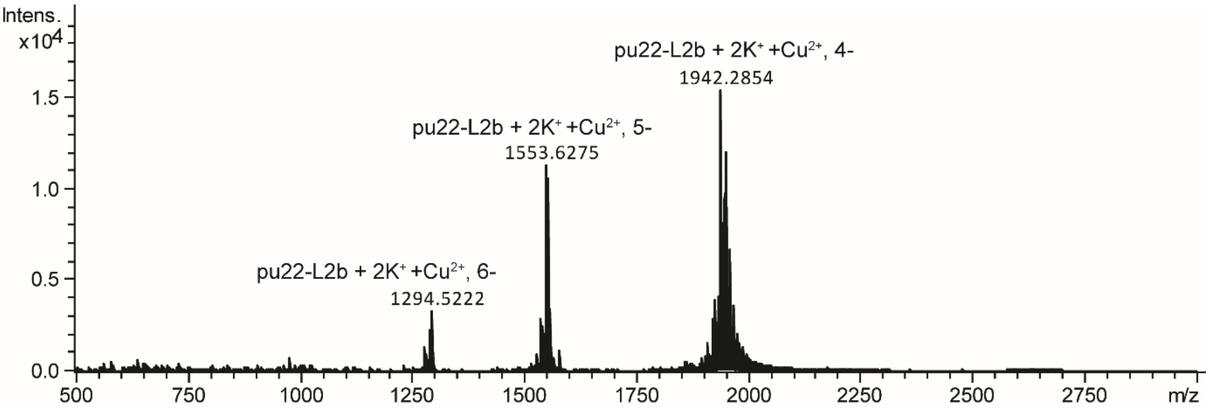

Figure S39. Native ESI-MS of pu22-L2b in presence of Cu^{2+}. Conditions: 12.5 µM DNA, 15 µM $CuSO_4$, 0.5 mM KCl, 50 mM TMAA pH 7.1, H_2O:ACN, 1:1.

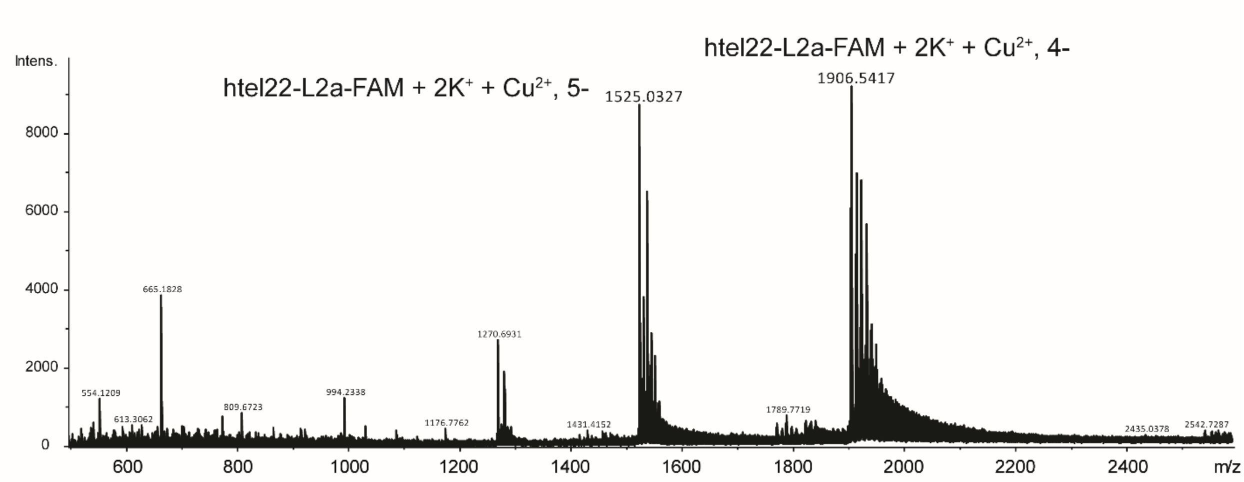

Figure S40. Native ESI-MS of FAM-htel22-L2a in presence of Cu^{2+}. Conditions: 12.5 µM DNA, 15 µM $CuSO_4$, 0.5 mM KCl, 50 mM TMAA pH 7.1, H_2O:ACN, 1:1.

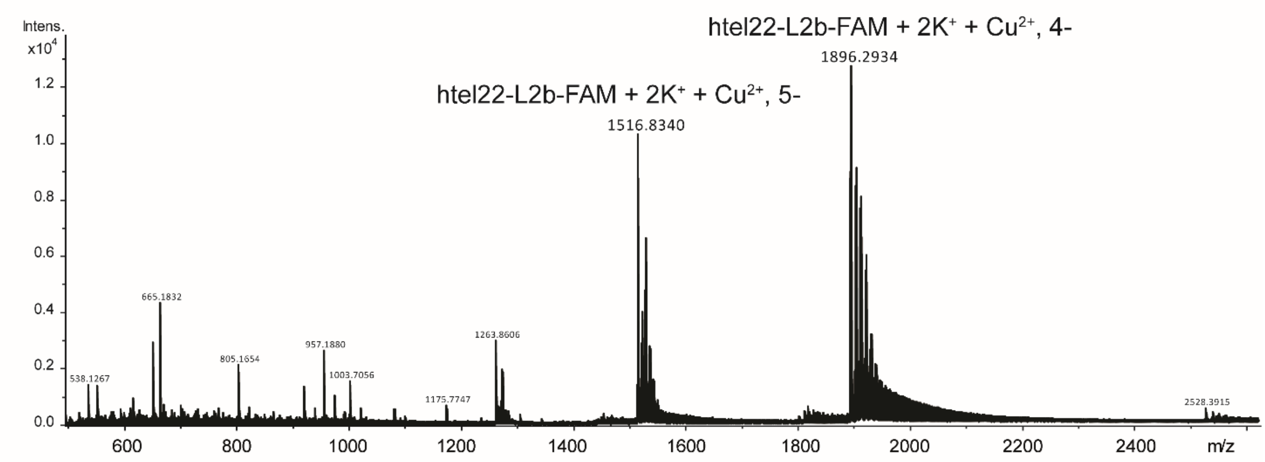

Figure S41. Native ESI-MS of FAM-htel22-L2b in presence of Cu^{2+}. Conditions: 12.5 µM DNA, 15 µM $CuSO_4$, 0.5 mM KCl, 50 mM TMAA pH 7.1, H_2O:ACN, 1:1.

10. Circular Dichroism (CD) spectroscopic analysis

CD spectra were recorded on an Applied Photophysics Chirascan qCD spectropolarimeter over a wavelength range of 350–205 nm (or 700–205 nm for induced CD detection), using a 1 nm step size, 0.5 s acquisition time per point, a bandwidth of 0.5 nm, and three accumulations. Measurements were performed at 4 or 25 °C with temperature regulation provided by a Quantum Northwest controller coupled to the sample probe. Background spectra were collected in the same cuvette used for the sample. To prevent condensation on the cuvette surface or cell window, a continuous nitrogen gas flow was applied. All spectra were averaged, corrected for background (cuvette, buffer, and electrolyte), smoothed using a Savitzky–Golay filter (window size 5), and baseline-adjusted to zero at 350 nm.

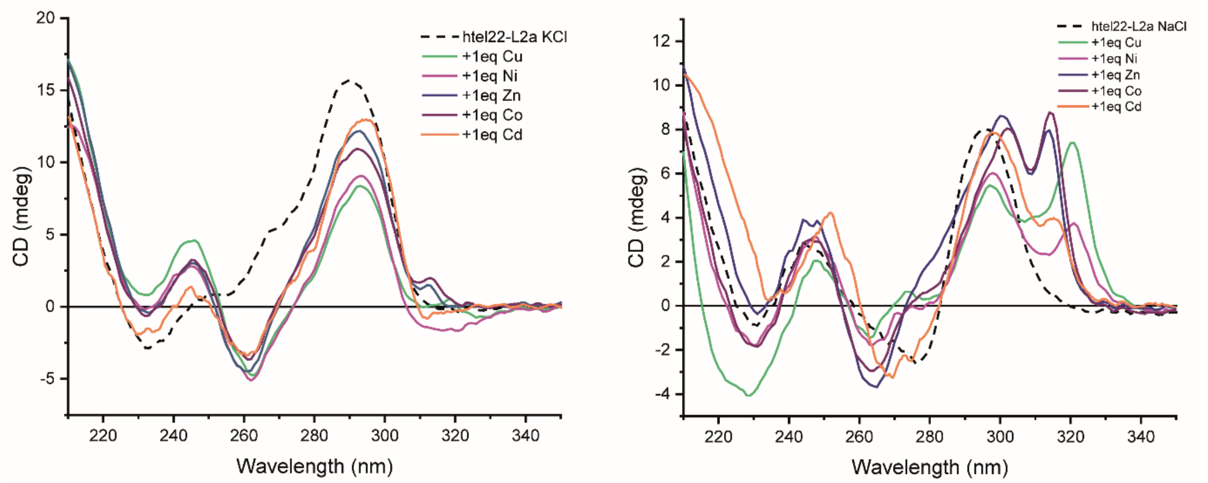

Figure S42. CD spectra of htel22-L2a in absence or presence of various divalent metal cations. Conditions: 4μM DNA, 10mM HEPES, 100mM NaCl or KCl, 4.4 μM M^{2+} (M = Cu, Ni, Zn, Co, Cd).

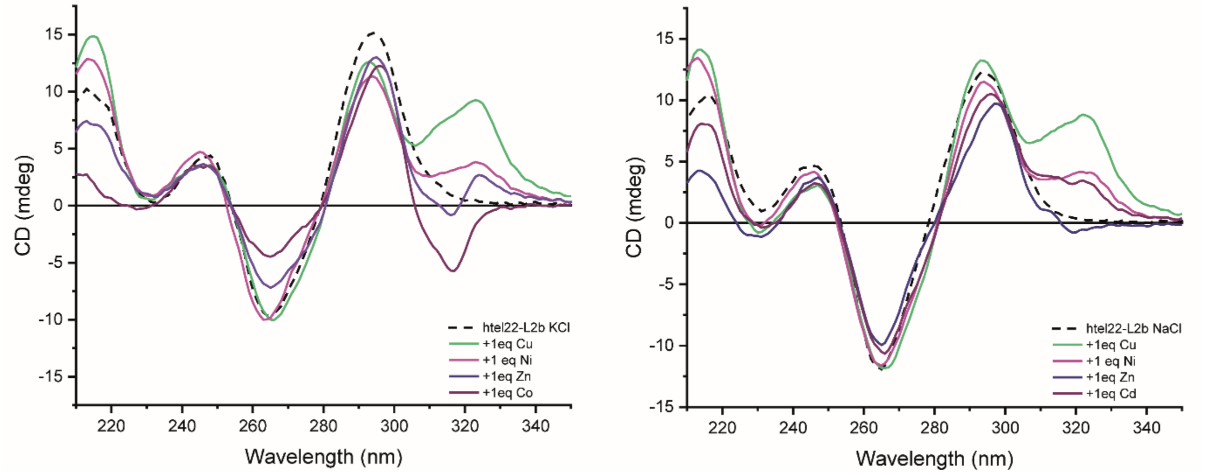

Figure S43. CD spectra of htel22-L2b in absence or presence of various divalent metal cations. Conditions: 4μM DNA, 10mM HEPES, 100mM NaCl or KCl, 4.4 μM M^{2+} (M = Cu, Ni, Zn, Co).

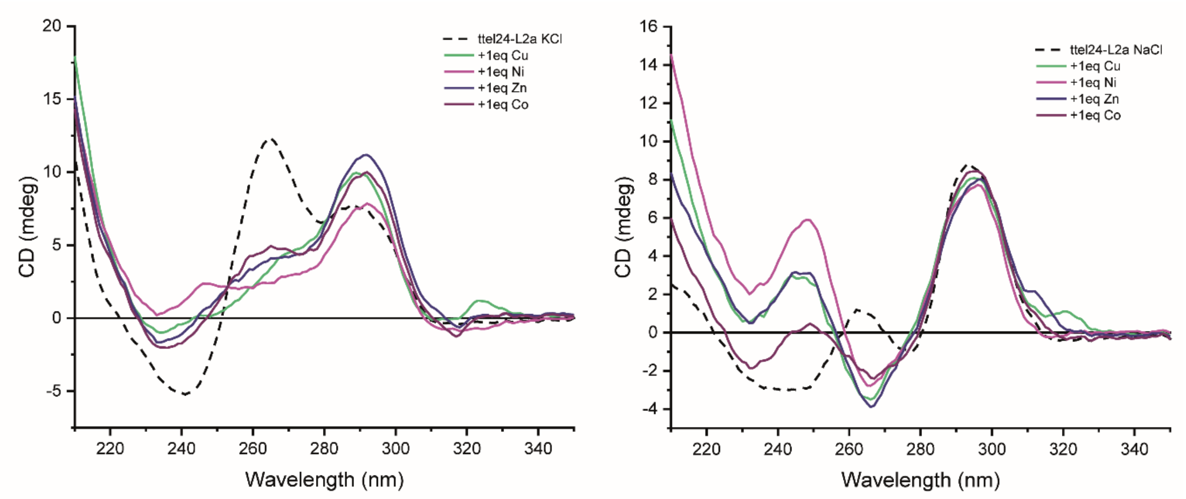

Figure S44. CD spectra of ttel24-L2a in absence or presence of various divalent metal cations. Conditions: 4μM DNA, 10mM HEPES, 100mM NaCl or KCl, 4.4 μM M^{2+} (M = Cu, Ni, Zn, Co).

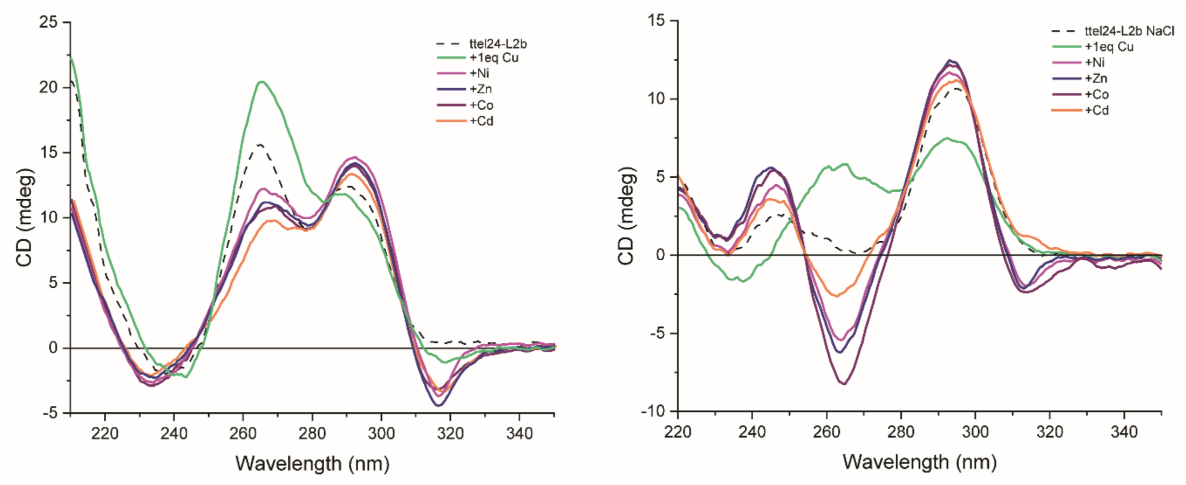

Figure S45. CD spectra of ttel24-L2b in absence or presence of various divalent metal cations. Conditions: 4μM DNA, 10mM HEPES, 100mM NaCl or KCl, 4.4 μM M^{2+} (M = Cu, Ni, Zn, Co, Cd).

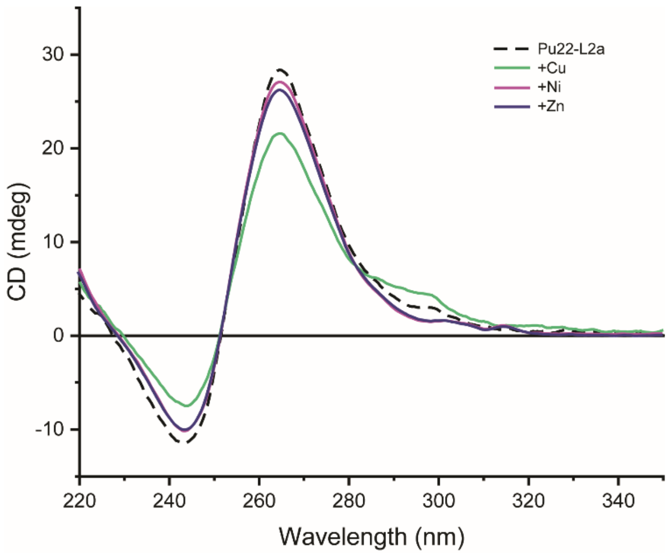

Figure S46. CD spectrum of pu22-L2a in absence or presence of various divalent metal cations. Conditions: 4μM DNA, 10mM HEPES, 100mM KCl, 4.4 μM M^{2+} (M = Cu, Ni, Zn).

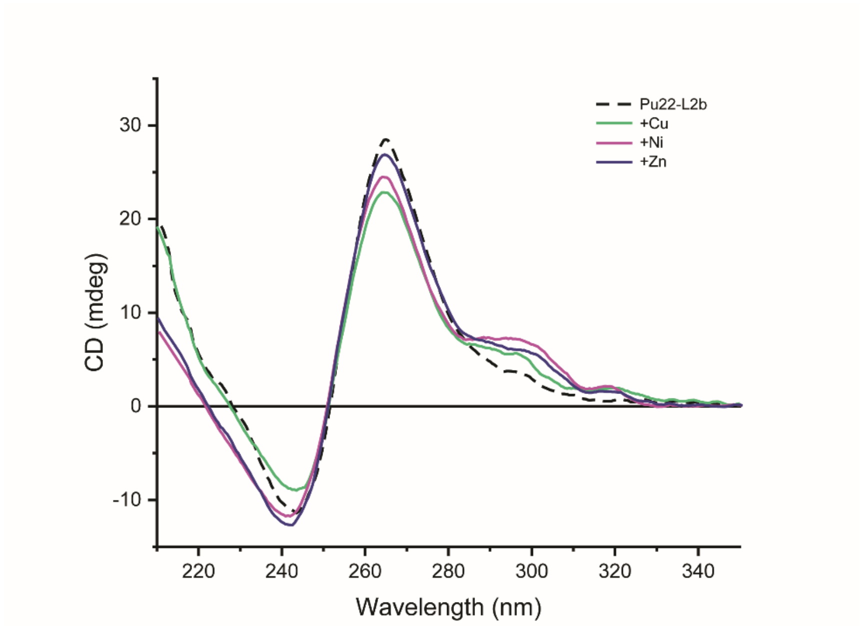

Figure S47. CD spectrum of pu22-L2a in absence or presence of various divalent metal cations. Conditions: 4μM DNA, 10mM HEPES, 100mM KCl, 4.4 μM M^{2+} (M = Cu, Ni, Zn).

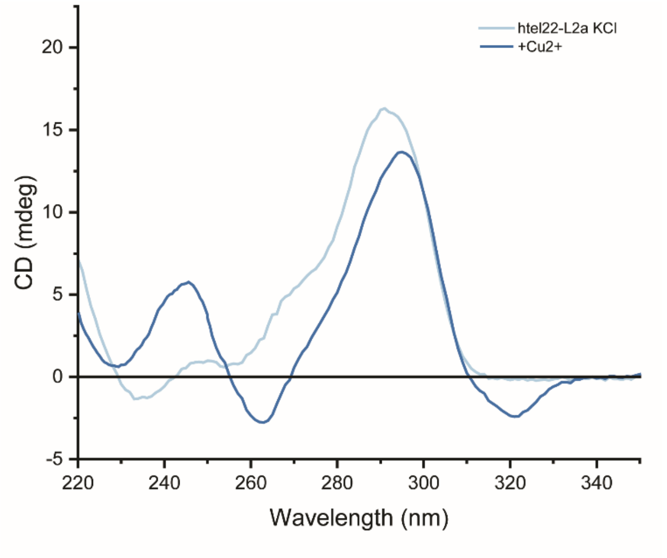

Figure S48. CD spectrum of FAM-htel22-L2a in absence or presence of Cu^{2+}. Conditions: 4μM DNA, 10mM HEPES, 100mM KCl, 4.4 μM Cu^{2+}.

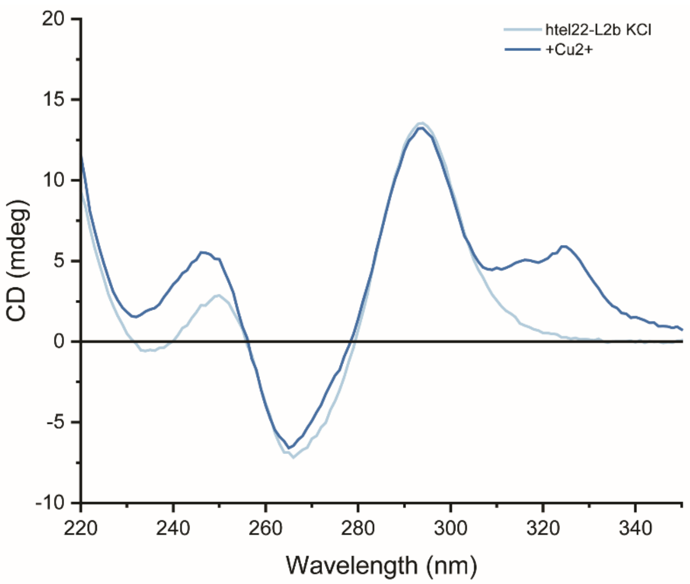

Figure S49. CD spectrum of FAM-htel22-L2b in absence or presence of Cu^{2+}. Conditions: 4μM DNA, 10mM HEPES, 100mM KCl, 4.4 μM Cu^{2+}.

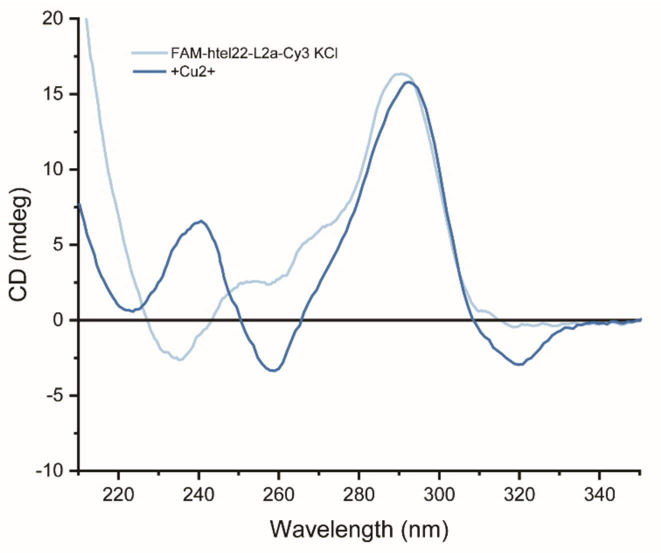

Figure S50. CD spectrum of FAM-htel22-L2a-Cy3 in absence or presence of Cu^{2+}. Conditions: 4μM DNA, 10mM HEPES, 100mM KCl, 4.4 μM Cu^{2+}.

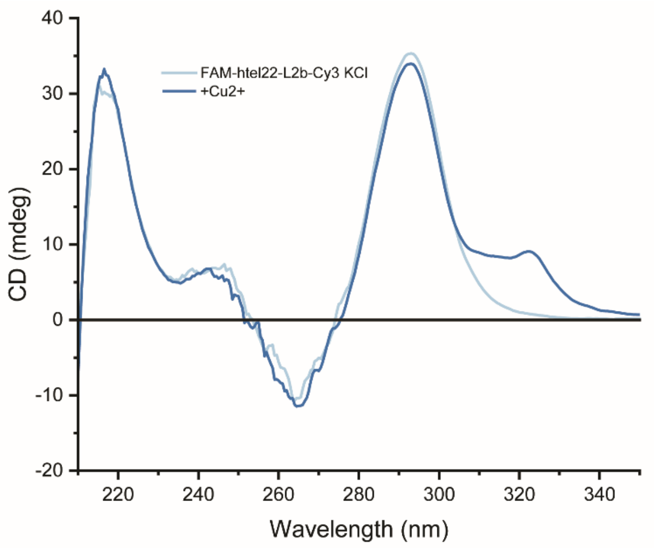

Figure S51. CD spectrum of FAM-htel22-L2b-Cy3 in absence or presence of Cu^{2+}. Conditions: 4μM DNA, 10mM HEPES, 100mM KCl, 4.4 μM Cu^{2+}.

11. Thermal Differential Spectroscopy Analysis

Thermal denaturation profiles and melting curves were recorded on a Jasco V-750 UV-Visible Spectrophotometer equipped with a PAC-743 6-cell thermostat for temperature control. The temperature was measured in the measurement cell in a water-filled cuvette. Quartz glass cuvettes (Hellma Analytics 114-QS, 1 cm path length) were used. In order to avoid condensation of water on the cuvette surface or cell window at low temperatures, a constant flow of air was pumped through the measurement cell. Evaporation of water at high temperatures and resulting changes in the absorption behaviour were minimized by a thin layer of silicon oil placed onto the sample and by tightly stoppering the cuvette. UV-VIS spectra were recorded from 350 to 220 nm with a scan rate of 200 nm/min both before (4 °C) and after thermal denaturation (95 °C). The data interval was set to 1 nm, bandwidth to 2.0 nm and the response time to 0.96 sec. All UV-VIS spectra were background corrected (cuvette, buffer and electrolyte) and zeroed using the absorption at 350 nm. To obtain the thermal difference spectra (TDS), the spectrum before denaturation (at 4 °C) was subtracted from the one after denaturation (at 95 °C).

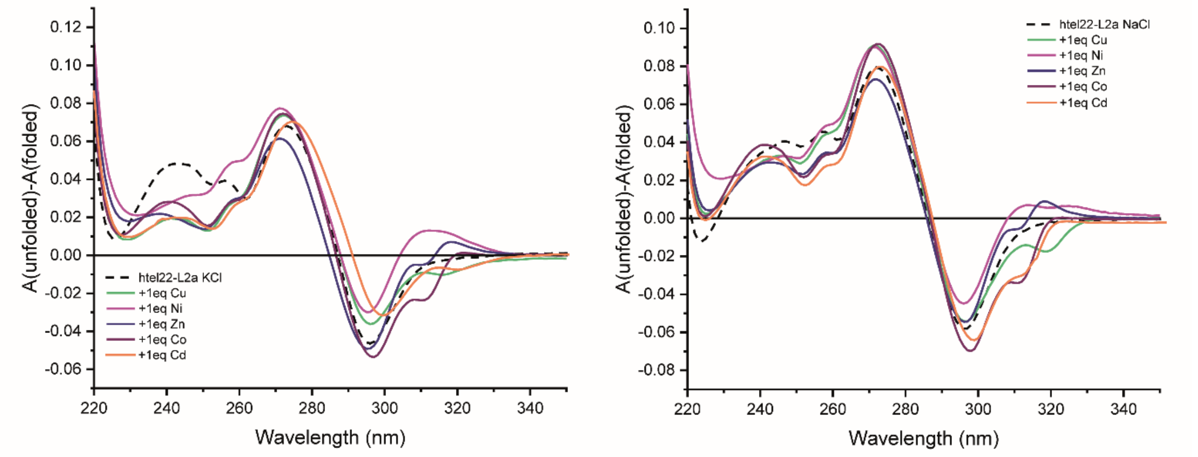

Figure S52. TDS spectra of htel22-L2a in absence or presence of various divalent metal cations. Conditions: 4µM DNA, 10mM HEPES, 100mM KCl, 4.4 µM M^{2+} (M = Cu, Ni, Zn, Co, Cd).

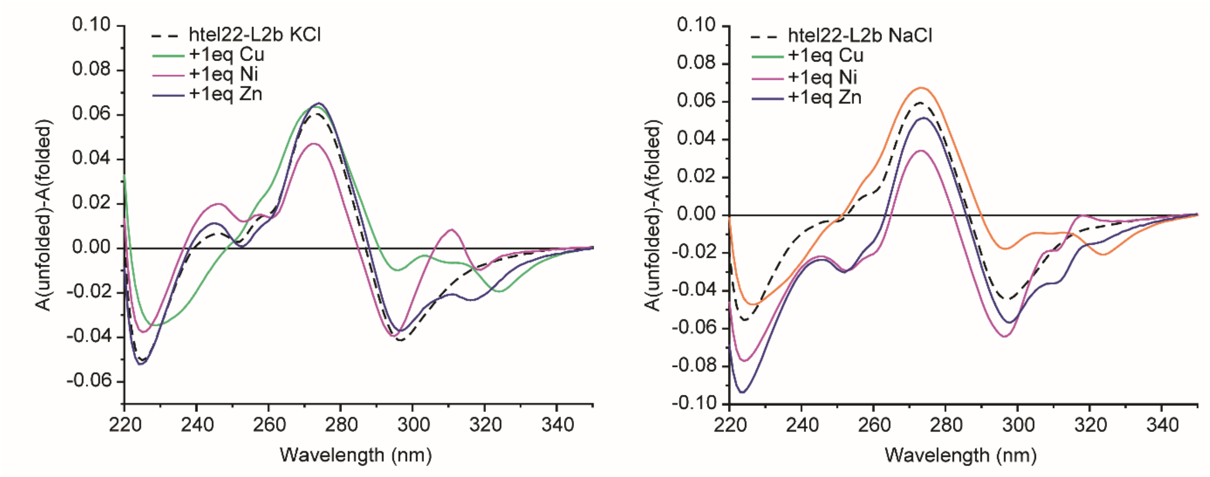

Figure S53. TDS spectra of htel22-L2b in absence or presence of various divalent metal cations. Conditions: 4μM DNA, 10mM HEPES, 100mM KCl, 4.4 μM M^{2+} (M = Cu, Ni, Zn, Co).

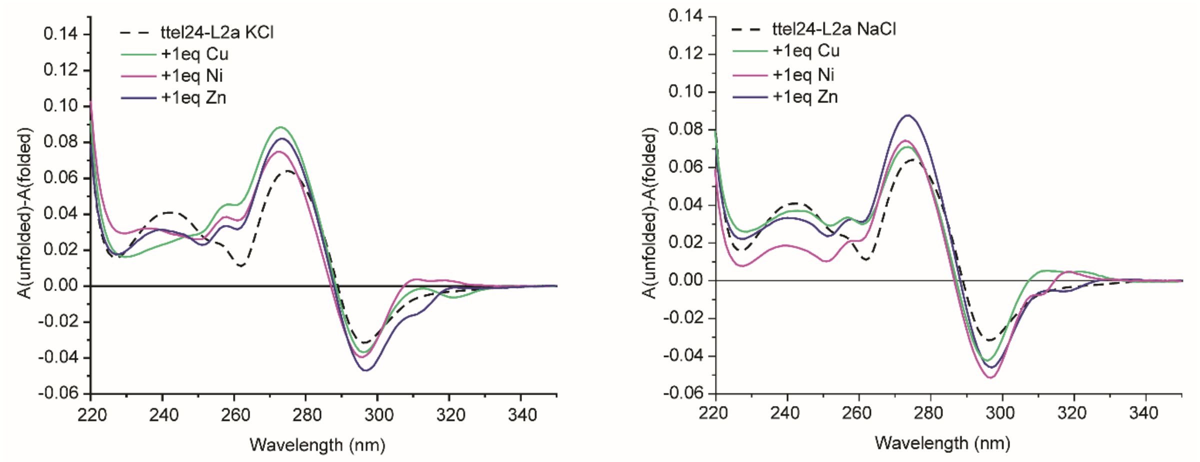

Figure S54. TDS spectra of ttel24-L2a in absence or presence of various divalent metal cations. Conditions: 4μM DNA, 10mM HEPES, 100mM KCl, 4.4 μM M^{2+} (M = Cu, Ni, Zn, Co).

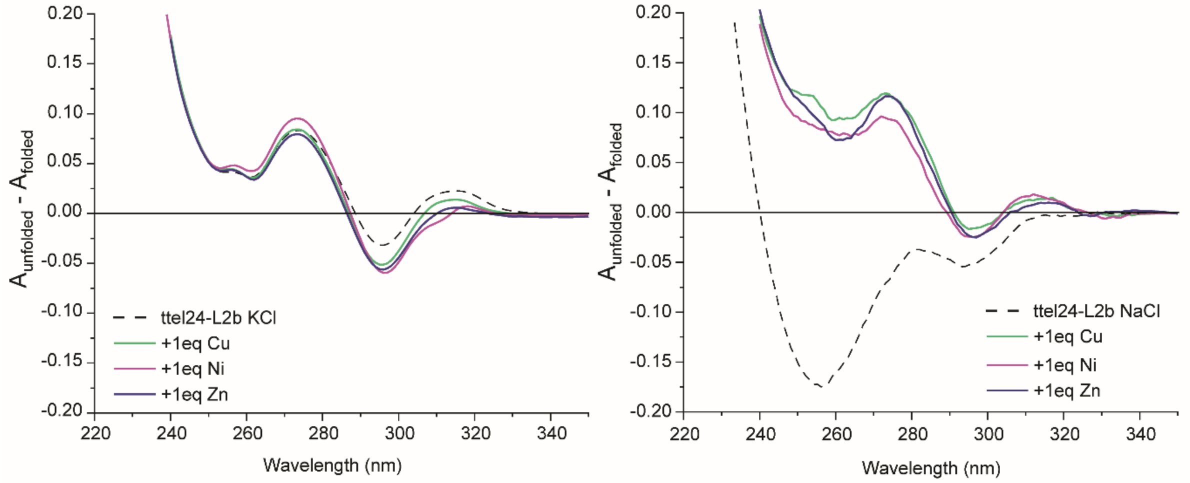

Figure S55. TDS spectra of ttel24-L2b in absence or presence of various divalent metal cations. Conditions: 4μM DNA, 10mM HEPES, 100mM KCl, 4.4 μM M2+ (M = Cu, Ni, Zn, Co).

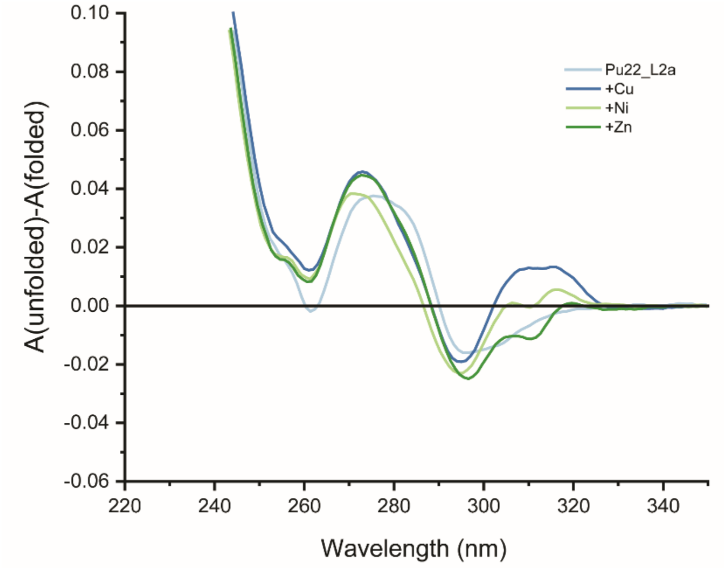

Figure S56. TDS spectrum of pu22-L2a in absence or presence of various divalent metal cations. Conditions: 4µM DNA, 10mM HEPES, 100mM KCl, 4.4 µM M^{2+} (M = Cu, Ni, Zn).

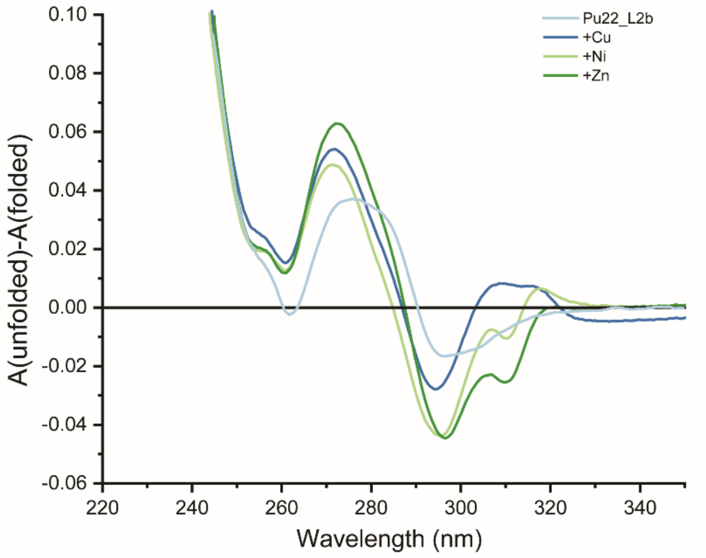

Figure S57. TDS spectrum of pu22-L2a in absence or presence of various divalent metal cations. Conditions: 4µM DNA, 10mM HEPES, 100mM KCl, 4.4 µM M^{2+} (M = Cu, Ni, Zn).

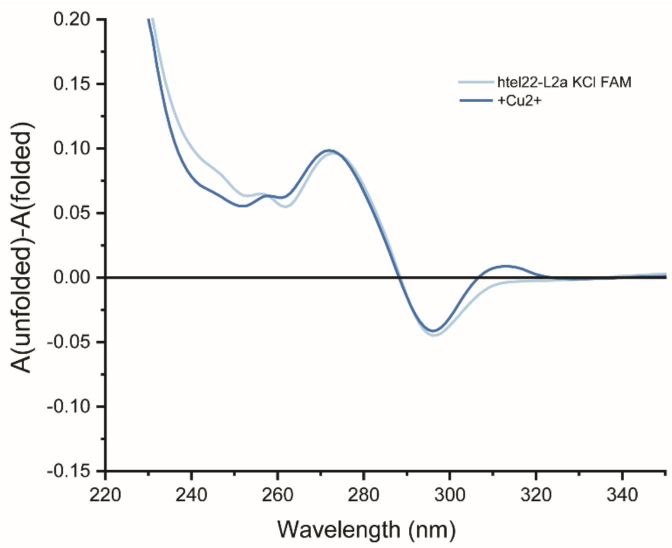

Figure S58. TDS spectrum of FAM-htel22-L2a in absence or presence of various divalent metal cations. Conditions: 4μM DNA, 10mM HEPES, 100mM KCl, 4.4 μM Cu^{2+}.

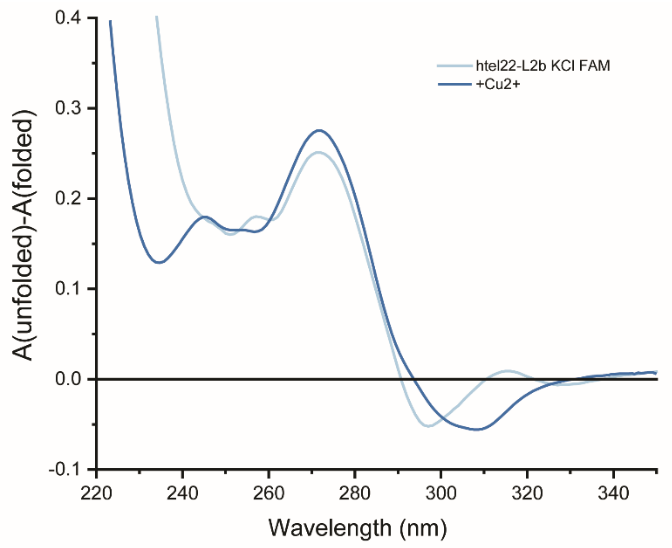

Figure S59. TDS spectrum of FAM-htel22-L2b in absence or presence of various divalent metal cations. Conditions: 4μM DNA, 10mM HEPES, 100mM KCl, 4.4 μM Cu^{2+}.

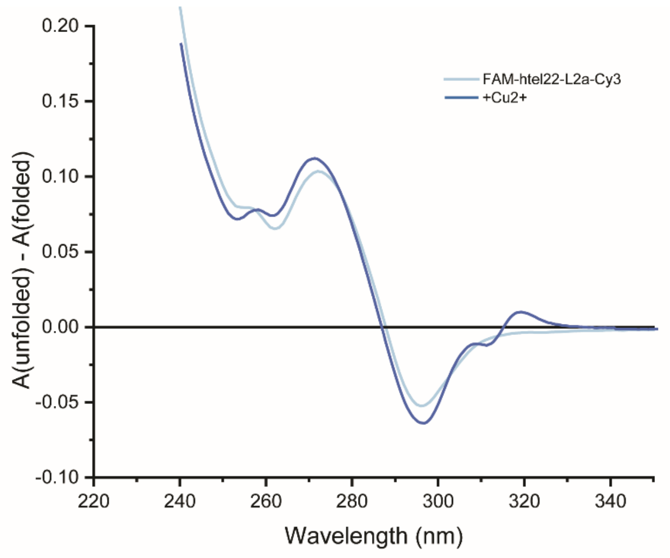

Figure S60. TDS spectrum of FAM-htel22-L2a-Cy3 in absence or presence of various divalent metal cations. Conditions: 4µM DNA, 10mM HEPES, 100mM KCl, 4.4 µM Cu^{2+}.

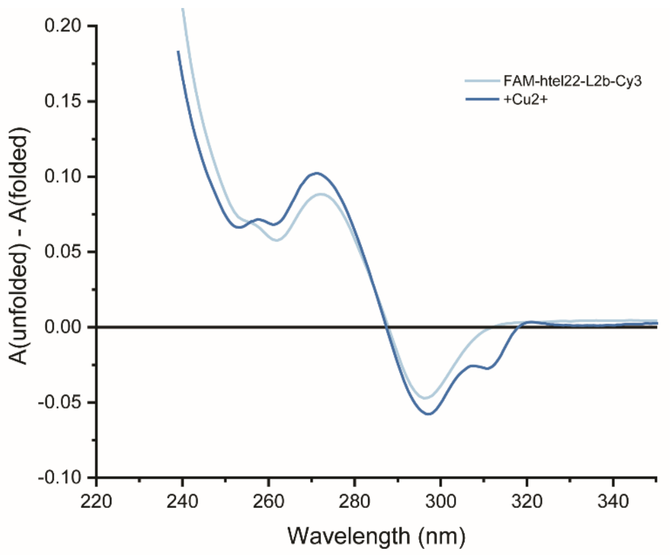

Figure S61. TDS spectrum of FAM-htel22-L2b-Cy3 in absence or presence of various divalent metal cations. Conditions: 4µM DNA, 10mM HEPES, 100mM KCl, 4.4 µM Cu^{2+}.

12. UV-Vis Melting Curve Analysis

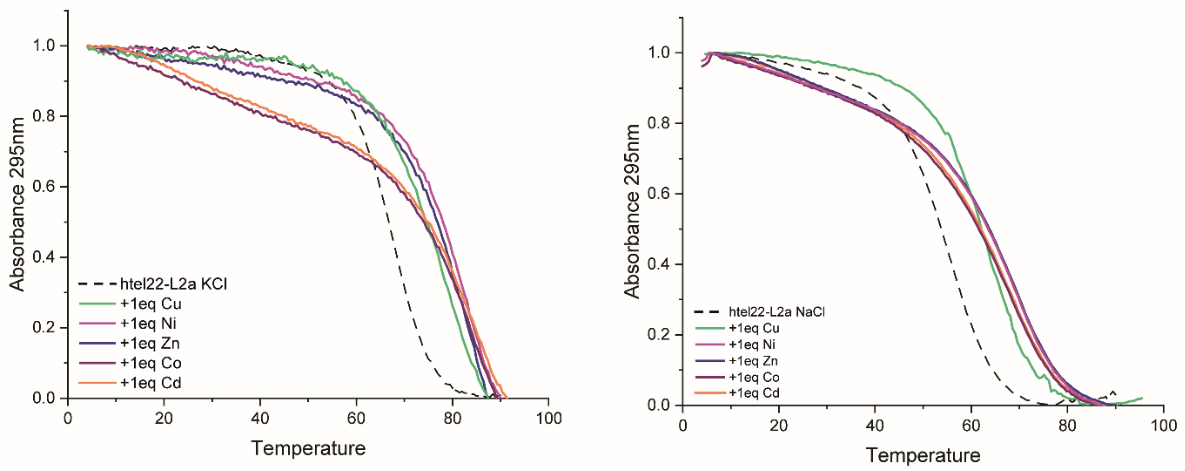

Figure S62. UV-Vis melting spectra of htel22-L2a in absence or presence of various divalent metal cations. Conditions: 4µM DNA, 10mM HEPES, 100mM KCl, 4.4 µM M^{2+} (M = Cu, Ni, Zn, Co, Cd).

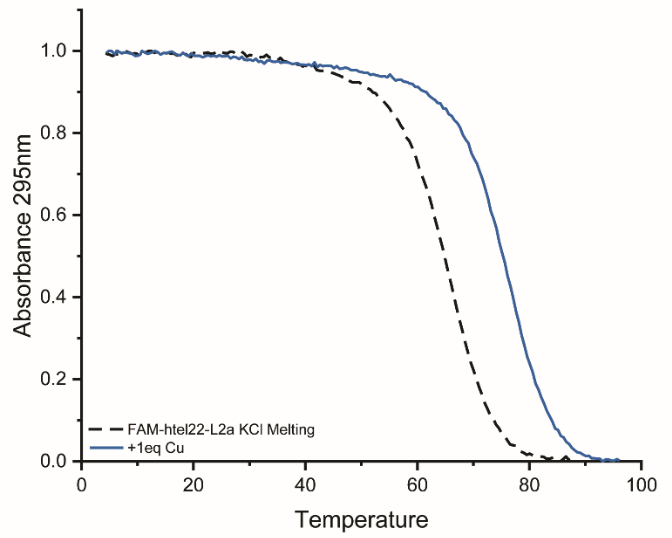

Figure S63. UV-Vis melting spectra of FAM-htel22-L2a in absence or presence of Cu^{2+}. Conditions: 4µM DNA, 10mM HEPES, 100mM KCl, 4.4 µM Cu^{2+}.

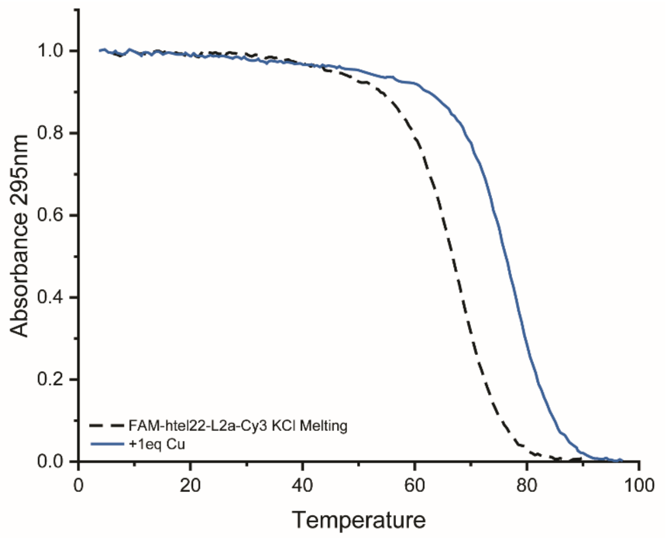

Figure S64. UV-Vis melting spectra of FAM-htel22-L2a-Cy3 in absence or presence of Cu^{2+}. Conditions: 4μM DNA, 10mM HEPES, 100mM KCl, 4.4 μM Cu^{2+}.

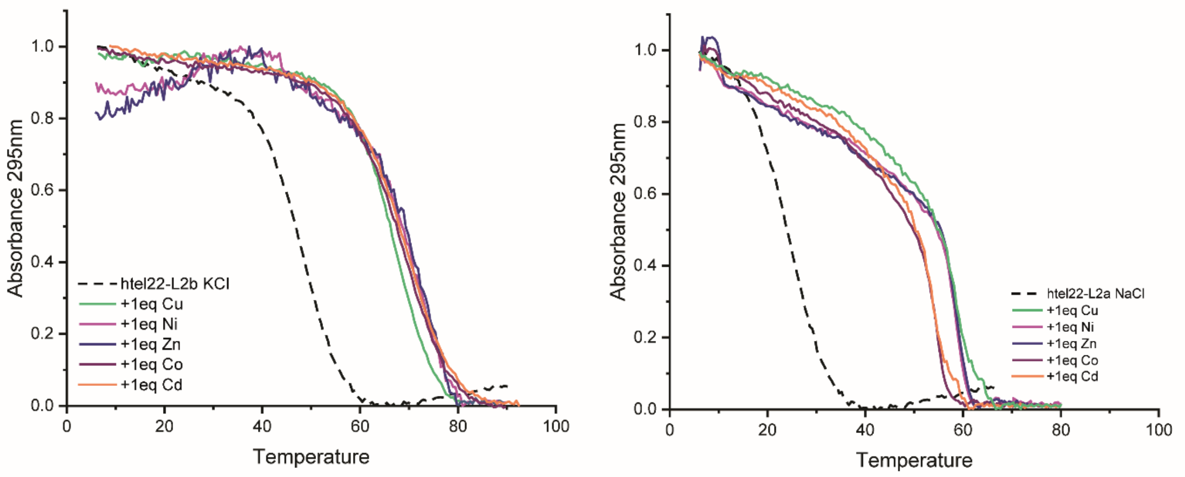

Figure S65. UV-Vis melting spectra of htel22-L2b in absence or presence of various divalent metal cations. Conditions: 4μM DNA, 10mM HEPES, 100mM KCl, 4.4 μM M^{2+} (M = Cu, Ni, Zn, Co, Cd).

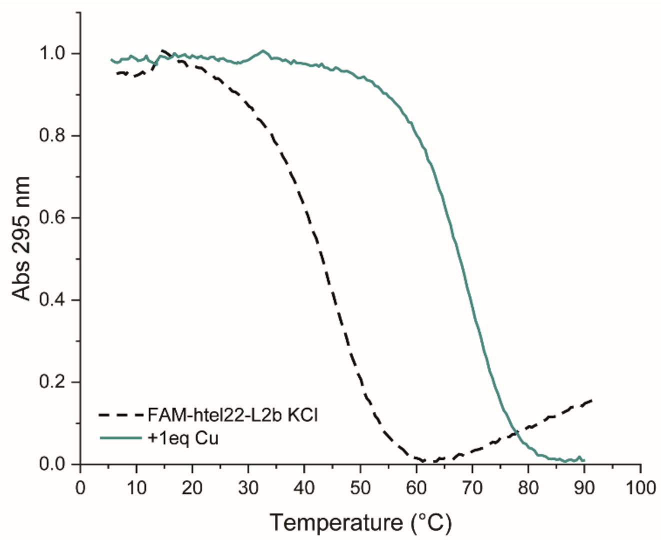

Figure S66. UV-Vis melting spectra of FAM-htel22-L2b in absence or presence of Cu^{2+}. Conditions: 4µM DNA, 10mM HEPES, 100mM KCl, 4.4 µM Cu^{2+}.

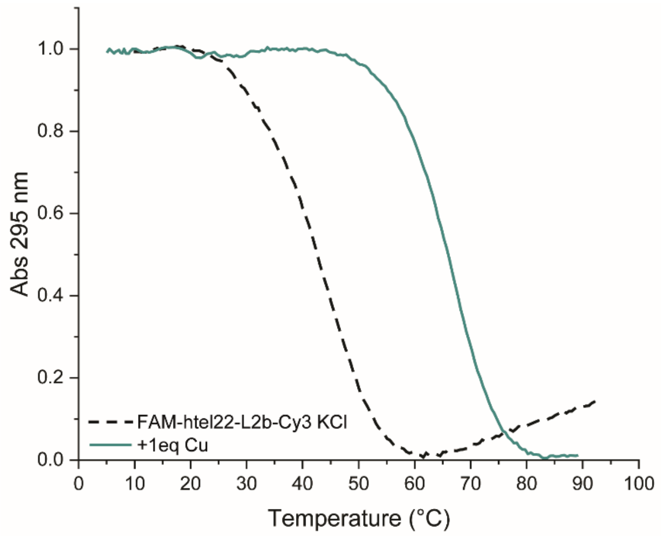

Figure S67. UV-Vis melting spectra of FAM-htel22-L2b-Cy3 in absence or presence of Cu^{2+}. Conditions: 4µM DNA, 10mM HEPES, 100mM KCl, 4.4 µM Cu^{2+}.

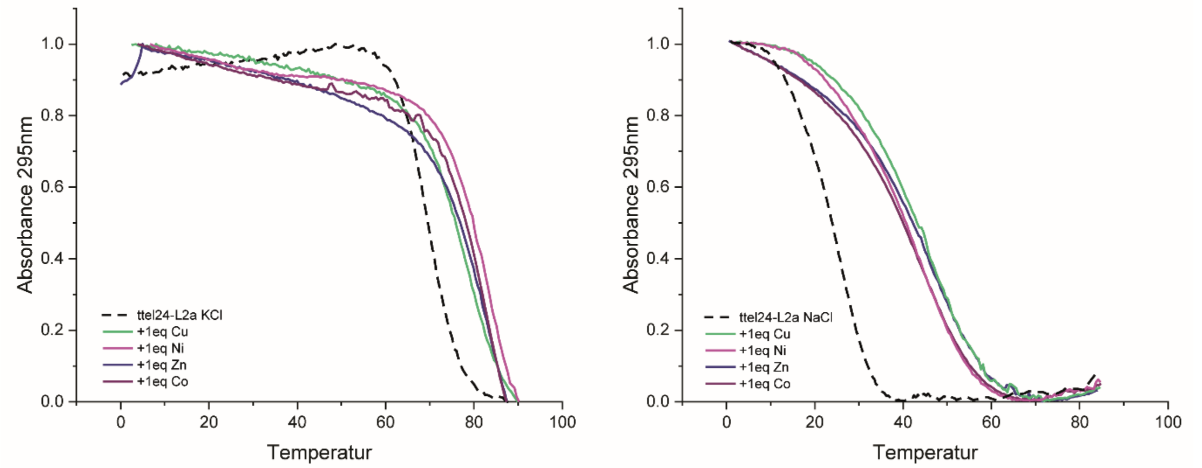

Figure S68. UV-Vis melting spectra of ttel24-L2a in absence or presence of various divalent metal cations. Conditions: 4µM DNA, 10mM HEPES, 100mM KCl, 4.4 µM M^{2+} (M = Cu, Ni, Zn, Co).

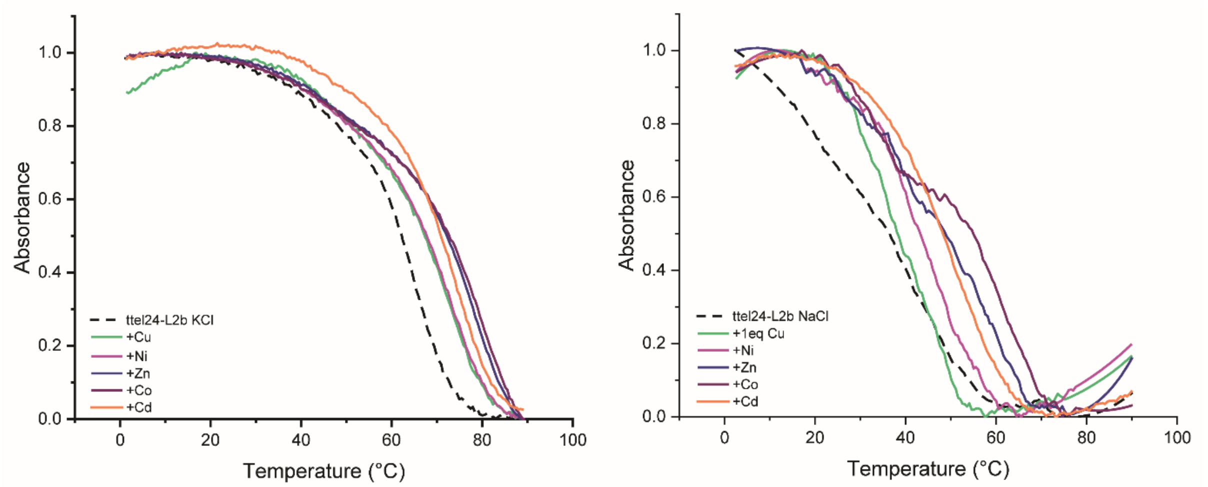

Figure S69. UV-Vis melting spectra of ttel24-L2b in absence or presence of various divalent metal cations. Conditions: 4µM DNA, 10mM HEPES, 100mM KCl, 4.4 µM M^{2+} (M = Cu, Ni, Zn, Co, Cd).

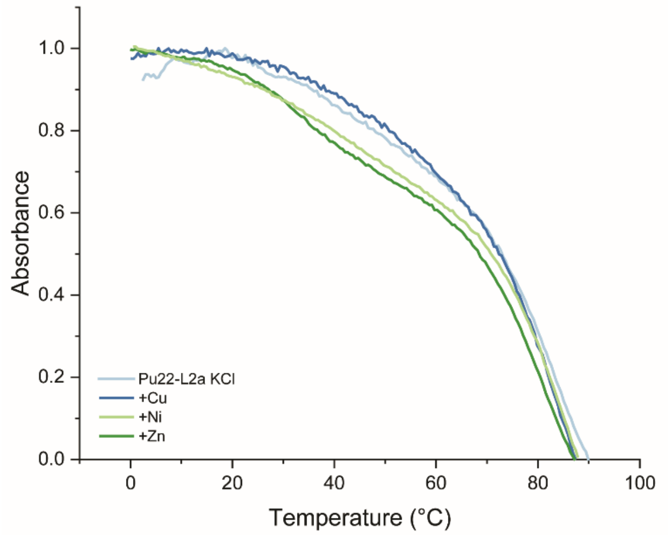

Figure S70. UV-Vis melting spectra of pu22-L2a in absence or presence of various divalent metal cations. Conditions: 4µM DNA, 10mM HEPES, 100mM KCl, 4.4 µM M^{2+} (M = Cu, Ni, Zn).

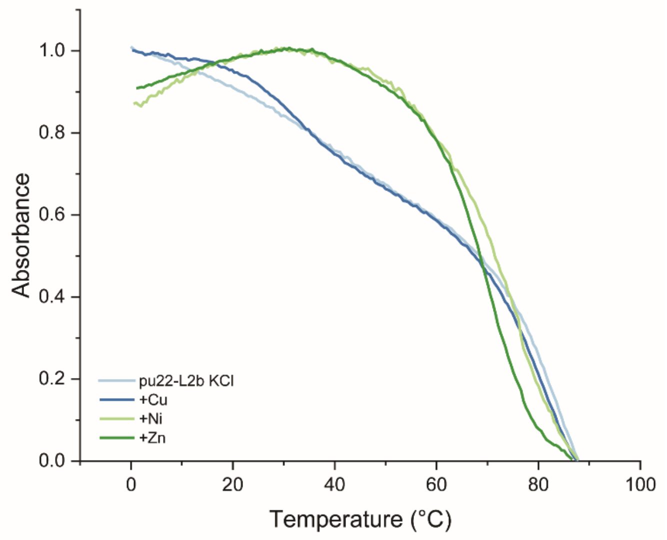

Figure S71. UV-Vis melting spectra of pu22-L2b in absence or presence of various divalent metal cations. Conditions: 4 µM DNA, 10mM HEPES, 100mM KCl, 4.4 µM M^{2+} (M = Cu, Ni, Zn).

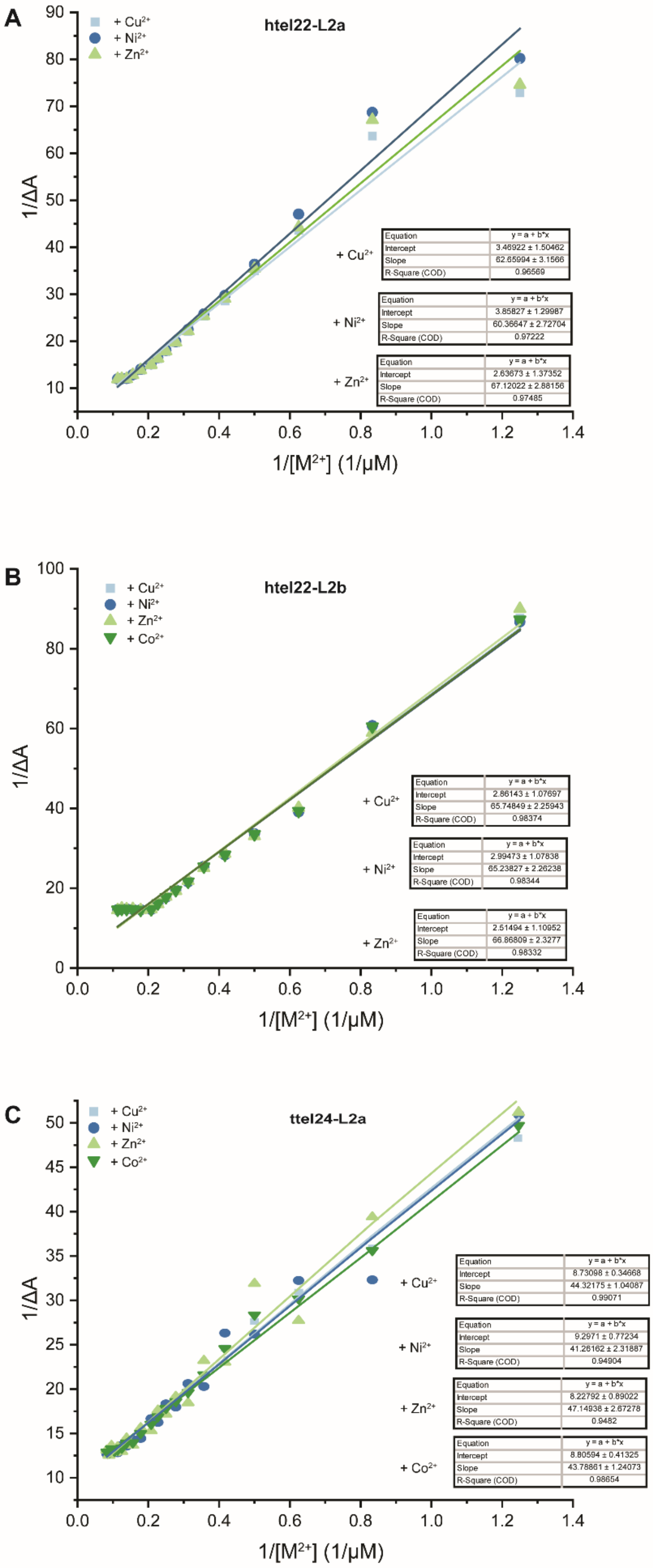

Figure S72. Benesi-Hildebrand analysis for determination of association constants *K* of BiPy-L1 modified DNA G-quadruplexes of htel22-L2a (A), htel22-L2b (B) and ttel24-L2a (C).

Table S2. The following association constants K have been obtained from Benesi-Hildebrand:

Name	Metal	Association constant (M^{-1})
htel22-L2a	Cu^{2+}	$5.53 * 10^{10} \pm 2.41 * 10^{9}$
htel22-L2a	Ni^{2+}	$6.38 * 10^{10} \pm 2.16 * 10^{9}$
htel22-L2a	Zn^{2+}	$3.92 * 10^{10} \pm 2.05 * 10^{9}$
htel22-L2b	Cu^{2+}	$4.35 * 10^{10} \pm 1.64 * 10^{9}$
htel22-L2b	Ni^{2+}	$4.59 * 10^{10} \pm 1.65 * 10^{9}$
htel22-L2b	Zn^{2+}	$3.75 * 10^{10} \pm 1.65 * 10^{9}$
ttel24-L2a	Cu^{2+}	$1.96 * 10^{11} \pm 0.89 * 10^{10}$
ttel24-L2a	Ni^{2+}	$2.25 * 10^{11} \pm 2.22 * 10^{10}$
ttel24-L2a	Zn^{2+}	$1.74 * 10^{11} \pm 2.12 * 10^{10}$
ttel24-L2a	Co^{2+}	$2.01 * 10^{11} \pm 1.10 * 10^{10}$

13. Molecular Dynamics Simulation

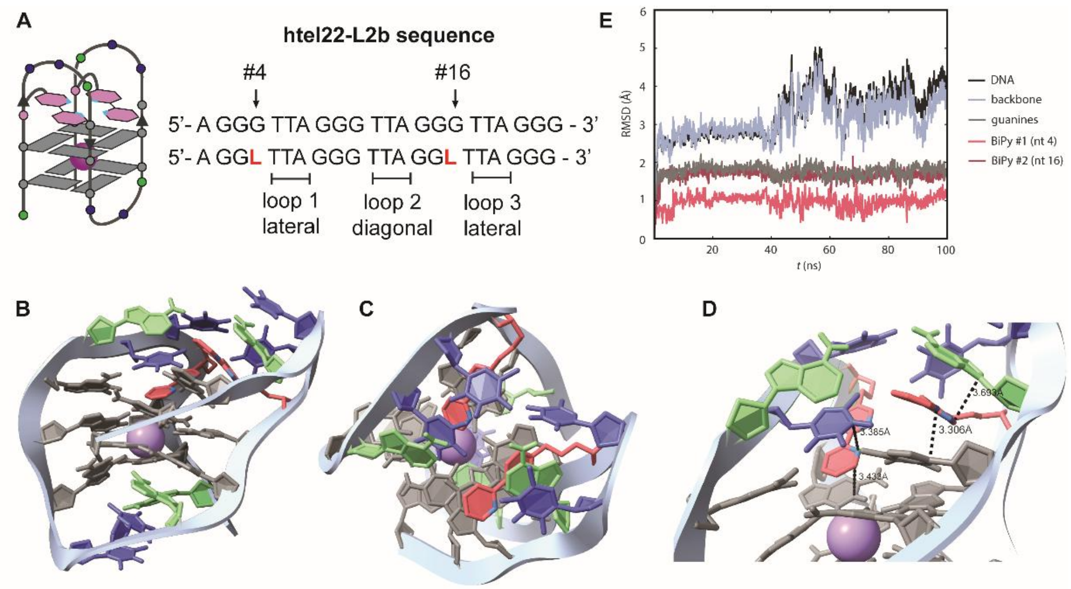

Figure S73. Molecular dynamics simulation of **htel22-L2b** in absence of any divalent metal cation M^{2+}. Starting structure used for modelling was PDB: 143D. Guanines coloured in grey, thymines in blue, adenines in green, bipyridine modifications in pink, potassium cation in violet. A) Oligonucleotide sequence shown with placement of the bipyridine ligand modifications (BiPy) marked with L. B) Side-view of the representative structure from the 3'-end. C) Top-view of the representative structure. D) Zoomed view of the loop region with distances between selected nucleotides indicating possible ranges of pi-stacking interactions. E)

Comparison of RMSD values of individual components of the models for **htel22-L2b** in absence of Cu^{2+} stabilisation.

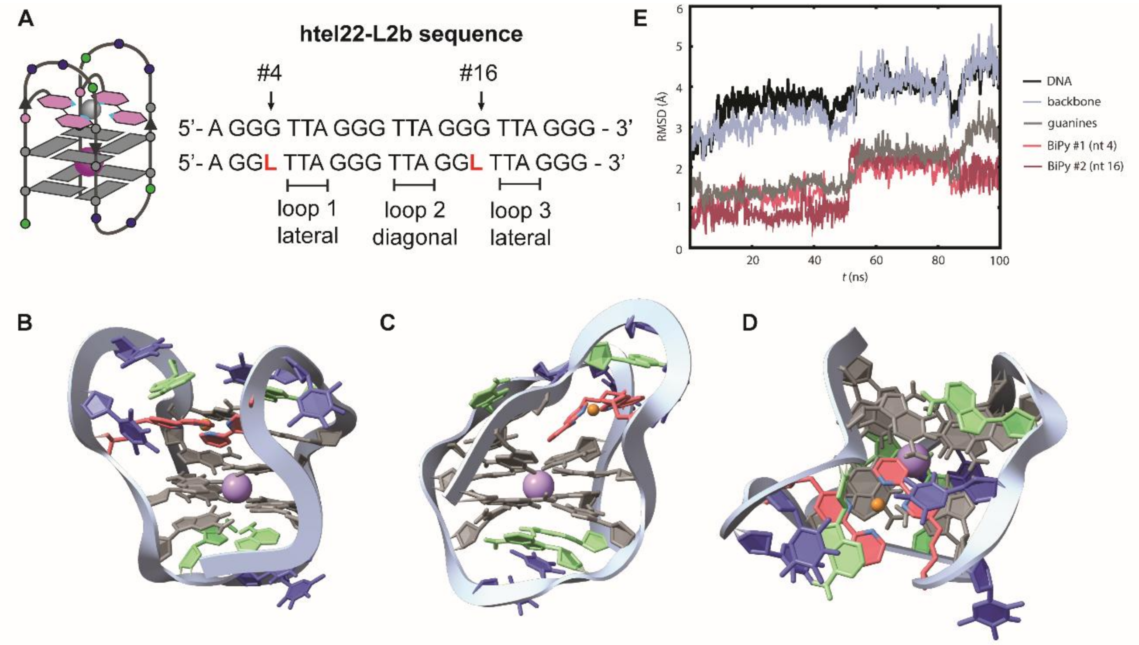

Figure S74. Molecular dynamics simulation of **htel22-L2b** in presence of Cu^{2+}. Starting structure used for modelling was PDB: 143D. Guanines coloured in grey, thymines in blue, adenines in green, bipyridine modifications in pink, potassium cation in violet. A) Oligonucleotide sequence shown with placement of the bipyridine ligand modifications (BiPy) marked with L. B) Side-view of the representative structure from the 5'-end. C) Side-view of the representative structure from the 3'-end. D) Top view of the representative structure showcasing the placement of the bipyridine metal-complex. E) Comparison of RMSD values of individual components of the models for **htel22-L2b** in presence of Cu^{2+} stabilisation.

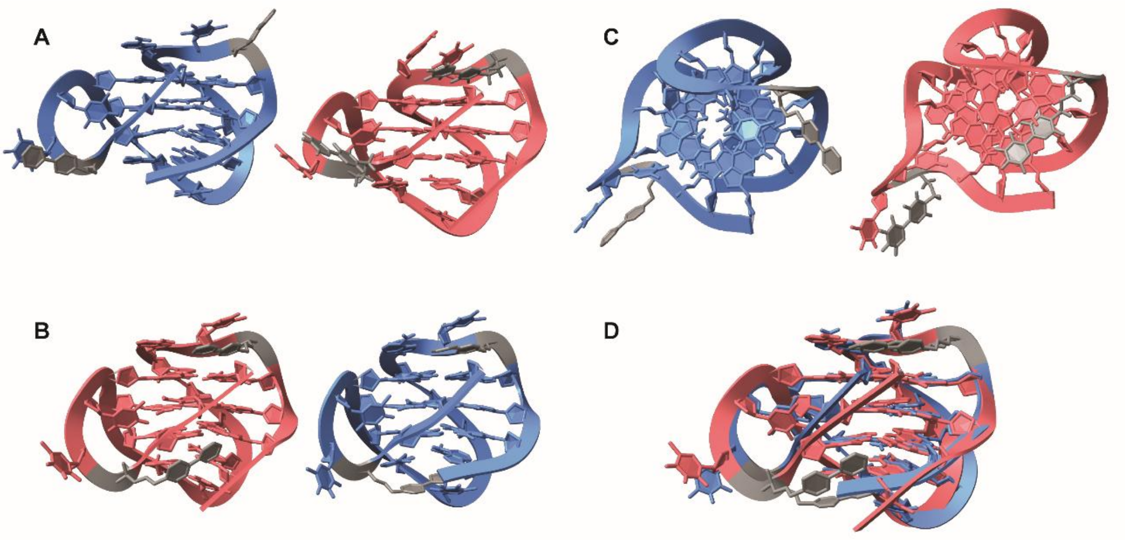

Figure S75. Molecular dynamics simulation results of htel22-L2a where orientation of the bipyridine units was altered after full completion of the simulation run and a secondary rerun was performed to check for arbitrary positioning of the bipyridine units. A) Side view from 5'-end of starting structure with manually altered position of bipyridine modification at nt position #5 (blue) resulting structure after rerunning simulation run over 50 ns (red). B) Top view of starting structure with manually altered position of bipyridine modification at nt position #5 (blue) resulting structure after rerunning simulation run over 50 ns (red). C) Side view from 5'-

end of starting structure with manually altered position of bipyridine modification at nt position #20 (blue) resulting structure after rerunning simulation run over 50 ns (red). D) Overlay of starting structure with manually altered position of bipyridine modifications and resulting structure after rerunning simulation run over 50 ns (red).

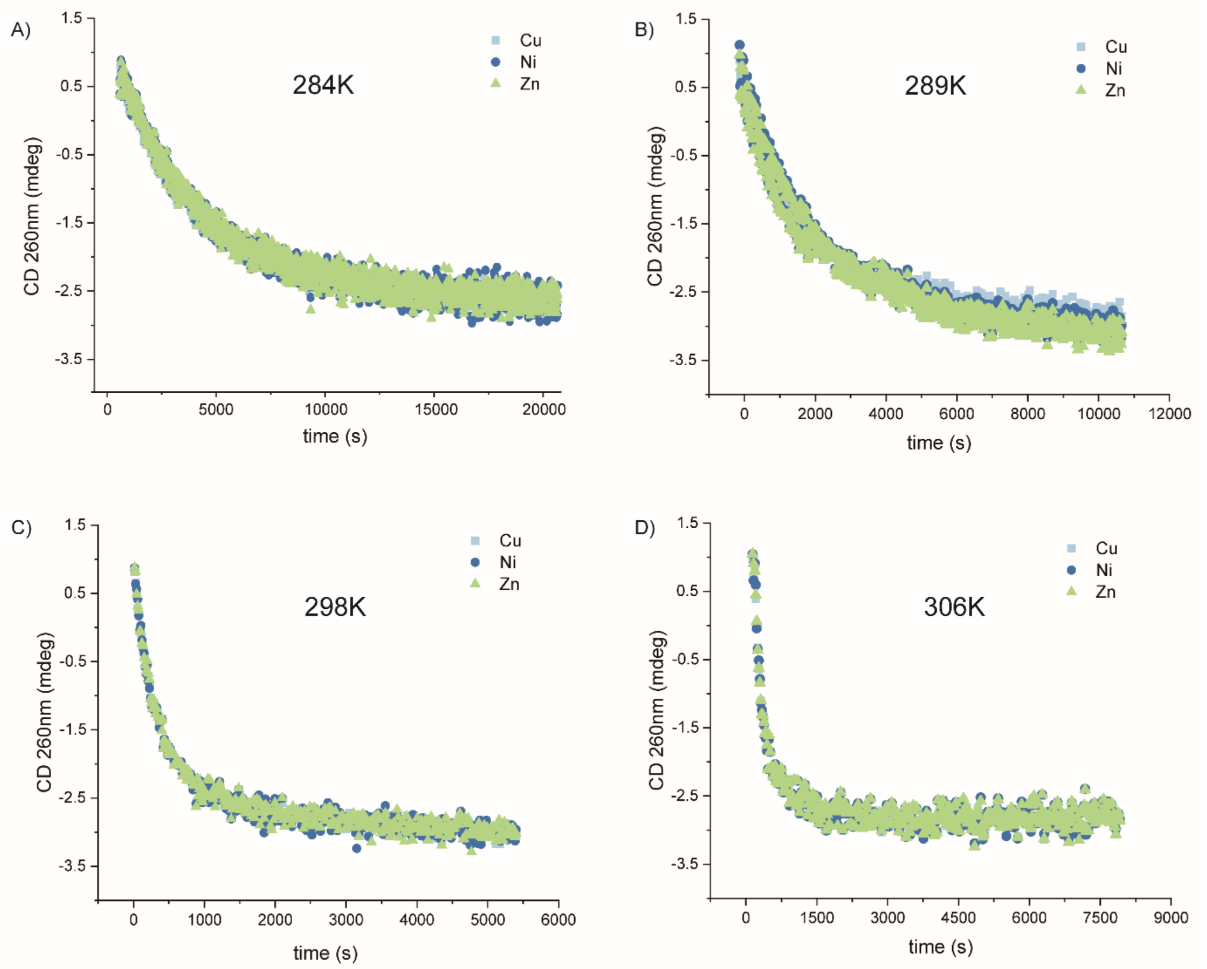

Figure S76. Decay of the CD signal of **htel22-L2a** at 260 nm at different temperature A) 284K, B) 289K, C) 298K and D) 306K measured in intervals of 10 s after addition of 1.1eq of the corresponding divalent metal sulfate MSO_4 (M = Cu, Ni, Zn).

Table S3. Calculated kinetic rate constants (k_{obs}) for first-order reaction kinetics of **htel22-L2a** refolding from hybrid to antiparallel with different metals (Cu^{2+}, Ni^{2+} and Zn^{2+}) at different temperatures.

Temperature [K]	k_{obs} Cu^{2+} [s^{-1}]	k_{obs} Ni^{2+} [s^{-1}]	k_{obs} Zn^{2+} [s^{-1}]
284	1.14 * 10^{-4}	1.11 * 10^{-4}	1.13 * 10^{-4}
289	2.50 * 10^{-4}	2.19 * 10^{-4}	2.04 * 10^{-4}
298	9.09 * 10^{-4}	9.05 * 10^{-4}	9.16 * 10^{-4}
306	1.73 * 10^{-3}	1.76 * 10^{-3}	1.75 * 10^{-4}

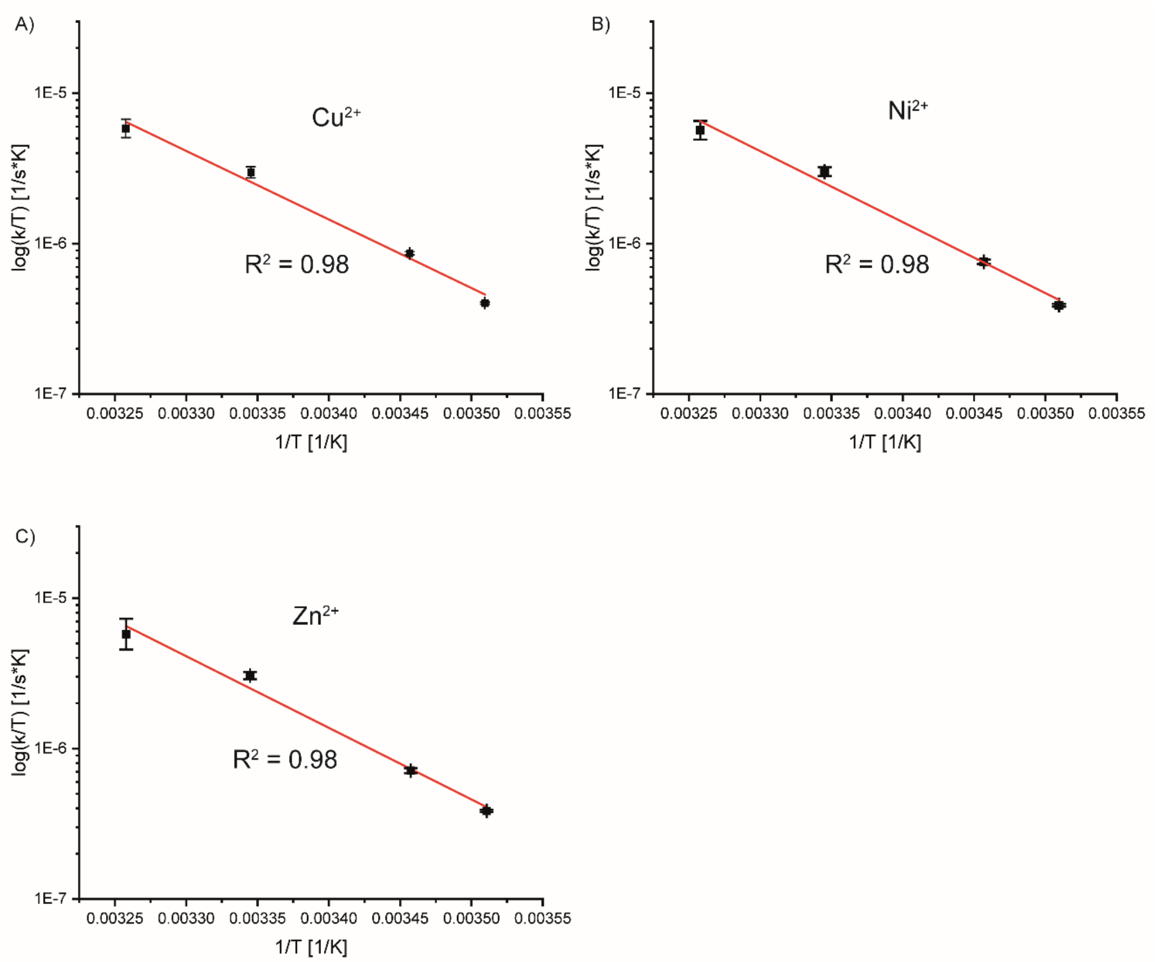

Figure S77. Eyring-plot analysis for the determination of enthalpy ($\Delta H^‡$) and entropy ($\Delta S^‡$) of activation and Gibbs activation energy ($\Delta G^‡$ or E_a) with A) Cu^{2+}, B) Ni^{2+} and C) Zn^{2+}.

Table S4. Calculated enthalpy ($\Delta H^‡$) and entropy ($\Delta S^‡$) of activation and Gibbs activation energy ($\Delta G^‡$ or E_a) at 298.15K.

**Metal**	**$\Delta H^‡$ [kJ/mol]**	**$\Delta S^‡$ [J/mol*K]**	**$\Delta G^‡$ [kJ/mol]**
**Cu^{2+}**	37.90 ± 3.56	80.31 ± 12.06	61.84 ± 5.06
**Ni^{2+}**	39.26 ± 2.22	84.80 ± 10.89	64.54 ± 4.57
**Zn^{2+}**	39.59 ± 3.35	85.88 ± 11.36	65.19 ± 4.76

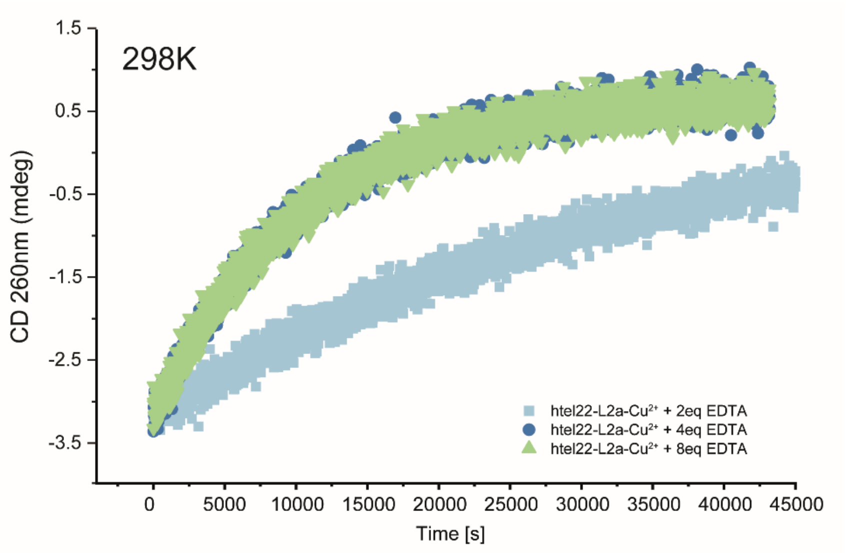

Figure S78. Change of the CD signal of **htel22-L2a** with complexed Cu^{2+}at 260 nm indicating topological change from antiparallel to hybrid at 298K with varying amounts of EDTA. The different trace of the light blue curve may indicate that two eq. of EDTA may not be sufficient to fully transform all metalated DNA, most likely due to competition with traces of other metals that are sequestered by part of the added EDTA. Addition of 4 or 8 eq. of EDTA then apparently makes no difference for the rate of the structural transformation, as the decomplexation is rather fast (see below) and thus not the rate determining step (the refolding is the much slower process).

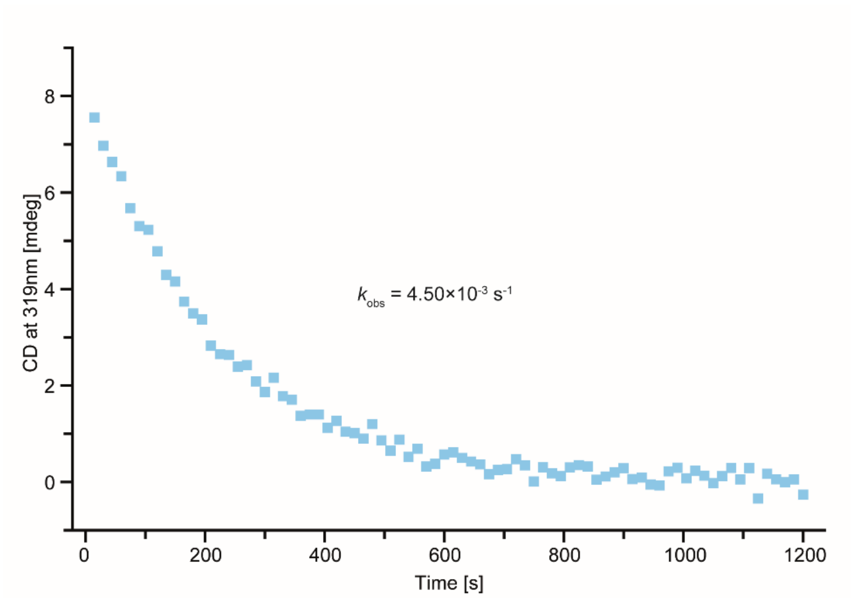

Figure S79. Decay of the CD signal of **htel22-L2b** measured at 319 nm which is a characteristic CD signature of the bipyridine-Cu^{2+} complex under chiral induction of the surrounding DNA (298K). The observed k_{obs} = 4.50 * 10^{-3} s^{-1} is significantly faster than the observed kinetic rate constants for the G-quadruplex refolding after EDTA removal, indicating a faster demetallation process followed by a slower DNA refolding process.

14. PEG200 experiments

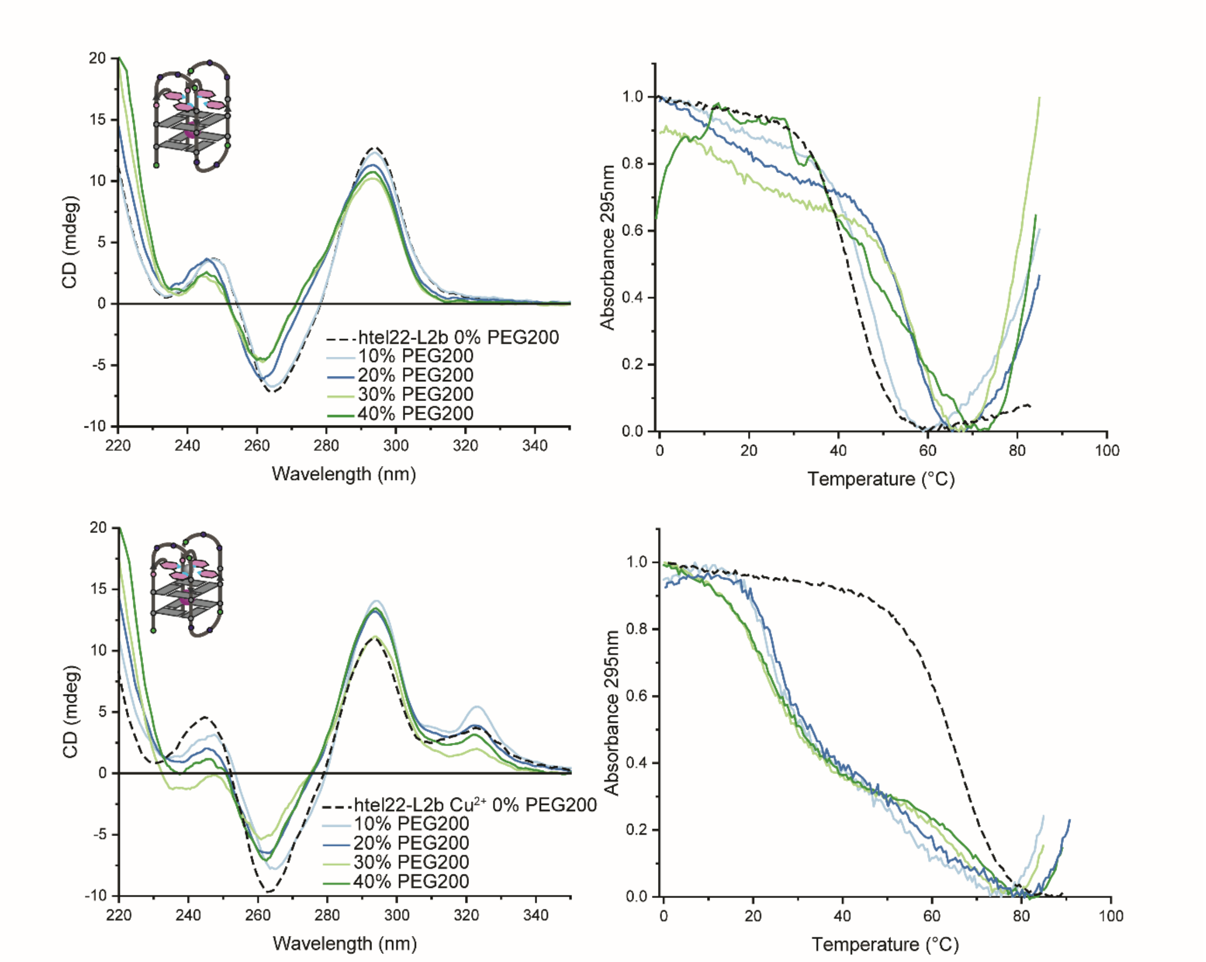

Figure S80. CD spectra and UV-Vis melting spectra of htel22-L2b in absence and presence of Cu^{2+} and increasing amount (vol%) of PEG200.

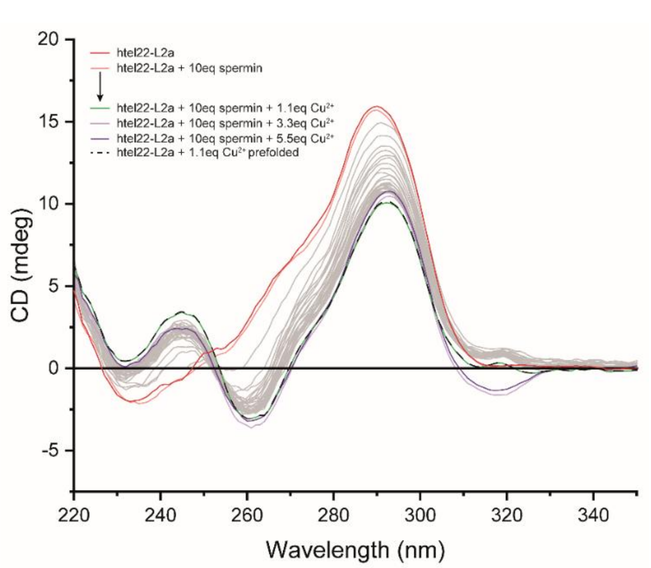

Figure S81. CD spectrum of **htel22-L2a** showcasing the slow refolding from hybrid 3+1 (red line) to antiparallel topology (green line) upon addition of $CuSO_4$ in presence of 10 eq. of spermin (grey lines, 5min intervals). Additional spectra with 3.3 eq. and 5.5 eq. $CuSO_4$ (light and dark violet respectively) show no additional changes or refolding. Comparison to prefolded **htel22-L2a** with 1.1eq $CuSO_4$ (dotted line).

15. Cellular studies in HeLa and U2OS cells

HeLa and U2OS cells were grown in high glucose Dulbecco's modified Eagle medium (DMEM) containing 10% fetal bovine serum (FBS) at 37 °C with 5% CO_2 in humidified air. For viability experiments, cells were seeded at a density of 10,000 cells per well in Greiner-Bio black µClear plates which had been coated in poly-D-lysine.

MTS Assay

To measure cellular toxicity of the probe, MTS assays were conducted with a Assay kit reagents mix consisting of a MTS solution in MilliQ water at 2 mg/mL and a phenazine methosulfate (PMS) solution in MiliQ water at 0.92 mg/mL which were mixed in a 2:0.1 ration (MTS:PMS). This solution was then mixed with DMEM containing 10% FBS in a 1:4 ratio (MTS/PMS mix:medium). HeLa and U2OS cells were grown in high glucose Dulbecco's modified Eagle medium (DMEM) containing 10% fetal bovine serum (FBS) at 37 °C with 5% CO_2 in humidified air at 5000 cells per well in a 96-well plate over 16h after which media was replaced with fresh media and incubated with the respective oligonucleotide compound and again incubated under identical conditions for 16h. Lastly media was removed and replaced with MTS reagent/buffer mixture and incubated for 4h.

Absorbance of the MTS reagent mix was then measured at 490 nm from which absorbance at 630 nm was subtracted as background correction. The experiment was conducted in triplicate. Positive controls were conducted with DMSO to ensure no dehydrogenase activity. Negative controls were conducted with cells in just medium to ensure full dehydrogenase activity. The respective absorbance maximum and minimum absorbance values were then normalised to the negative and positive controls.

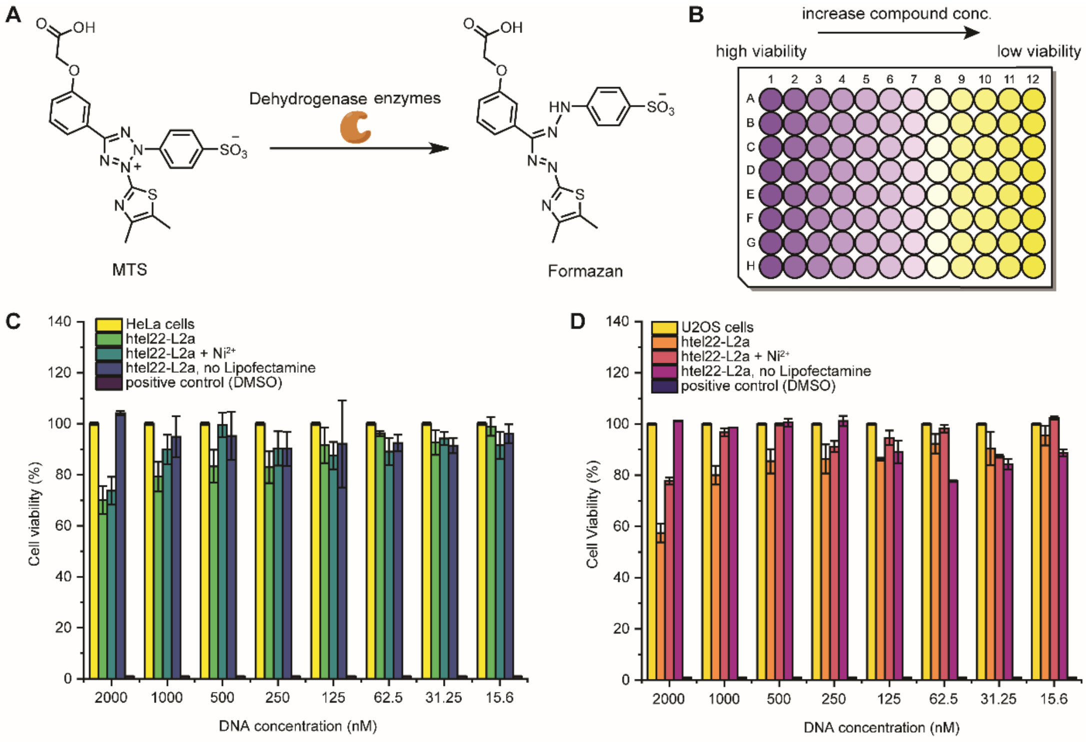

Figure S82. MTS Assay of FAM-htel22-L2a in absence or presence of Ni^{2+} and in absence or presence of Lipofectamine2000. Negative control: 10000 cells per well in growth medium. Positive control: Incubation in medium with 50% DMSO over 16h at 37°C and 5% CO_2.

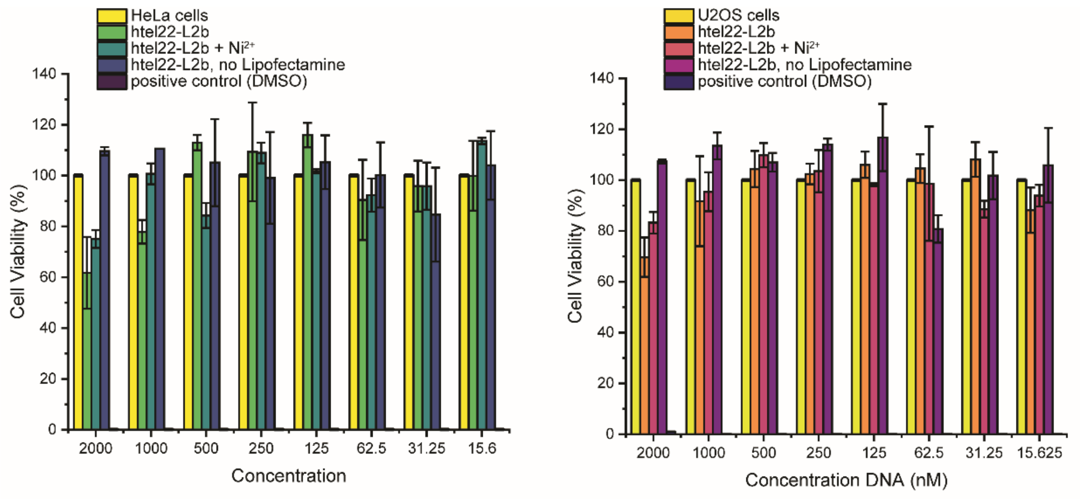

Figure S83. MTS Assay of FAM-htel22-L2b in absence or presence of Ni^{2+} and in absence or presence of Lipofectamine2000. Negative control: 10000 cells per well in growth medium. Positive control: Incubation in medium with 50% DMSO over 16h at 37°C and 5% CO_2.

Transfection Assay

DNA transfection was performed with lipofectamine 2000 (Invitrogen) following the manufacturer's instructions: G4 DNA was incubated with lipofectamine at a 3:1 ratio of lipofectamine:DNA in DMEM (Gibco) for 15 min. The lipofectamine/DNA mixture was then incubated with cells for up to 24 hours and imaged every 4h using a Sartorius Incucyte S3 Live-Cell Analysis System from which total green count over all cells within each well was plotted over time to determine the optimal incubation time after which no more signal enhancement was present. All cell imaging experiments are an average of at least three independent biological repeats.

Fluorescence Microscopy

Confocal images of single fluorescently labelled G-quadruplex strands (FAM label) were acquired using a Leica SP5 II confocal microscope after incubation of U2OS/HeLa cells with the DNA samples for 16 hours. Images were taken with a 100× oil immersion objective (correction collar, NA = 1.2, Leica) after excitation using an internal Ar+ laser at 495 nm and detection at 500-700 nm.

For FRET based experimets, confocal images of U2OS and HeLa cells in 8 well chamber slides were acquired using a Leica Stellaris 8 inverted microscope equipped with a diode laser (405 nm) and a white light laser (440-790 nm). Images were collected using a 63x oil immersion objective (correction collar, NA = 1.4, Leica) at a resolution of 512 x 512 pixels. Samples were excited using the white light laser at 495 nm, and emission detected 505-650 nm, using Airy pinhole 1.

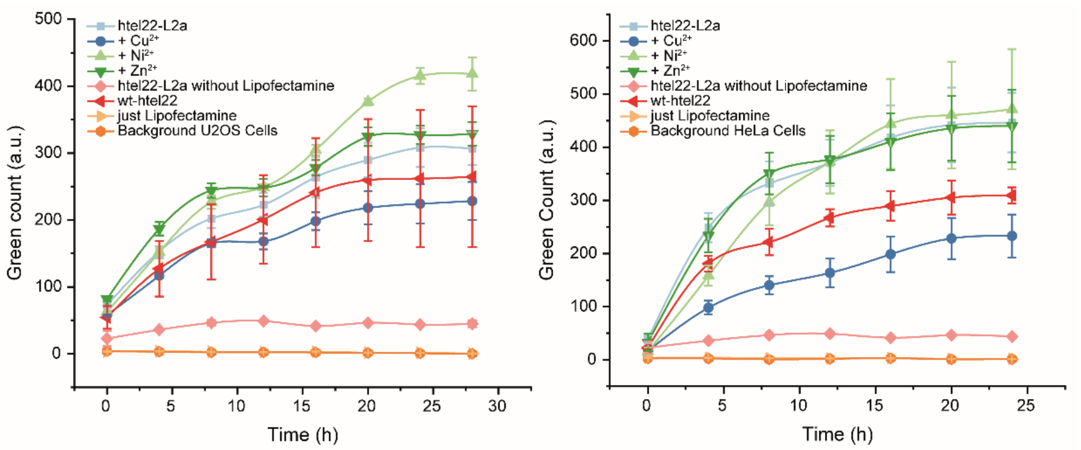

Figure S84. Green fluorescence count ($\lambda_{ex.}$ = 490nm, $\lambda_{em.}$ = 520nm) of the cells transfected with htel22-L2a in absence and presence of M^{2+} (M = Cu, Ni, Zn) and in absence and presence of Lipofectamine2000.

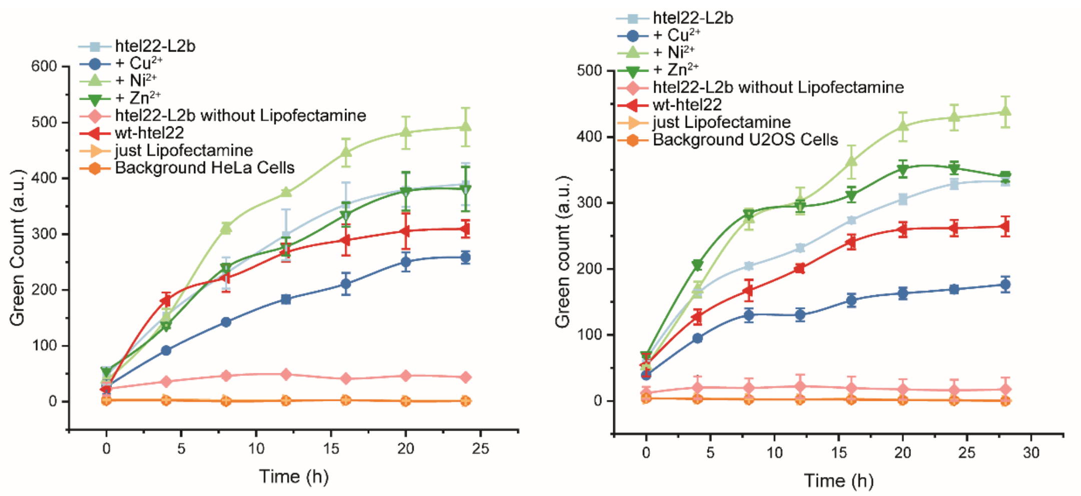

Figure S85. Green fluorescence count ($\lambda_{ex.}$ = 490nm, $\lambda_{em.}$ = 520nm) of the cells transfected with htel22-L2b in absence and presence of M^{2+} (M = Cu, Ni, Zn) and in absence and presence of Lipofectamine2000.

16. Fluorescence Spectroscopy Analysis

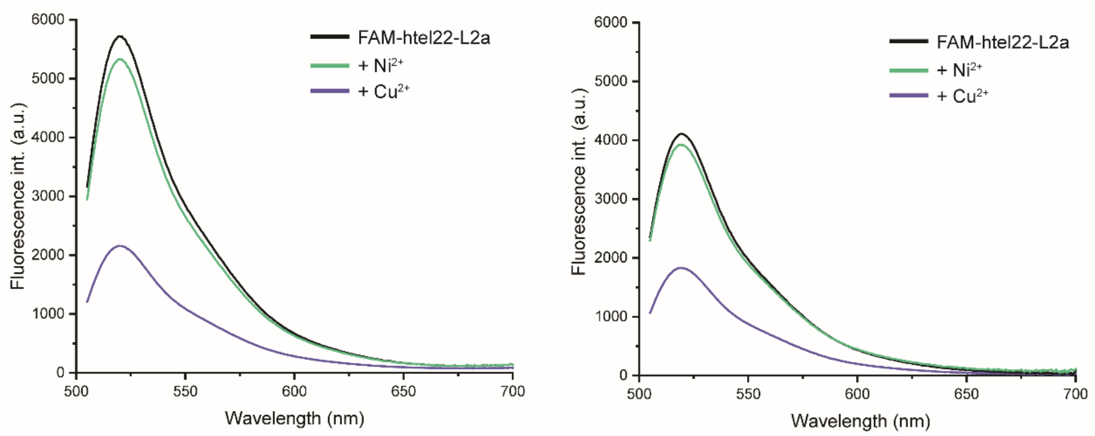

Figure S86. Fluorescence spectra of FAM-htel22-L2a and FAM-htel22-L2b in absence and presence of either Ni^{2+} and Cu^{2+} showcasing metal dependent fluorescence quenching of the modified G-quadruplexes ($\lambda_{ex.}$ = 490nm).

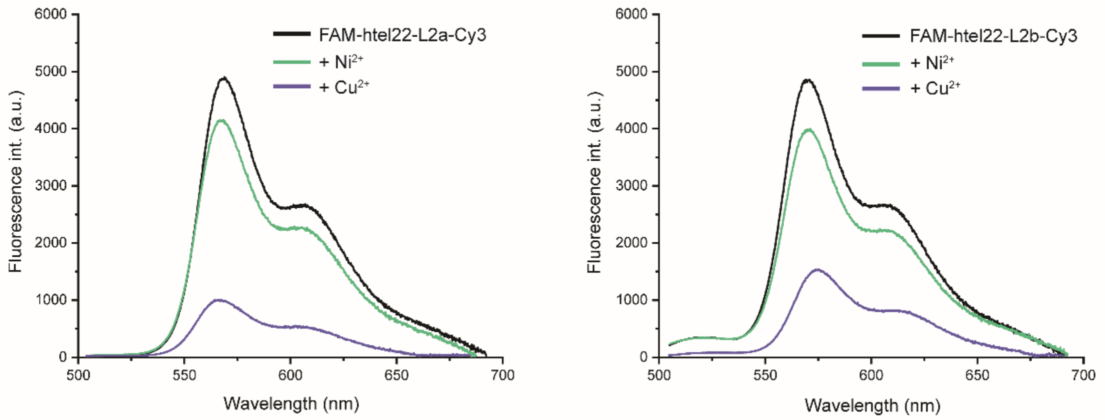

Figure S87. Fluorescence spectra of FAM-htel22-L2a-Cy3 and FAM-htel22-L2b-Cy3 in absence and presence of either Ni^{2+} and Cu^{2+} showcasing metal dependent fluorescence quenching of the modified G-quadruplexes ($\lambda_{ex.}$ = 490nm).

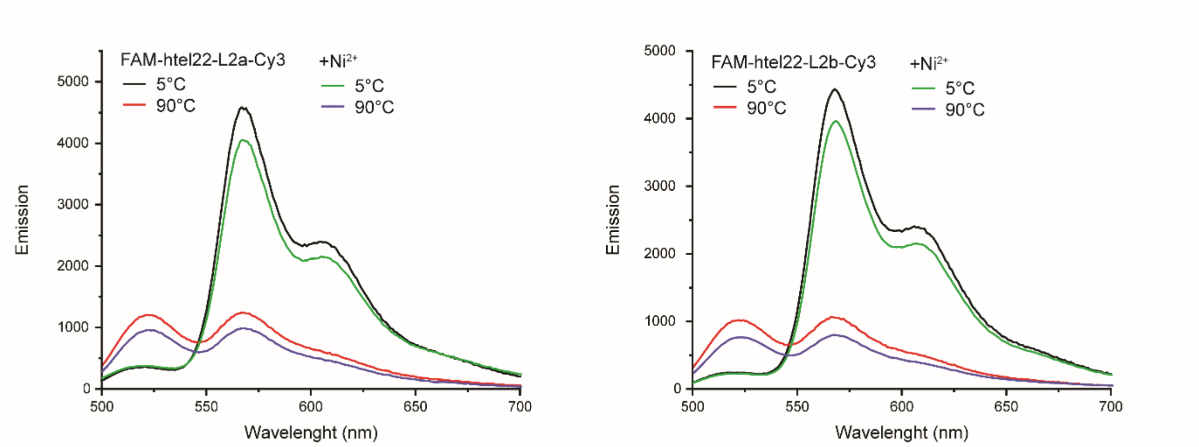

Figure S88. Temperature dependent fluorescence spectra of FAM-htel22-L2a-Cy3 and FAM-htel22-L2b-Cy3 in absence and presence of either Ni^{2+} showing metal dependent as well as temperature dependent fluorescence quenching of the modified G-quadruplexes ($\lambda_{ex.}$ = 490nm).

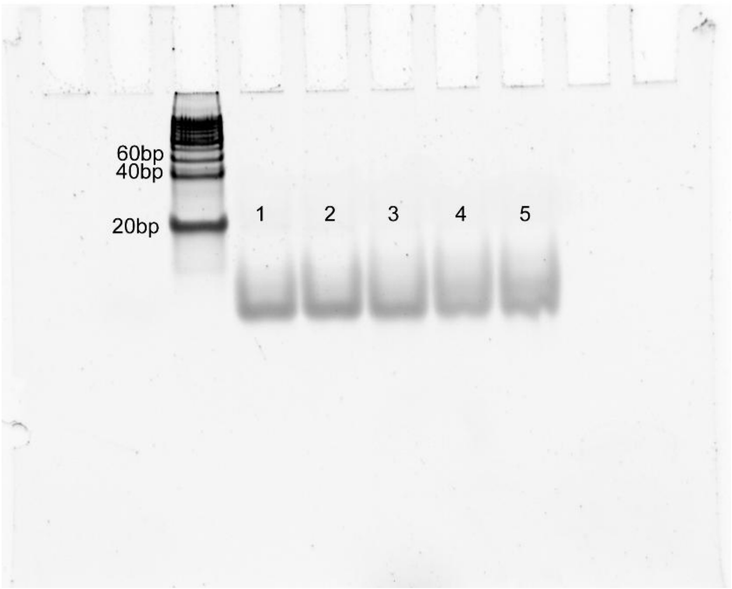

Figure S89. Native polyacrylamide gel electrophoresis (PAGE) experiment of htel22-L2a in presence or absence of increasing concentration of Cu^{2+}. Htel22-L2a was annealed in standard conditions (10uM DNA concentration, 10mM HEPES, 100mM KCl) and in absence or presence of varying equivalents of $CuSO_4$ (0eq, 0.5eq, 1eq, 2eq and 4eq). Final DNA concentration in each well was 8uM. Acrylamide (20%) gel was prepared without EDTA to prevent decomplexation. Running conditions: 90V for 2.5h.Gel was stained post-run with 1X GelGreen® for 45min and visualied using a BioRad ChemiDoc XR+. Lane 1: htel22-L2a, lane 2: htel22-L2a +0.5eq Cu^{2+}, Lane 3: htel22-L2a + 1.0eq Cu^{2+}, Lane 4: htel22-L2a + 2.0eq Cu^{2+}, Lane 5: htel22-L2a + 4.0eq Cu^{2+}. A 20bp DNA ladder was used in lane 0 to give a size estimate. Increased migration of G-quadruplex bands in comparison to dsDNA is expected due to its more compact size.

17. References

[1] D. M. Engelhard, J. Nowack, G. H. Clever, *Angew. Chem. Int. Ed.* **2017**, *56*, 11640–11644.
[2] W. D. Cornell, P. Cieplak, C. I. Bayly, I. R. Gould, K. M. Merz, D. M. Ferguson, D. C. Spellmeyer, T. Fox, J. W. Caldwell, P. A. Kollman, *J. Am. Chem. Soc.* **1995**, *117*, 5179–5197.
[3] C. I. Bayly, P. Cieplak, W. Cornell, P. A. Kollman, *J. Phys. Chem.* **1993**, *97*, 10269–10280.
[4] F.-Y. Dupradeau, A. Pigache, T. Zaffran, C. Savineau, R. Lelong, N. Grivel, D. Lelong, W. Rosanski, P. Cieplak, *Phys. Chem. Chem. Phys.* **2010**, *12*, 7821–39.
[5] E. Vanquelef, S. Simon, G. Marquant, E. Garcia, G. Klimerak, J. C. Delepine, P. Cieplak, F.-Y. Dupradeau, *Nucleic Acids Res.* **2011**, *39*, W511-7.
[6] Y. Wang, D. J. Patel, *Structure* **1993**, *1*, 263–282.
[7] J. Dai, M. Carver, C. Punchihewa, R. A. Jones, D. Yang, *Nucleic Acids Res.* **2007**, *35*, 4927–4940.
[8] J. Dai, C. Punchihewa, A. Ambrus, D. Chen, R. A. Jones, D. Yang, *Nucleic Acids Res* **2007**, *35*, 2440–2450.
[9] E. F. Pettersen, T. D. Goddard, C. C. Huang, G. S. Couch, D. M. Greenblatt, E. C. Meng, T. E. Ferrin, *J. Comput. Chem.* **2004**, *25*, 1605–1612.
[10] U. Essmann, L. Perera, M. L. Berkowitz, T. Darden, H. Lee, L. G. Pedersen, *J. Chem. Phys.* **1995**, *103*, 8577–8593.
